# Protein post-translational modification crotonylation of TXN and GLO1 in artery and vein grafts for coronary artery surgery

**DOI:** 10.1016/j.redox.2025.103608

**Published:** 2025-03-22

**Authors:** Wen-Tao Sun, Huan-Xin Chen, Hai-Tao Hou, Hong-Mei Xue, Qin Yang, Guo-Wei He

**Affiliations:** a Department of Cardiovascular Surgery & The Institute of Cardiovascular Diseases, TEDA International Cardiovascular Hospital, Tianjin University & Chinese Academy of Medical Sciences, Tianjin, China; b Tianjin Key Laboratory of Molecular Regulation of Cardiovascular Diseases and Translational Medicine, Tianjin, China; c School of Life Sciences and Health, University of Health and Rehabilitation Sciences, Qingdao, 266071, China; d Division of Cardiothoracic Surgery, Department of Surgery, Oregon Health and Science University, Portland, OR, USA

**Keywords:** Internal thoracic artery, Saphenous vein, Post-translational crotonylation

## Abstract

A key problem in coronary artery bypass grafting (CABG) is the lower long-term patency of the saphenous vein (SV) compared to internal thoracic artery (ITA). The potential strategies to improve the long-term patency of the vein graft include developing drugs to block unfavorable pathways in the vein and even to change the protein structure of the vein towards arterial structure. It is therefore important to understand the differences of the protein structure between arterial and venous grafts. Using post-translational modification (PTM) proteomics, we systematically investigated differences between ITA and SV with regard to a vascular stenosis-related PTM crotonylation.

Crotonylome and PTM crotonylation in paired ITA and SV segments (n = 150) from patients undergoing CABG surgery were performed by proteomics analysis with further validation. To elucidate the underlying mechanisms, we focused on three crotonylated enzymatic proteins with anti-oxidative effects-thioredoxin (TXN), glyoxalase 1 (GLO1), and glyceraldehyde-3-phosphate dehydrogenase (GAPDH) - whose crotonylation patterns were systematically investigated. The functional validation was performed using both site-mutation experiments in HEK293 cells and pharmacological inhibitors in *ex vivo* cultured ITA/SV tissue specimens.

Comprehensive crotonyl-proteomics demonstrated 3652 proteins are differentially-expressed and 411 proteins are differentially-crotonylated in ITA/SV segments. In the identified crotonylated proteins, SV demonstrated significantly higher levels compared to ITA. Notably, SV showed higher crotonylation levels on TXN-K3, GLO1-K157, and GAPDH-K61, which were associated with decreased enzymatic activity, elevated methylglyoxal (MGO) accumulation, and increased oxidative stress. Inhibition of CREB-binding protein (CBP) reversed oxidative stress in SV by suppressing crotonylation of the three enzymes. In Hek293 cells, both site-specific and comprehensive crotonylation decreased the activities of TXN/GLO1/GAPDH, which in turn triggered the accumulation of MGO. Overexpression of histone deacetylases HDAC1 and HDAC3 showed the opposite effect, restoring enzyme function.

This study is the first to reveal significant differences in PTM crotonylation between human ITA and SV, shedding light on the biological mechanisms underlying the functional disparities between these grafts. These differences impact the enzymatic activity of key proteins involved in oxidative stress, providing insights into the molecular basis of graft performance. Importantly, these findings form a scientific basis for developing specific methods including new anti-oxidative drugs and gene therapy to target on crotonylation in the vein graft in order to improve the long-term graft patency.

## Highlights

• A key problem in CABG is lower long-term patency of the SV compared to ITA but the mechanism is unclear.
• We for the first time studied PTM proteomics in ITA and SV.
• Differences in lysine crotonylation of ITA/SV were found affecting enzymes and oxidative stress.
• CBP may act as a potential target to improve the oxidation resistance in SV graft.

## Graphical abstract

**Figure:**
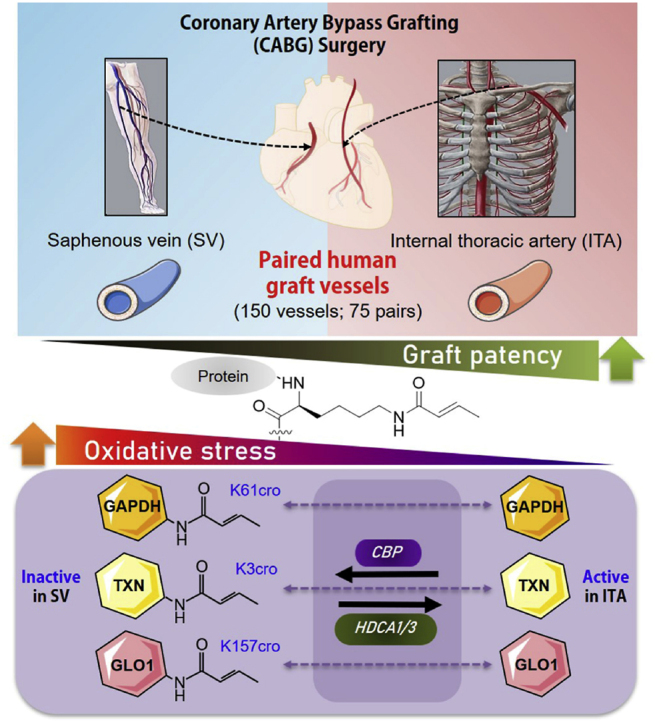

**Table:** Abbreviations

CABG	coronary artery bypass grafting
**CBP**	CREB-binding protein
**GAPDH**	glyceraldehyde-3-phosphate dehydrogenase
**GLO1**	glyoxalase 1
**ITA**	internal thoracic artery
**Kcro**	lysine crotonylation
**MGO**	Methylglyoxal
**PTM**	post-translational modification
**SV**	saphenous vein
**TXN**	thioredoxin

## 1 Introduction

Coronary artery disease, also known as ischemic heart disease, is the leading cause of death worldwide, accounting for 16 % of global mortality according to the World Health Organization. Since 2000, deaths attributed to coronary artery disease have increased by over 2 million, reaching 8.9 million in 2019 [1].

Coronary artery bypass grafting (CABG) is the most frequently performed heart surgery in adults and is the primary surgical intervention for multi-vessel coronary artery disease. Autologous arteries and veins, such as the internal thoracic artery (ITA) and the saphenous vein (SV), are used as graft materials in CABG procedures. The long-term efficacy and survival rate of patients who receive CABG depend on the long-term patency of the vascular graft [2]. What puzzles the clinicians for decades is the significant disparity in the 10-year patency rates between ITA and SV grafts, with 85 %–95 % of ITA grafts remaining functional compared to only 50 %–60 % of SV grafts [[2], [3], [4]]. Some biological characteristics have been revealed to explain the difference of the two grafts [[5], [6], [7]]. ITA grafts exhibit superior endothelial function, including enhanced production and prolonged activity of endothelium-dependent relaxing factors such as nitric oxide (NO⋅) and endothelial-derived hyperpolarizing factors, both of which are essential for maintaining postoperative graft patency [8]. In contrast, SV grafts lack sufficient endogenous antioxidative factors, rendering them more susceptible to intimal hyperplasia triggered by local growth stimuli [9]. In addition, structural differences in the medium layers, especially the production of matrix metalloproteinase [10,11], makes SVs more vulnerable to the pulsatile stretch of blood circulation, which induces pathological changes including smooth muscle proliferation [12], inflammatory cell infiltration, and lipoprotein deposition [13]. These molecular mechanisms, based on well-known functional proteins and enzymes in the vasculature, partially explain the differences in long-term patency between ITA and SV grafts. However, a comprehensive and broad-level understanding of the differences between the ITA and SV conduits is far from adequate.

Protein posttranslational modifications (PTMs) are important epigenetic regulatory mechanisms involved in diverse biological processes. Dysregulation of PTMs at lysine residues (eg, acetylation, ubiquitination, and methylation) is associated with various cardiovascular diseases, including hypertension, vascular dysfunction, and heart failure [14,15]. As a conserved short-chain PTM, lysine crotonylation (Kcro) was first reported in human somatic and mouse male germ cells by Tan in 2011 [16]. Most of the previous studies regarding Kcro PTM focused primarily on histone proteins. Yan et al. recently reviewed crotonylation's role in pan-vascular diseases, demonstrating its involvement in myocardial hypertrophy and cerebral ischemia-reperfusion injury via histone H3 (K18, K12, K9) modifications [15]. Proteomic studies of Kcro modification conducted in both prokaryotic [18] and eukaryotic cells [16,[19], [20], [21], [22]] demonstrated global cellular localizations of non-histone Kcro modifications and their crucial roles in the regulation of cell cycle, inspiration/energy metabolism, and inflammatory responses. A Kcro proteomics study by Cao et al. discovered that crotonylation modifications play a significant role in rat vascular smooth muscle cell phenotypic remodeling [22]. These findings suggest that Kcro PTMs may contribute to vascular remodeling by regulating protein function in bypass vessels.

**Fig. 1:**
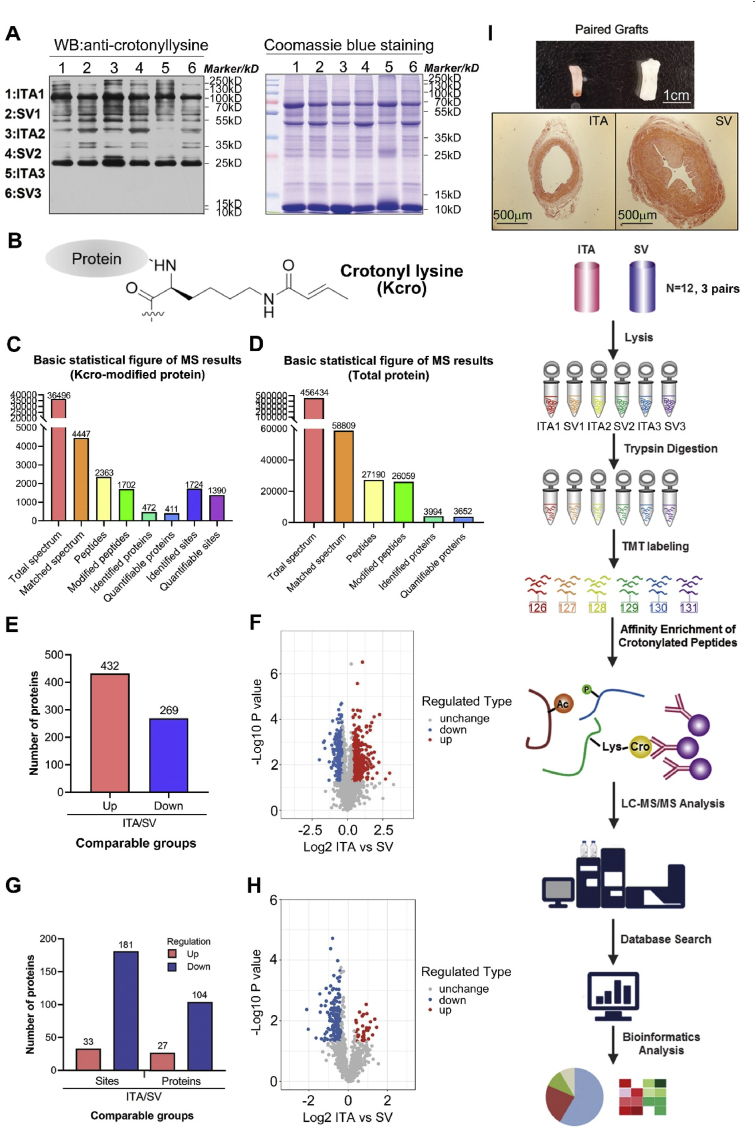
Identification of the Kcro proteins in human ITA and SV grafts. (A) Western blotting with pan anti-crotonyllysine antibody (PTM-502; Lot: 20372671D527; 1:1000 dilution). 20 μg protein/lane. (B) The structure of the crotonaldehyde adduct. (C), (D) Basic statistical table of MS results for crotonylated-proteins and total proteins. (E) Histogram of the number distribution of differentially-expressed proteins in ITA and SV. (F) Volcano plot of differentially expressed proteins. (G) Histogram of the number distribution of differentially crotonylated-proteins and -sites in ITA and SV. (H) Volcano plot of differentially expressed crotonylation sites. (I) Flowchart of crotonylation proteomics.

Structurally, the Kcro modification spans four carbons, featuring a carbon-carbon (C–C) π-bond that confers a distinctive rigid, planar conformation (Fig. 1B) [23]. Levels of protein lysine Kcro could be regulated by the abundance of crotonyl-CoA, which is metabolized from short-chain fatty acid such as crotonate. In addition, the regulation of Kcro involves a dynamic equilibrium maintained by the enzymatic activities of writer and eraser proteins, which are responsible for its addition and removal, respectively [24]. In general, Kcro could be catalyzed by universal lysine acetyltransferases, such as the CREB-binding protein (CBP)/p300 [22,25], and removed by specific histone deacetylases (HDACs), including HDAC1/2/3 and sirtuin1/2/3 [17,26,27]. Recent studies have highlighted the association of HDAC1/3 and SIRT1/3/6 with vasculitis, vascular calcification and atherosclerosis [14,28,29]. However, the presence of Kcro PTMs in human vascular tissues and their role in regulating vascular function remain unknown.

In this study, we first performed lysine crotonylome (crotonylation proteomics) analysis to compare protein Kcro PTMs between human ITA and SV vessels. We further revealed the role of site-specific and CBP-promoted crotonylation in three graft-patency-related enzymes and its impact on oxidative stress in Hek293 cell and in the SV tissue, which may partially explain the different long-term performance between ITA and SV, as well as inspiring new perspectives to the therapeutic strategies to improve outcomes following CABG surgery.

## 2 Methods

### 2.1 Data availability

The data that support the findings of this study are available from the corresponding author upon reasonable request. The mass spectrometry proteomics data have been deposited to the ProteomeXchange Consortium (http://proteomecentral.proteomexchange.org) via the iProX partner repository with the dataset identifier PXD026404.

### 2.2 Collection of human ITA and SV

Human vascular segments (ITA, n = 75; SV, n = 75) were harvested from 75 patients undergoing CABG surgery, including 56 males and 19 females with an average age of 66.4 ± 7.4 years. The indications for CABG in these patients included unstable angina (n = 52), acute non-ST-segment elevation myocardial infarction (n = 13), stable angina (n = 5), acute anterior myocardial infarction (n = 3), and acute ST-segment elevation myocardial infarction (n = 2). Detailed demographic and clinical characteristics of patients are provided in the Supplemental Table S1. The study fully conformed to the principles of the Declaration of Helsinki and the approval to use discarded vessels was obtained from the Institutional Review Board (IRB) of TEDA International Cardiovascular Hospital, Tianjin China (No. [2019]-0516-1).

### 2.3 Crotonylation proteomics in ITA and SV

For the PTM proteomics study, twelve pairs of ITA/SV segments were selected from male patients aged 54–76 years. To fulf ill the requirement of minimum protein amount of PTM proteomics study (≥300mg/sample, ≥3 mg protein/sample), each four ITA or SV segments were randomly selected and combined as one group (resulting in 3 pooled ITA samples, and 3 pooled SV samples). A flowchart of the sample definition was presented in Fig. S1. Six groups of frozen residues (ITA1, SV1, ITA2, SV2, ITA3, and SV3) were grinded by liquid nitrogen into cell powder and then immersed in the lysis buffer (8 M urea, 3 μM TSA, 50 mM NAM, 2 mM EDTA and 1 % Protease Inhibitor Cocktail). The samples were sonicated on ice using a high-intensity ultrasonic processor (Scientz) for three cycles. The remaining debris was removed by centrifugation at 12,000 g at 4 °C for 10 min. The supernatant was collected for concentration determination and trypsin digestion. The amount of protein in each sample was shown in Table S2.

The six samples (ITA1, SV1, ITA2, SV2, ITA3, and SV3) were then incubated with tandem mass tag (TMT) sixplex Isobaric Lable Reagent (Thermo Fisher Scientific, 90068) for 2 h at room temperature, and further pooled, desalted and dried by vacuum centrifugation. The six TMT-labled peptides, dissolved in NETN buffer (100 mM NaCl, 1 mM EDTA, 50 mM Tris-HCl, 0.5 % NP-40, pH 8.0), were incubated overnight at 4 °C with gentle shaking, using pre-washed antibody beads (PTM Bio, PTM503). The peptides were eluted from the beads using 0.1 % trifluoroacetic acid. Finally, the eluted fractions were combined and vacuum-dried. For LC-MS/MS analysis, the resulting peptides were desalted with C18 ZipTips (Millipore) according to the manufacturer's instructions. The enriched crotonylated peptides were analyzed using an EASY-nLC 1000 UPLC system. The peptides were subjected to the NSI source, followed by tandem mass spectrometry (MS/MS) in Q ExactiveTM Plus mass spectrometer (Thermo Scientific), coupled online to the UPLC. These experiments were performed in Jingjie PTM Biolab Co. Ltd, Hangzhou, China. A flowchart of the crotonylation proteomics was presented in Fig. 1I.

### 2.4 Database search

The resulting MS/MS data were processed using MaxQuant search engine (v.1.5.2.8). Tandem mass spectra were searched against the human SwissProt database (20317 entries), concatenated with reverse decoy database. Trypsin/P was specified as the cleavage enzyme, allowing up to 4 missing cleavages. The mass tolerance for precursor ions was set as 20 ppm in First search and 5 ppm in Main search, while the mass tolerance for fragment ions was set as 0.02 Da. Carbamidomethyl on Cys was specified as fixed modification. Acetylation on protein N-terminal, oxidation on Met, deamidation (NQ) and crotonylation on Lys were specified as variable modifications. Quantification was performed using TMT-6plex. A false discovery rate (FDR) was adjusted to < 1 % and minimum score for peptides was set >40.

### 2.5 Identification of up- or down-crotonylated proteins in ITA and SV

The average values of each sample (ITA1, SV1, ITA2, SV2, ITA3, and SV3) were obtained from three repeated experiments. The ratios of the average values between the ITA and SV groups (ITA1 vs. SV1, ITA2 vs. SV2, ITA3 vs. SV3) were calculated. For statistical analysis, the log2 of each value was taken for the p-value calculation from two-tailed T-test. A threshold of p-value <0.05 was applied, and ratios of ITA/SV greater than 1.3 or less than 1/1.3 were identified as significant up- or down-regulation, respectively (Data Set S2). Notably, all Kcro-proteomics data reported in this study have been normalized by the general proteomics results of the same samples. Details of the proteomics data and Kcro proteomics data can be found in Data Set S1 and Data Set S2, respectively. The details of protein quantification were explained in the Supplementary Methods.

### 2.6 Immunoprecipitation (IP)-Western blot for validation of Kcro-proteins

In a new cohort of 32 patients, the paired ITA-SV samples were collected, and the different protein expression of the identified Kcro-proteins were validated via immunoprecipitation (IP)-Western blot. Details of the procedures have been reported in our prior studies [30,31]. For the validation study, 49 pairs of ITA/SV segments were used. Immunoprecipitation was conducted according to the manufacturer's instructions (Thermo Scientific). Briefly, ITA and SV segments were lysed by IP Lysis/Wash Buffer (pH 7.4, 25 mM Tris, 150 mM NaCl, 1 mM EDTA, 1 % NP40, 5 % glycerol) containing protease and deacetylase inhibitor cocktail (Thermo Scientific & Beyotime) for 10 min on ice. After centrifuge at 12,000 g at 4 °C for 10 min, the supernatant was collected, and protein concentration was determined using a BCA kit according to the manufacturer's instructions. Cell lysate with no less than 1 mg of total protein was incubated with antibody-crosslinked A/G magnetic beads overnight at 4 °C. After washing with elution buffer, the bound antigen was denatured and subjected to SDS-PAGE, followed by immunoblotting with the indicated antibodies. Details of the antibodies used in the study can be found in the Supplementary Methods.

### 2.7 Cell culture, inhibitors and plasmids

The human embryonic kidney cell line HEK293 was cultured in minimum essential medium (MEM), supplemented with 10 % fetal bovine serum (FBS), 100 units/ml penicillin and 100 mg/ml streptomycin at 37 °C with 5 % (v/v) CO_2_. The CBP/p300 inhibitor C646 was purchased from Abcam. The cDNA of GAPDH, GAPDH-FLAG, GAPDH-K61R-FLAG, TXN, TXN-FLAG, TXN-K3R-FLAG, GLO1, GLO1-FLAG, GLO1-K157R-FLAG, HDAC1 and HDAC3 were purchased from GenePharm (Shanghai, China) and the cDNA plasmid of CBP was generously provided by Laboratory of Molecular Cell Biology and Tumor Biology, School of Basic Medical Sciences, Peking University Health Science Center.

### 2.8 TXN activity assay

HEK293 cells (1 × 10^6^)/ITA and SV segments were homogenized with 100 μL of TE Buffer (50 mM Tris, 1 mM EDTA, 0.1 mg/ml bovine serum albumin, pH 7.5) and sonicated on ice for 10 min. The mixture was then centrifuged at 10,000 g for 20 min at 4 °C to remove insoluble material. The supernatant was quantified for protein concentration and used for the TXN assay following the manufacturer's instructions (IMCO Corporation FkTRX-02-V2, Sweden). In brief, the samples were mixed with thioredoxin reductase, β-NADPH and Eosin-labeled insulin in 200 μl tubes. The fluorescence emission at 545 nm (E545) after 520 nm excitation was measured by a fluorescent reader (Roche LightCycler96, USA) in a kinetic mode for 60 min at 37 °C. The increasing fluorescence intensity was calculated during the reaction period, which remained within a linear range. The concentration of TXN at each time-point could be calculated according to the E545 and the standard curve.

### 2.9 GLO1 activity assay

HEK293 cells (1 × 10^6^)/ITA and SV segments were homogenized with 300 μL of ice-cold GLO1 Assay Buffer containing PMSF and kept on ice for 10 min, followed by centrifuge at 12,000 g for 10 min at 4 °C to remove insoluble material. The supernatant was quantified for protein concentration and used for the GLO1 assay following the manufacturer's instructions (Abcam ab241019, UK). In brief, the samples were mixed with substrate mix in wells of a 96 well plate and the absorbance at 240 nm (A240) was measure using a multilabel plate reader (Tecan Spark, Austria) in a kinetic mode for 18 min at room temperature. The activity of GLO1 was calculated based on the formation of S-d-lactoylglutathione (SLG, μmol) within 18 min, which could be determined by measuring the change in A240.

### 2.10 Methylglyoxal (MGO) assay

HEK293 cells (1 × 10^7^)/ITA and SV segments were homogenized with 200 μL of RIPA Buffer (1 × PBS, 1 % NP40, 0.5 % Sodium deoxycholate, 0.1 % SDS) and kept on ice for 20 min, followed by centrifuge at 12,000 g for 10 min at 4 °C to remove insoluble material. The supernatant was quantified for protein concentration and used for the MGO assay following the manufacturer's instructions (Abcam ab273284, UK). In brief, the samples were mixed with enzyme-coupled reaction buffer and the fluorogenic probe at room temperature for 3 h and the fluorescence was measured at 535/587 nm (E587) by a fluorescent reader (Roche LightCycler96, USA). The concentration of MGO at end-point could be calculated based on the E587 value and the standard curve.

### 2.11 GAPDH activity assay

HEK293 cells (1 × 10^6^)/ITA and SV segments were homogenized with 100 μL of ice-cold GAPDH Assay Buffer and kept on ice for 10 min, followed by centrifuge at 10,000 g for 5 min at 4 °C to remove insoluble material. The supernatant was quantified for protein concentration and used for the GAPDH assay following the manufacturer's instructions (Sigma-aldrich MAK277). In brief, the samples were mixed with master reaction mix in wells of a 96 well plate and the absorbance at 450 nm (A450) was measured by a spectrophotometer (Tecan Sunrise, Austria) in a kinetic mode for 60 min at 37 °C. The concentration of NADH at each time-point was calculated based on A450 value and the standard curve. Converted NADH production at two selected time points (T1 & T2) could be applied to calculate the GAPDH activity of the samples.

### 2.12 Protein carbonyl assay

ITA/SV segments were lysed by IP Lysis/Wash Buffer (pH 7.4, 25 mM Tris, 150 mM NaCl, 1 mM EDTA, 1 % NP40, 5 % glycerol) containing a protease and deacetylase inhibitor cocktail (Thermo Scientific & Beyotime) for 10 min on ice. After centrifuge at 12,000 g at 4 °C for 10 min, the supernatant was quantified for protein concentration and used for the protein carbonyl assay following the manufacturer's instructions (Nanjing Jiancheng A087-1). In brief, the samples were mixed with reagents step by step in wells of a 96 well plate and the absorbance at 370 nm (A370) was measured by multilabel plate reader (Tecan Spark, Austria) at 37 °C. The concentration of protein carbonyl was calculated based on the A450 value and the standard curve.

### 2.13 Malondialdehyde (MDA) assay

ITA/SV segments were lysed by IP Lysis/Wash Buffer (pH 7.4, 25 mM Tris, 150 mM NaCl, 1 mM EDTA, 1 % NP40, 5 % glycerol) containing a protease and deacetylase inhibitor cocktail (Thermo Scientific & Beyotime) for 10 min on ice. After centrifuge at 12,000 g at 4 °C for 10 min, the supernatant was quantified for protein concentration and used for the protein carbonyl assay following the manufacturer's instructions (Nanjing Jiancheng A003-2). In brief, the samples were mixed with mix reagents in wells of a 96 well plate and boiled at 95 °C for 40 min. The absorbance was at measured at 532 nm (A532) by multilabel plate reader (Tecan Spark, Austria) at 37 °C. The concentration of protein carbonyl was calculated based on the A450 value and the standard curve.

### 2.14 Validation via enzymatic activity assay and molecular docking

The effects of site-specific crotonylation on protein functions and the roles of CBP, HDAC1 and HDAC3 were examined using Hek293 cellular models. The activities of TXN, GLO1, GAPDH, as well as the concentration of MGO, were measured following the manufacturer's instructions. Moreover, to compare the anti-oxidative potential between the ITA and SV graft samples, the activities of TXN, GLO1 and GAPDH, along with MGO accumulation, protein carbonyl level, and MDA level were assessed in paired ITA and SV by using commercial kits. The beneficial effects of C646, a CBP inhibitor, on the aforementioned factors of SV were also evaluated.

Molecular docking simulations were performed using AutoDock 4.0 to study the binding of small molecules (substrates) to their enzyme structures, with or without crotonylation modification. In this study, the bindings of three systems including TXN - nicotinamide adenine dinucleotide phosphate [NADP (+)], GLO1 - (R)-S-lactoylglutathione, and GAPDH - NAD (+) were simulated. Detailed methods and the Major Resources Table are presented in the Supplementary Methods.

To ensure data robustness and reproducibility, the validation step used the samples of paired ITA and SV from a new cohort of patients. Crotonylation sites were further validated by point-mutations.

### 2.15 Statistical analysis

Protein expression of targets of interest was normalized to the expression of β-actin or β-tubulin. Data were expressed as mean ± SEM. Student's t-test and one-way ANOVA (SPSS, version 20) were used for statistical analysis when appropriate. Sheffe test was used as post-hoc test. p < 0.05 was considered statistically significant.

## 3 Results

### 3.1 Crotonylation proteomics quantified different proteins in human ITA and SV

In both human ITA and SV, proteins were broadly crotonylated with different classification and intensity, as demonstrated by the Western blot analysis using anti-pan-Kcro (Fig. 1A). In paired ITA/SV, we identified 3994 proteins via proteomics, within which 3652 proteins could be quantified as differentially-expressed on the whole protein level (Fig. 1D & Data Set S1). Through Kcro-proteomics, a total of 1724 differentially-expressed crotonylation sites were identified on 472 proteins, of which 1390 Kcro sites on 411 proteins were quantifiable. (Fig. 1C & Data Set S2). The repeatability of quantification was statistically validated across three independent groups of ITA/SV samples (Fig. S4 & Supplemental Methods). Interestingly, crotonylated proteins with a ratio greater than 1.3 between ITA and SV were predominantly non-histone proteins. These results indicated that a large number of proteins are modified by crotonylation in both human ITA and SV graft samples.

On the whole-protein level, 432 proteins were found to be more abundant in ITA, while 269 proteins being higher-expressed in SV (Fig. 1E). In terms of Kcro-modified proteins in ITA/SV, 27 proteins (33 sites) exhibited higher modification in the ITA, whereas 104 proteins (181 sites) showed lower modification in the ITA (Fig. 1G). The distribution of the ratios and p-values for the differentially-expressed proteins and Kcro sites were presented in Fig. 1F and H, respectively.

To identify any specific amino acid biases adjacent to Kcro sites, we analyzed the flanking sequences of these sites using MoMo software. The results showed that phenylalanine (F) residues were overrepresented at both the −1 and + 1 positions surrounding the Kcro sites. Additionally, other amino acids, including alanine (A), aspartic acid (D), glutamic acid (E), and lysine (K), were also frequently enriched at the upstream or downstream of the Kcro sites (Fig. S2A). The top seven most frequent sequences surrounding the crotonylation sites were statically sorted by the motif score (Fig. S2B).

### 3.2 Functional annotation and enrichment analysis of the differentially-crotonylated proteins in ITA and SV

**Fig. 2:**
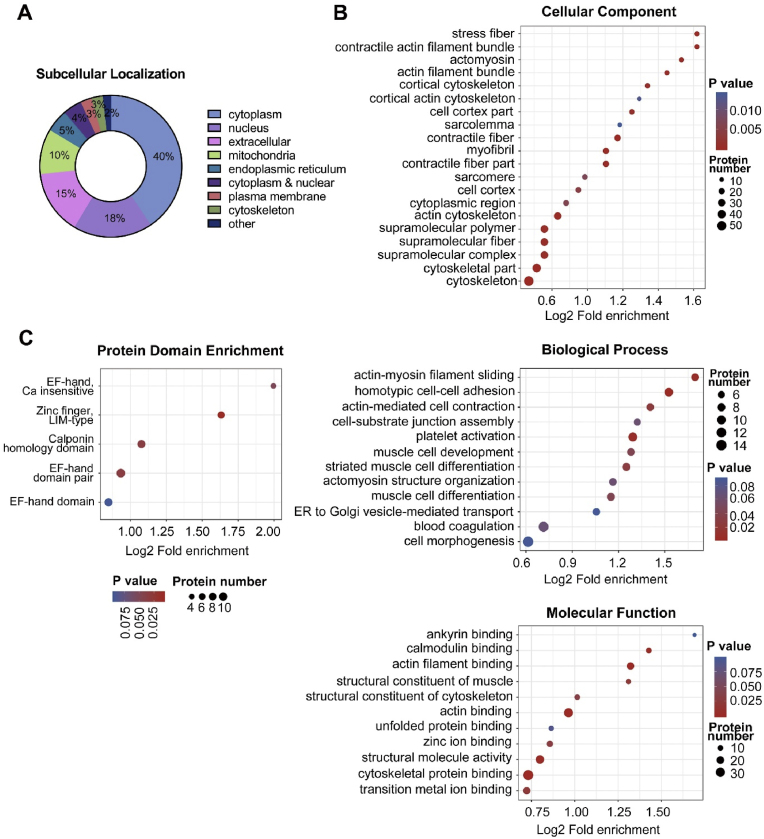
Functional annotation and enrichment of differentially-crotonylated proteins in human ITA and SV grafts. (A) Subcellular localization of the proteins. (B) GO enrichment bubble plot of proteins corresponding to differentially expressed modification sites in three categories (Biological Process; Molecular Function; Cellular Component). (C) Protein domain enrichment bubble plot of proteins corresponding to differentially expressed modification sites.

The proteins were classified according to their subcellular localization, cellular component, biological processes, and molecular function. Specifically, the Kcro-proteins were found in the cytoplasm, nucleus, and extracellular space, indicating broad differences between ITA and SV (Fig. 2A). Moreover, gene ontology (GO) analysis further revealed that these proteins were distributed scatteredly in the cellular components, including intracellular fibers, the cytoskeleton, and cell-movement related proteins (Fig. 2B *above* & Fig. S5a). Besides, these proteins were involved in various biological processes such as actin-myosin filament sliding, cell morphogenesis and cell-cell adhesion (Fig. 2B *middle* & Fig. S5b). Particularly, molecular function analysis implied substantial differences in protein crotonylation related to protein binding, catalytic activity, and structural molecular activity between ITA and SV (Fig. 2B *below* & Fig. S5c). These findings were further supported by the functional classification from the Clusters of Orthologous Groups of proteins (COG/KOG) database, which provided an additional perspective, emphasizing differences in cytoskeleton, signal transduction, and energy metabolism between ITA and SV (Fig. S5d).

**Fig. 3:**
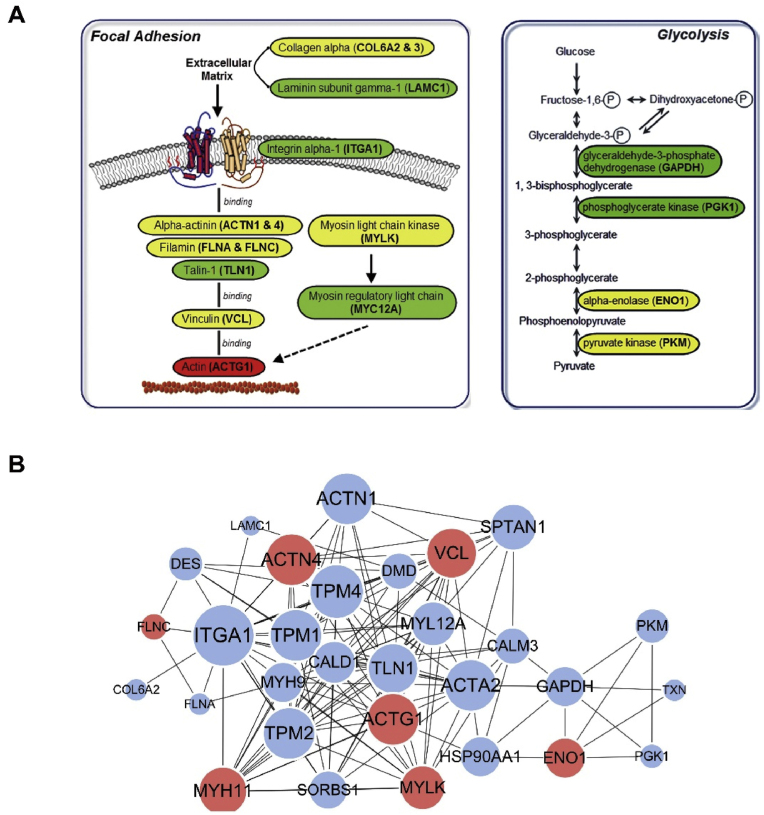
KEGG pathway enrichment and protein-protein interaction network diagram of proteins exhibiting remarkable Kcro differentiation in human ITA and SV grafts. (A) Representative KEGG pathways enriching the differentially-crotonylated proteins. Green: down-regulated protein; Red: up-regulated protein; Yellow: proteins with both up-regulated and down-regulated sites (ITA/SV). (B) The interaction networks of the top 30 most close related proteins with protein IDs based on STRING database. Blue: down-regulated protein; Red: up-regulated protein (ITA vs SV). The circle size represents the number of differentially modified proteins and their interacting proteins. Larger circle means the protein has more interacting proteins, indicating that the protein is more important in the network.

Functional enrichment analysis, including GO enrichment, Kyoto Encyclopedia of Genes and Genomes (KEGG) pathway, and protein domain enrichment, were conducted to further investigate the shared functions of these differentially-crotonylated proteins. Protein domain enrichment indicated that these proteins were enriched in EF-hand domain, Zinc finger and Calponin homology domain (Fig. 2C). Consistent with the GO enrichments, KEGG enrichments also demonstrated differential Kcro-modification of proteins involved in tight junction and focal adhesion (Fig. 3A *left panel* & Fig. S5e). In addition, various metabolic enzymes in glycolysis were differentially-crotonylated between ITA and SV (Fig. 3A *right panel*). A protein-protein interaction network was achieved by comparison of proteins with the STRING database (v.10.5), indicating the important proteins in the network, such as integrin alpha-1 (ITGA1), alpha-actinin (ACTN4), actin (ATCG1), tropomyosin alpha-1 (TPM1), alpha-enolase (ENO1), and GAPDH (Fig. 3B). Results of these functional annotations and enrichment analyses suggested that the differentially expressed, crotonylated proteins in ITA and SV are widely distributed across various tissues and play critical roles in a range of cellular processes, particularly in the cell structure/motion/differentiation and energy metabolism. More detailed information about the correlations between the modification multiples and site function was shown in the heatmaps related to GO enrichment, KEGG pathway and protein domain (Fig. S6).

### 3.3 Validation of the differentially-expressed Kcro-proteins in ITA/SV

**Fig. 4:**
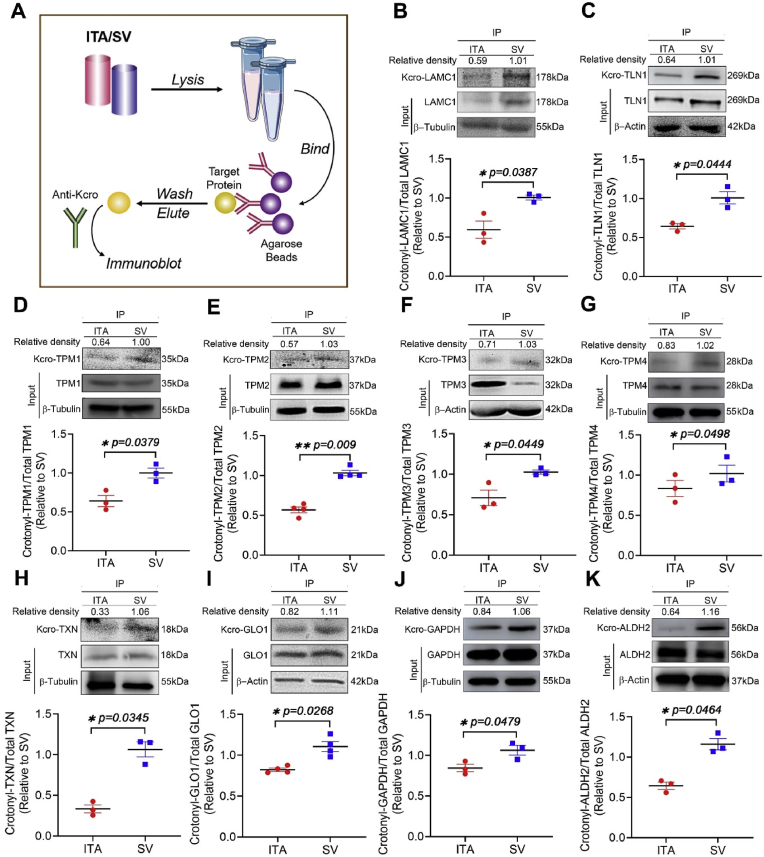
Representative blots of the differentially-crotonylated proteins in ITA/SVs. (A) Flowchart of IP-western blot for examining the protein crotonylation. (B) Statistical evaluation and representative blot of the total and crotonylated LAMC1 in human ITA and SV. Tissue lysates were immunoprecipitated with an anti-LAMC1 antibody, followed by immunoblotting with a pan-Kcro antibody. Each column represents mean ± S.E. (n = 3 or 4 independent replicates). The ratio of Kcro-LAMC1/total LAMC1 in ITA was normalized with that in SV. Paired *t*-test, ∗p < 0.05, ∗∗p < 0.01 vs. ITA. (C)–(K), validation of the total and crotonylated TLN1, TPM1, TPM2, TPM3, TPM4, TXN, GLO1, GAPDH and ALDH2 in human ITA and SV. Three or four independent pairs of ITA/SV grafts were examined for each target protein.

To validate the differentially-expressed crotonylated proteins identified by our MS data, we selected 10 representative protein that exhibited remarkably differences in the crotonylation ratio of ITA/SV, and are well-documented for their involvement in regulating vascular function. The crotonylation status of these proteins were examined by immunoprecipitation (IP)-western blot in the newly-collected 32 pairs of ITA/SV. These proteins include laminin subunit gamma-1 (LAMC1), talin-1 (TLN1), TPM1, tropomyosin beta chain (TPM2), tropomyosin alpha-3 chain (TPM3), tropomyosin alpha-4 chain (TPM4), TXN, GLO1, GAPDH, and mitochondrial aldehyde dehydrogenase (ALDH2). These selected proteins were involved in various regulatory pathways of vascular function, including focal adhesion, anti-oxidation, glycolysis, endothelial NO⋅ production, neointima formation and smooth muscle cell dedifferentiation (Table S3). The expression and crotonylation level of these proteins in the new cohort of ITA/SV sample were demonstrated to be consistently with the MS results (Fig. 4B–K).

### 3.4 Site-specific lysine crotonylation deactivates antioxidant proteins to induce MGO accumulation

**Fig. 5:**
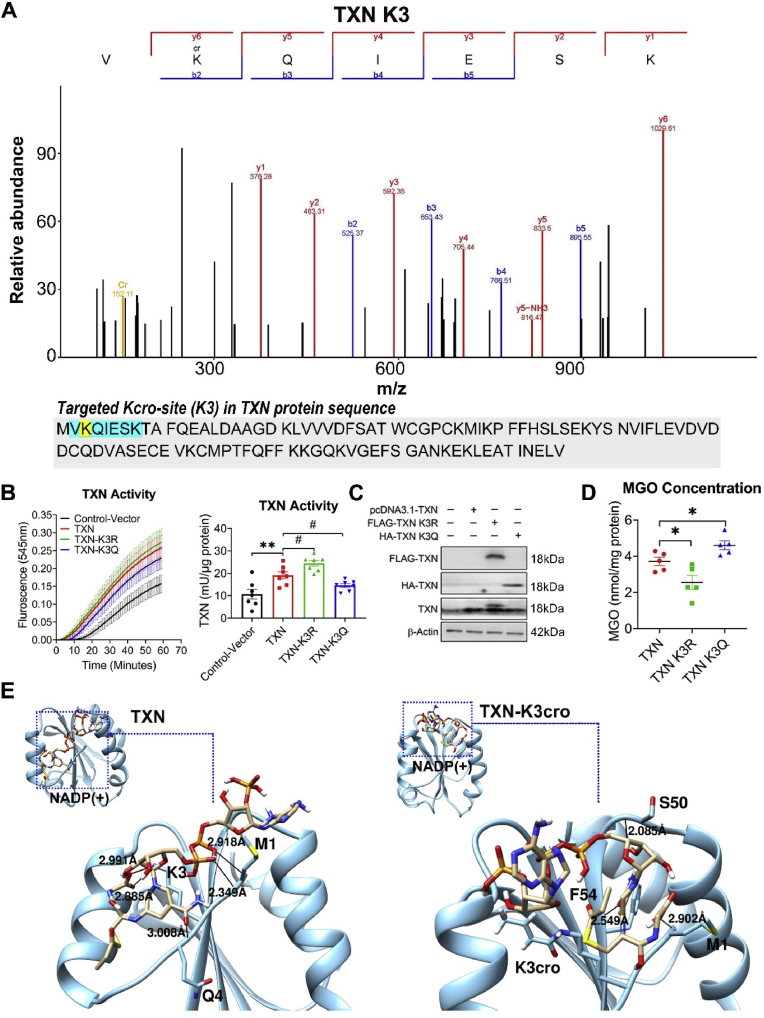
Crotonylation decreases TXN activity in Hek293 cell. (A) Mass spectrometry of ITA/SV at TXN K3 site. (B) Influence of K3-point mutation on the TXN activity. *Left panel:* Time-dependent line graph of TXN activity assay in Hek293 cell. Each spot represents mean ± S.E. (n = 7 independent replicates). *Right panel*: Histogram of TXN activity quantified by the slope of the time-dependent line. Each bar represents mean ± S.E. (n = 7 independent replicates); one-way ANOVA, ∗∗P < 0.01 vs. control; #P < 0.05 vs. TXN group. (C) Validation of point-mutation on the protein level. (D) Influence of TXN-K3-point mutation on the MGO concentration. Each bar represents mean ± S.E. (n = 5 independent replicates); one-way ANOVA, ∗P < 0.05 vs. TXN group. (E) Simulation of the binding conformation of TXN with its substrate NADP (+). The black line represents the hydrogen bond between the protein and its substrate.

**Fig. 6:**
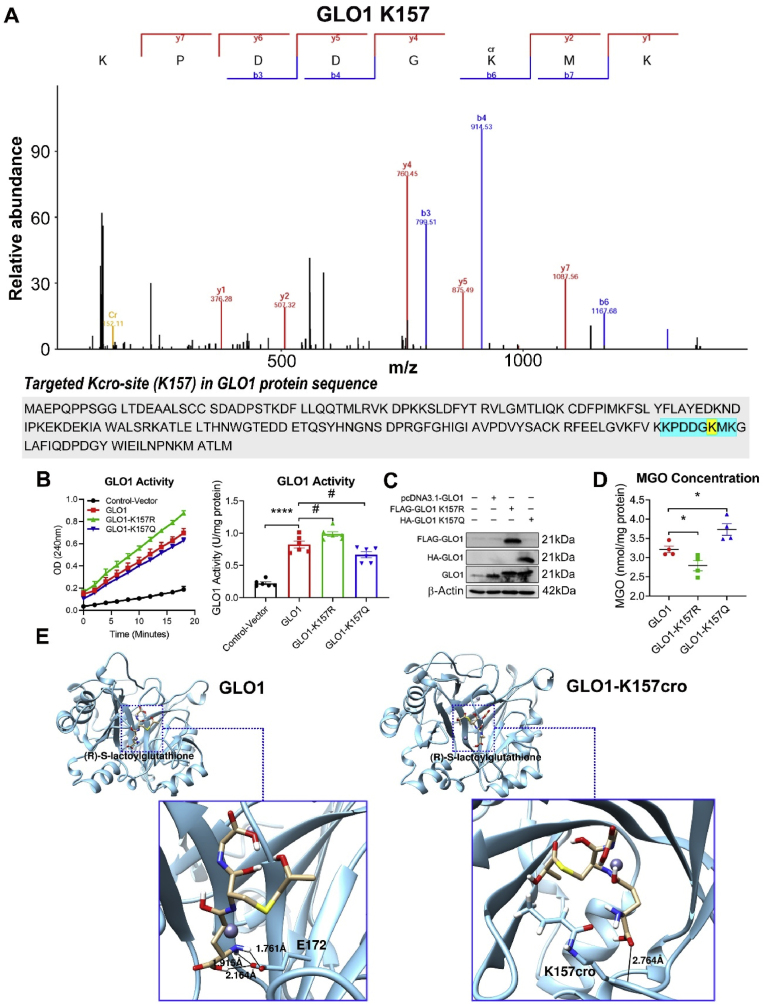
Crotonylation decreases GLO1 activity in Hek293 cell. (A) Mass spectrometry of ITA/SV at GLO1 K157 site. (B) Influence of K157-point mutation on the GLO1 activity. *Left panel:* Time-dependent line graph of GLO1 activity assay in Hek293 cell. Each spot represents mean ± S.E. (n = 6 independent replicates). *Right panel*: Histogram of GLO1 activity quantified by the slope of the time-dependent line. Each bar represents mean ± S.E. (n = 6 independent replicates); one-way ANOVA, ∗∗∗∗P < 0.0001 vs. control; #P < 0.05 vs. GLO1 group. (C) Validation of point-mutation on the protein level. (D) Influence of GLO1-K157-point mutation on the MGO concentration. Each bar represents mean ± S.E. (n = 4 independent replicates); one-way ANOVA, ∗P < 0.05 vs. GLO1 group. (E) Simulation of the binding conformation of GLO1 with its substrate (R)-S-lactoylglutathione. The black line in the blue box represents the hydrogen bond between the protein and its substrate.

**Fig. 7:**
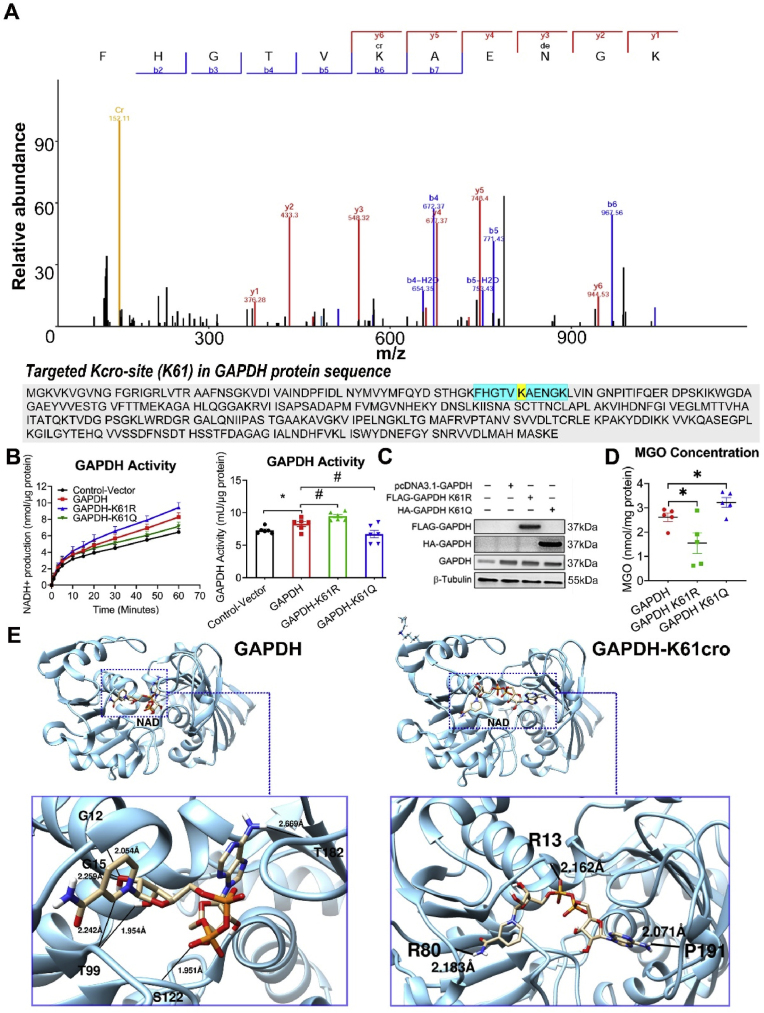
Crotonylation decreases GAPDH activity in Hek293 cell. (A) Mass spectrometry of ITA/SV at GAPDH K61 site. (B) Influence of K61-point-mutation on the GAPDH activity. *Left panel:* Time-dependent line graph of GAPDH activity assay in Hek293 cell. Each spot represents mean ± S.E. (n = 6 independent replicates). *Right panel*: Histogram of GAPDH activity quantified by the slope of the time-dependent line. Each bar represents mean ± S.E. (n = 6 independent replicates); one-way ANOVA, ∗P < 0.05 vs. control; #P < 0.05 vs. GAPDH group. (C) Validation of K61-point-mutation on the protein level. (D) Influence of GAPDH-K61-point mutation on the MGO concentration in Hek293 cell. Each bar represents mean ± S.E. (n = 5 independent replicates); one-way ANOVA, ∗P < 0.05 vs. GAPDH group. (E) Simulation of the binding conformation of GAPDH with its substrate NAD (+). The black line in the blue box represents the hydrogen bond between the protein and its substrate.

To investigate the impact of site-specific crotonylation on protein function, a validation study on three measurable antioxidant proteins (TXN, GLO1 and GAPDH) were conducted in Hek293 cellular models. The differentially-crotonylated sites of TXN (K3), GLO1 (K157), and GAPDH (K61) were identified from the proteomics data (Fig. 5, Fig. 6, Fig. 7A, Table S3).

Site-mutation on TXN-K3R (Lysine to Arginine), which mimicked the de-crotonylation on site 3, enhanced TXN activity and reduced the concentration of MGO, a toxic glycolytic byproduct which leads to vascular dysfunction. In contrast, the TXN-K3Q mutation (Lysine to Glutamine), which mimicked the 100 % crotonylation on site 3, diminished TXN activity and increased the concentration of MGO (Fig. 5B–D). The results suggested an adverse role of K3-crotonylation on TXN activity. Molecular docking analysis (Fig. 5E) showed that, in the absence of Kcro, NADP (+) bounded to the substrate binding site, which included residues M1, K3, and Q4, with a binding energy of −3.44 kcal/mol (*left panel*). However, in the presence of Kcro, NADP (+) bounded to the neighboring site composed of M1, S50, and F54, with a reduced binding energy of −0.72 kcal/mol (*right panel*). This suggested that K3 crotonylation disrupts the binding stability of TXN and its substrate, NADP (+), via weakening hydrogen bond formation and altering the binding energy, ultimately leading to reduced enzymatic activity of TXN.

Similarly, site-specific mutation of GLO1 at K157R, which mimicked de-crotonylation at site 157, enhanced GLO1 activity (Fig. 6B and C) and led to a reduction in MGO levels (Fig. 6D). In contrast, GLO1-K157Q, which mimicked full crotonylation at site 157, decreased GLO1 activity and increased MGO levels (Fig. 6B, C & D). Molecular docking analysis (Fig. 6E) showed that, in the absence of Kcro, a tight junction consisting three hydrogen bonds were formed between (R)-S-lactoylglutathione and E172, with a binding energy of −5.62 kcal/mol (*left panel*). In the presence of Kcro, the substrate formed a weaker interaction with K157cro, with only one hydrogen bond and a reduced binding energy of −3.61 kcal/mol (*right panel*). These results suggested that K157 crotonylation weakens the binding stability of GLO1 and (R)-S-lactoylglutathione, the substrate, via disrupting the formation of hydrogen bonds and altering the binding conformation, ultimately leading to reduced enzymatic activity of GLO1.

Furthermore, site-mutation of GAPDH-K61R promoted GAPDH activity and decreased the concentration of MGO (Fig. 7B–D). In contrast, GAPDH-K61Q showed the opposite effects, suggesting the adverse role of 61K crotonylation on GAPDH activity. Molecular docking analysis (Fig. 7E) demonstrated that in the absence of Kcro, NAD (+) bounded to the substrate binding site, which included G12, G15, T99, S122, and T182, forming 6 hydrogen bonds with a binding energy of −7.65 kcal/mol (*left panel*). In the presence of Kcro, NAD (+) shifted to a neighboring site consisting of R13, R80, and P191, forming only 3 hydrogen bonds, with a binding energy of −6.85 kcal/mol (*right panel*). These findings suggested that K61 crotonylation reduces the binding stability between GAPDH and NAD (+), the substrate, via breaking hydrogen bond formation and modifying the binding energy, which might eventually reduce the enzymatic activity of GAPDH.

### 3.5 CBP-mediated crotonyl-modification deactivates antioxidant proteins to induce MGO accumulation and stimulate oxidative stress

**Fig. 8:**
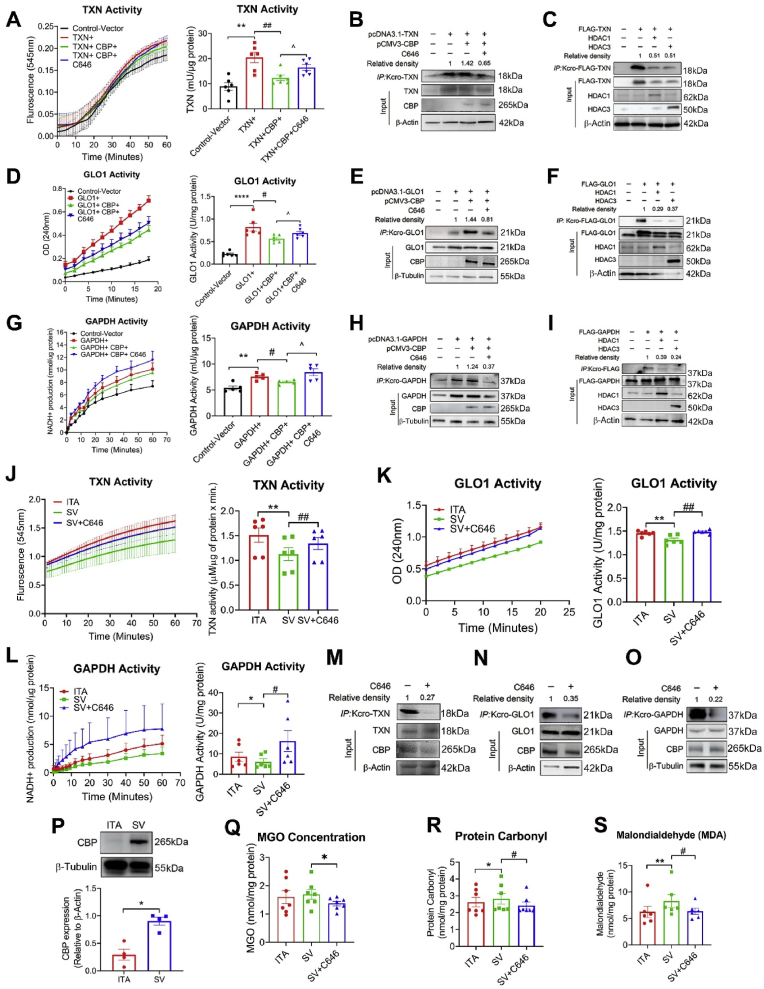
Effect of crotonylation/de-crotonylation modification on the activities of TXN, GLO1 and GAPDH in Hek293 cells and on the oxidative stress in the SV graft. (A)(D)(G) Effect of CBP and the inhibitor C646 on TXN/GLO1/GAPDH activity in Hek293. Each spot/bar represents mean ± S.E. (6 independent replicates in TXN and GLO1 group, 5 independent replicates in GAPDH group); one-way ANOVA, ∗∗P < 0.01; ∗∗∗∗P < 0.0001 vs. control; ##P < 0.01; #P < 0.05; ^P < 0.05. (B)(E)(H) Effect of CBP and the inhibitor C646 on the crotonylation level of TXN/GLO1/GAPDH. (C)(F)(I) Effect of HDAC1 and HDAC3 on the de-crotonylation of TXN/GLO1/GAPDH. (J–L) TXN/GLO1/GAPDH activity in SV is lower than in ITA, and C646 enhanced TXN/GLO1/GAPDH activity. 6 independent replicates in TXN, GLO1 and GAPDH group; one-way ANOVA, ∗P < 0.05; ∗∗P < 0.01 vs. ITA; #P < 0.05; ##P < 0.01 vs. SV. (M–O) Incubation of C646 decreased TXN/GLO1/GAPDH crotonylation in SV. (P) The CBP expression in SV is higher than in ITA. n = 5 independent replicates; paired *t*-test, ∗P < 0.05 vs. ITA. (Q) MGO level in SV and in ITA is similar, and C646 decreases MGO level in SV. n = 7 independent replicates; one-way ANOVA, ∗P < 0.05 vs. SV. (R) Protein carbonyl in SV is higher than in ITA, and C646 decreased the content of protein carbonyl. n = 7 independent replicates; one-way ANOVA, ∗P < 0.05 vs. ITA; #P < 0.05 vs. SV. (S) Malondialdehyde (MDA) in SV is higher than in ITA, and C646 decreased the content of MDA. n = 6 independent replicates; one-way ANOVA, ∗∗P < 0.01 vs. ITA; #P < 0.05 vs. SV. Each spot/bar represents mean ± S.E.

Whole-cell levels of crotonylated TXN, GLO1 and GAPDH in Hek293 cells were found to be upregulated by CBP and downregulated by the inhibitor C646 (Fig. 8B, E & 8H), along with a corresponding reverse regulation of their enzymatic activity (Fig. 8A, D & 8G). In addition, the de-crotonylation of TXN, GLO1 and GAPDH was catalyzed by histone deacetylase (HDAC)1 and HDAC3 (Fig. 8C, F & 8I).

To further investigate the role of CBP in regulating antioxidant protein activity, we examined its effect in ITA and SV. Comparing with ITA, SV showed a higher level of CBP, reduced enzyme activities of TXN, GLO1 and GAPDH, and more pronounced oxidative stress, as indicated by elevated protein carbonyl and malondialdehyde (MDA) content (Fig. 8J–L & P-S). Incubation of SV with C646 led to a decrease in the crotonylation levels of TXN, GLO1 and GAPDH, which in turn restored their activities, reduced MGO concentration, and alleviated oxidative stress in the vessels (Fig. 8J–O & Q-S). These results suggested that CBP negatively impacts the antioxidant properties of the vessels by regulating protein lysine crotonylation.

## 4 Discussion

In this study, we employed crotonylation proteomics to identify and quantify lysine crotonylated proteins (Kcro-proteins), along with cell and tissue enzymatic assays for functional validation, to compare, for the first time, the profiling and mechanisms of lysine crotonylation in human arteries and veins. Results revealed that: (1) lysine crotonylation exists in both ITA and SV, with a significant difference in the crotonylated protein profiles between the two; (2) most of the differentially-crotonylated proteins are non-histone, and are involved in cellular functions such as structural integrity, motility, differentiation, and energy metabolism; (3) crotonylation, mediated by CBP, negatively regulates the activity of antioxidant proteins (TXN, GLO1, and GAPDH), leading to the accumulation of methylglyoxal (MGO) and the induction of oxidative stress in both cellular and vascular models. These findings suggest that CBP-mediated lysine crotonylation may contribute to the functional differences and varying patency outcomes observed between arterial and venous grafts in coronary artery bypass graft (CABG) surgery.

In the early, mid-term and late stages following graft implantation, various proteins and enzymes contribute to the pathophysiology of endothelial cell function, smooth muscle cell growth, and extracellular matrix deposition, ultimately causing tube occlusion and diminishing long-term patency [2,32]. Recent advances in proteomics techniques have enabled comprehensive characterization of the CABG grafts in both physiological and disease states, facilitating the identification of potential prognostic and diagnostic markers [[33], [34], [35], [36], [37], [38]]. Pioneering work utilizing proteomics of human ITA have demonstrated frequent changes of extracellular matrix proteins in response to pathological stimuli such as abdominal aortic aneurysms, type 2 diabetes, and high pulse wave velocity [[35], [36], [37]]. Similarly, proteomics of human SV has identified changes in smooth muscle contractile proteins and extracellular matrix proteins in response to hemodynamic stress [33,34,37]. However, no studies have yet comprehensively compared the proteomic profiles of ITA and SV grafts. Therefore, we aimed to address this gap by investigating the biological differences between human arteries and veins at the proteomic level.

Crotonylation proteomics, one of the most up-to-date PTM proteomics, is a powerful tool to identifying global tissue proteins as and quantifying both protein expression and crotonyl-modifications [16]. Over the past decade, global profiling of crotonylation has been determined in various human cancer cells and peripheral blood mononuclear cells, shedding light on the functional associations of lysine crotonylation with cancer [39], kidney disease [40] and pulmonary disease [41]. While a recent study suggests the potential role of Kcro in vascular smooth muscle cell phenotypic remodeling [22], a comprehensive crotonylation profile in human vessels remains unexplored. In this study, we demonstrate that lysine crotonylation exists in both human artery and vein (Fig. 1A & C), highlighting the widespread relevance of Kcro in physiological function of human circulatory system. Moreover, our analysis reveals significant differences in protein crotonylation between ITA and SV, with most differentially modified proteins being non-histone proteins that are involved in cell structure, cell motion, cell differentiation, and energy metabolism (Fig. 2, Fig. 3). These findings demonstrate the original biological divergence of human artery and vein in term of protein composition. The abundance of Kcro on focal adhesion and myofilament proteins, previously reported in other cells and species [26,40], indicates that Kcro plays conserved functional roles in cell motility and muscle contraction. Furthermore, KEGG pathway enrichment analysis reveals distinct carbohydrate metabolism patterns in ITA and SV, particularly in glycolysis enzymes such as GAPDH, PGK1, ENO1 and PKM (Fig. 3A *right panel*). Notably, some Kcro sites (e.g. GAPDH-K194) can also be modified by other acyl-groups, including acetyl-, malonyl-, or dimethyl-group (https://www.uniprot.org/uniprot/P04406), while some sites (e.g. PKM-K135) were identified for the first time as being modified by the crotonyl group. From the perspective of vascular pathology, these results give us a hint that further research should focus on the energy metabolism pathways in both vessels to identify potential target proteins correlating to atherosclerotic risk and graft patency.

Non-histone crotonylation can regulate activity, localization and degradation of specific proteins [39,42,43]. However, it remains unclear regarding whether and how crotonylation influences proteins correlated with vascular function. To investigate this, we focus on 10 proteins for further study. From an initial analysis of 131 differentially-crotonylated proteins (see Supplemental DataSet 2), we distinguish those most prominently involved in key vascular regulatory pathways, including focal adhesion, anti-oxidation, glycolysis, endothelial NO⋅ production, neointima formation, and smooth muscle cell dedifferentiation. Among them, we select three functional-measurable candidates (TXN, GLO1 and GAPDH) for validation in Hek293 cell. As shown in Fig. 5, Fig. 6, Fig. 7, Fig. 8, the results demonstrate that both whole-cell and site-specific crotonylation (TXN-K3, GLO1-K157, and GAPDH-K61) decrease the enzymatic activities of the three proteins, suggesting a broadly negative-regulatory role of non-histone crotonylation on protein activity.

Previous studies have shown that the long-term patency rate of CABG grafts is closely related to plasma oxidative stress levels. Supplement of antioxidants has been shown to improve long-term survival rates after CABG surgery [[44], [45], [46]], likely through mechanisms such as reduced inflammation, increased NO⋅ levels, and controlled production of atherosclerotic factors. An investigation by Shi and the colleagues revealed that veins exhibit poorer antioxidant properties compared to arteries [9]. In the present study, we find that site-specific crotonylation of TXN, GLO1, and GAPDH leads to a reduction in their activity, which, in turn, may contribute to the higher oxidative stress observed in SV. Among the three proteins, TXN is well-known for its antioxidant function in vascular endothelial and smooth muscle cells, primarily by decreasing the NADPH oxidase activity and the production of reactive oxygen species [47]. GLO1, the major enzyme detoxifying methylglyoxal (MGO) and the subsequent advanced glycation end products of AGEs, protects vessels from atherosclerosis by inhibiting endothelial dysfunction and smooth muscle proliferation [48]. The inactivity of GAPDH, the key enzyme in glycolytic process that helps prevent cell death of endothelium and smooth muscle cells [49], is closely related to the accumulation of early glycolytic intermediates and subsequent pathological pathways. Therefore, by establishing a link between crotonylation and increased oxidative stress in blood vessels, this study highlights potential new pathways and biomarkers at PTM level, offering insights into the mechanisms underlying graft failure in CABG. Additionally, it suggests future research directions and clinical strategies, such as long-term antioxidant supplementation following CABG, to improve graft patency.

In the present study, we also investigated the regulatory mechanisms of crotonylation in cellular models and vessel tissues. The results demonstrate that TXN, GLO1 and GAPDH could be crotonylated by CBP and decrotonylated by HDAC1 and 3 (Fig. 8). The SV has a higher level of CBP, which likely contributes to elevated crotonylation of Kcro-TXN, Kcro-GLO1 and Kcro-GAPDH (Fig. 8M–P). Interestingly, inhibition of CBP enhances the activities of TXN, GLO1 and GAPDH by decreasing their crotonylation in the SV graft (Fig. 8J–L), thereby increasing the graft's resistance against oxidative stress. These findings reveal the importance of CBP-related crotonylation in the functional differences between ITA and SV, suggesting that CBP could serve as a promising target for the regulating graft patency. Developing small molecules that specifically inhibit CBP's crotonylation activity may provide a strategy to reduce the crotonylation level in the SV grafts. However, given that CBP is involved in a wide range of acylation processes, any potential inhibitors would need to be selective to avoid unwanted broad transcriptional repression, which could lead to adverse effects [50].

As a new direction to identify the biological differences between the arterial and venous grafts that may reveal the biological basis of the different long-term behavior of these two kinds of grafts, this study suggests that PTM of proteins of the vessels may play an important role. Therefore, targeting on the PTM may form new therapeutic strategy as to further improve the long-term results of CABG.

**Limitations.** By investigating the differences of crotonylation between ITA and SV grafts, the present study clearly demonstrates that PTM of proteins plays an important role in regulating patency-related proteins in CABG grafts. The limitations of the present study include the relatively small sample size in the PTM proteomics analysis, which is a common challenge in proteomics studies, although a new cohort of samples was further investigated in the validation experiments. Further, potential biases in the PTM proteomics analysis should be considered. Future studies should also consider the need for *in vivo* validation to fully understand the role of PTMs.

## 5 Conclusions

In conclusion, this study is the first to perform a comprehensive analysis of crotonylome and Kcro modifications in human vessels. We demonstrate that there are significant differences in PTM of proteins between ITA and SV grafts. These differences, particularly in proteins regulated by CBP, influence cellular functions such as oxidative stress response and other key processes. The variations in crotonylation modifications between ITA and SV contribute to the biological basis underlying their differing long-term patency outcomes. Our findings highlight the importance of protein PTMs in the grafting vessels and form a scientific basis for developing specific methods including new anti-oxidative drugs and gene therapy to target on crotonylation in the vein graft in order to improve the long-term graft patency.

## CRediT authorship contribution statement

**Wen-Tao Sun:** Writing – original draft, Validation, Methodology, Investigation, Funding acquisition, Formal analysis. **Huan-Xin Chen:** Investigation, Data curation. **Hai-Tao Hou:** Investigation, Data curation. **Hong-Mei Xue:** Formal analysis. **Qin Yang:** Formal analysis. **Guo-Wei He:** Writing – review & editing, Visualization, Supervision, Resources, Project administration, Methodology, Investigation, Funding acquisition, Formal analysis, Conceptualization.

## Availability of data and materials

The mass spectrometry proteomics data have been deposited to the ProteomeXchange Consortium (http://proteomecentral.proteomexchange.org) via the iProX partner repository with the dataset identifier PXD026404.

## Ethics approval and consent to participate

The study fully conformed to the principles of the Declaration of Helsinki. The protocol including collection of the redundant ITA and SV graft samples was approved by the Institutional Review Board (IRB) of TEDA International Cardiovascular Hospital (No. [2019]-0516-1).

## Declaration of generative AI and AI-assisted technologies in the writing process

No generative AI and AI-assisted technologies were used in the writing process.

## Sources of funding

This work was supported by the 10.13039/501100001809National Natural Science Foundation of China [82170353 & 82370350 & 82200440]; the Non-profit Central Research Institute Fund of Chinese Academy of Medical Sciences [2019XK310001 & 2020-PT310-007]; the 10.13039/501100007129Shandong Provincial Natural Science Foundation [ZR2022QH234], Taishan Scholar Young Talent Program [tsqn202312241]; Tianjin Municipal Heath Commission [TJWJ2023MS054], and Special Fund for High Quality Development Project.

## Declaration of competing interest

None.

## Data Availability

The mass spectrometry proteomics data have been deposited to the ProteomeXchange Consortium (http://proteomecentral.proteomexchange.org) via the iProX partner repository: dataset identifier PXD026404.
