# Supplementary material for: Protein post-translational modification crotonylation of TXN and GLO1 in artery and vein grafts for coronary artery surgery
Source: Redox Biol. 2025 Mar 22;82:103608. doi: 10.1016/j.redox.2025.103608 (PMC11986619; doi:10.1016/j.redox.2025.103608)
Supplement: Multimedia component 1 [file mmc1.docx]

**For resubmission to *Redox Biology* February 27, 2025**

**SUPPLEMENTARY MATERIAL. for Article Entitled:**

**Protein Post-translational Modification Crotonylation of TXN and GLO1 in Artery and Vein Grafts for Coronary Artery Surgery**

Wen-Tao Sun^1,3^, Huan-Xin Chen^1^, Hai-Tao Hou^1^, Hong-Mei Xue^1^, Qin Yang^1^, and Guo-Wei He^1,2,4*^

^*^**Correspondence author:**

**Professor Guo-Wei He, *MD, PhD, DSc***

**Distinguished Professor of Tianjin University**

**Foreign Correspondence Member,**

**The National Academy of Medicine, France.**

TEDA International Cardiovascular Hospital, No.61, the 3rd Ave, TEDA, Tianjin, 300457 &

Department of Surgery, Oregon Health and Science University, Portland, Oregon, U.S.A.

Tel 86-22-6520 9089; Fax 86-22-6520 8732

E-mail: [gwhezj@163.com](mailto:gwhezj@163.com) OR gwhe@tju.edu.cn

**This file includes** **Supporting Information**

**Supplementary Methods**

**Table S1.** Demographic and clinical characteristics of patients whose paired ITA and SV segments were collected.

**Table S2.** Concentration of the six protein extracts of ITA and SV for crotonyl-proteomics.

**Table S3.** Differentially expressed crotonylated proteins in ITA/SV we chose for validation.

**Fig. S1.** Flowchart of the sample collection and the subsequent Kcro proteomics analysis.

**Fig. S2.** The motif enrichment feature of crotonylated site.

**Fig. S3.** The accuracy analysis of the MS data from crotonyl-proteomics.

**Fig. S4.** The statistical analysis of the repeatability of quantification in three independent groups of ITA/SV samples.

**Fig. S5.** GO-based enrichment analysis of crotonylated proteins.

**Fig. S6.** Functional annotation and enrichment of differentially-crotonylated proteins in human ITA and SV grafts.

**Fig. S7.** Hierarchical clustering analysis the differentially crotonylated sites in ITA and SV.

**Fig. S8.** Validation of gene over-expression cellular model.

**References**

**Supplemental Methods**

**Availability of human ITA and SV segments**

In the CABG surgery, the artery and vein were usually taken with slightly longer length for the safety and effectiveness of the operation. After the artery or vein is removed from the body, the exact length of the graft required was accurately measured and the small segment of redundant vessel was collected for this experiment. when both ITA and SV had redundant segments from the same patient available for experiments (≥50mg/sample), these samples were allocated to this study.

**Western blot for global crotonylation assay**

Protein extracts of ITA/SV segments were fractionated by sodium dodecyl sulfate polyacrylamide gel electrophoresis (12% SDS-PAGE, 40 μg/lane), followed by electrotransferred to a polyvinylidene difluoride (PVDF) membrane (Thermo Scientific, USA) for coomassie blue staining and detecting the modifications of crotonylation.

**Trypsin digestion**

The protein solution was reduced with 5 mM dithiothreitol for 30 min at 56 °C and alkylated with 11 mM iodoacetamide for 15 min at room temperature in darkness. The protein sample was then diluted by adding 100 mM TEAB to urea concentration less than 2 M. Finally, trypsin was added at 1:50 trypsin-to-protein mass ratio for the first digestion overnight and 1:100 trypsin-to-protein mass ratio for a second 4h-digestion.

**TMT labeling**

After trypsin digestion, peptide was desalted by Strata X C18 SPE column (Phenomenex) and vacuum-dried. Peptide was reconstituted in 0.5 M TEAB and processed according to the manufacturer’s protocol for TMT kit. Briefly, one unit of TMT reagent were thawed and reconstituted in acetonitrile. The peptide mixtures were then incubated for 2 h at room temperature and pooled, desalted and dried by vacuum centrifugation.

**Affinity enrichment of crotonylated peptides**

To enrich lysine crotonylated peptides, tryptic peptides dissolved in NETN buffer (100 mM NaCl, 1 mM EDTA, 50 mM Tris-HCl, 0.5% NP-40, pH 8.0) were incubated with pre-washed antibody beads (PTM503, PTM Bio) at 4 °C overnight with gentle shaking. The beads were then washed four times with NETN buffer and twice with H2O. The bound peptides were eluted from the beads with 0.1% trifluoroacetic acid. Finally, the eluted fractions were combined and vacuum-dried. For LC-MS/MS analysis, the resulting peptides were desalted with C18 ZipTips (Millipore) according to the manufacturer’s instructions. The selectivity/specificity of the antibody was evidenced by the dot-blot from Jingjie PTM Biolab Co. Ltd, Hangzhou, China^1^.

**LC-MS/MS analysis**

The enriched crotonylated peptides were dissolved in 0.1% formic acid (solvent A), directly loaded onto a reversed-phase analytical column. The gradient was comprised of an increase from 6% to 23% solvent B (0.1% formic acid in 98% acetonitrile) over 26 min, 23% to 35% in 8 min and climbing to 80% in 3 min then holding at 80% for the last 3 min, all at a constant flow rate of 400 nL/min on an EASY-nLC 1000 UPLC system. The peptides were subjected to NSI source followed by tandem mass spectrometry (MS/MS) in Q ExactiveTM Plus (Thermo Scientific, USA) coupled online to the UPLC. The applied electrospray voltage was 2.0 kV, and both the peptide precursor and its secondary fragments were detected and analyzed using high-resolution Orbitrap. The scan range of the primary mass spectrometry was set to 350-1600 m/z, and the scan resolution was set to 120,000. The scan range of the secondary mass spectrometry was fixed at 100 m/z, and the scan resolution of Orbitrap was set to 15,000. A data-dependent scanning (DDA) program was selected as the data acquisition mode. After the primary scan, the first 20 peptide precursor ions with the highest signal intensity were selected to enter the HCD collision cell sequentially and then triggered the fragmentation by using 28% of the fragmentation energy, and sequentially performed the secondary mass spectrometry analysis. In order to improve the effective utilization of the mass spectrometry, the automatic gain control (AGC) was set to 1E5, the signal threshold was set to 50000 ions/s, the maximum injection time was set to 100 ms, and the dynamic exclusion time of the tandem MS scan was set to 10 s.

The accuracy of the MS data was validated by analyzing the mass error and distribution of the identified peptides (eFigure 3).

**Identification of up- or down- crotonylated proteins in ITA and SV**

The average values of each sample (ITA1, SV1, ITA2, SV2, ITA3 and SV3) were obtained from three repeated experiments. The ratios of the average values between the ITA and SV groups (ITA1 vs. SV1, ITA2 vs. SV2, ITA3 vs. SV3) were calculated. Further, the log2 of each value was taken for the p-value calculation from two-tailed T-test. When p-value <0.05, the ratio of ITA/SV was applied as a threshold. Details of the protein information can be found in Data Set S1. When the ratio was higher than 1.3 or lower than 1/1.3, it was identified as significant up- or down- regulation (Data Set S2). It is worth noting that all the Kcro-proteomics data reported in this study have been normalized by the general proteomics results of the same samples. The details of protein quantification were explained below.

**TMT-labeled Quantitative Proteomics** The raw LC-MS datasets were first searched against database and converted into matrices containing reporter intensity of peptides across samples. The relative quantitative value of each protein was then calculated based on these intensity values by the following steps:

1. Firstly, the intensities of peptide (I) across all samples were centralized and transformed into their values of relative quantification (U) in each sample. The formula is as follows: i denotes the sample and j denotes the peptide.

Uij = Iij / Mean(Ij)

1. To adjust the systematic bias of the identified peptide amount among different samples in the process of mass spectrometry detection, the relative quantitative value of peptide needs to be corrected by median value as follows:

NRij = Uij / Median(Ui)

1. The relative quantitative value of a protein (R) is calculated by the intensity median of its corresponding unique peptides. The formula is listed as follow where k denotes the protein and j denotes the unique peptides belonging to the protein:

Rik = Median(NRij, j∈k)

**TMT-labeled Quantitative PTM Proteomics** The raw LC-MS datasets were first searched against database and converted into matrices containing reporter intensity of peptides across samples. The relative quantitative value of each modified peptide was then calculated based on these intensity information by the following steps:

1. Firstly, the intensities of modified peptides (I) were centralized and transformed into relative quantitative values (U) of modified peptides in each sample. The formula is listed as follow: i denotes the sample and j denotes the modified peptide.

Rij = Iij / Mean(Ij)

1. If both Proteomics and Post-translational modification profiling were conducted ed on the same cohort, the relative quantitative value of the modified peptide is usually divided by the relative quantitative value of corresponding protein to remove the influence from protein expression of modifications.

**Repeatability tests**

For biological replicates or technical replicates, we test whether the quantitative results are statistically consistent. Here we used three statistical analysis methods, principal component analysis (PCA), relative standard deviation (RSD) and Pearson's correlation coefficient, to evaluate the repeatability of Kcro quantification. eFigure 4a is the PCA graph showing the Kcro quantification results of all samples. Higher aggregation between repeated samples means better quantitative repeatability. eFigure 4b is an RSD boxplot of the Kcro quantification values between replicate samples. The smaller the overall RSD value, the better the quantitative repeatability. eFigure 4c is a heatmap of Pearson’s correlation coefficients from all quantified proteins between each pair of samples. This coefficient is a value that measures the degree of linear correlation between two sets of data. When it is close to -1, the data is negatively correlated; when it is close to 1, the data is positively correlated; and when it is close to 0, the data is irrelevant.

**Antibodies used in the study**

The following primary antibodies were used in the experiments: Pan Kcro (PTM Biolabs 501\502), beta Actin (Absinabs137975), beta Tubulin (Cell Signaling Technology 2128), Thioredoxin-1 (Abcam ab16965), ALDH2 (Abcam ab108306), Talin-1 (Abcam ab157808), Tropomyosin-1 (Abcam ab133292), Tropomyosin-2 (Abcam ab180176), Tropomyosin-3 (Abcam ab113692), Tropomyosin-4 (Abcam ab181085), LAMC1 (Abcam ab233389), GAPDH (Cell Signaling Technology 5174) and GLO1 (Abcam ab226353). Secondary antibodies including HRP-linked goat anti-rabbit IgG (CST 7074) and horse anti-mouse IgG (CST 7076) were applied in the blotting step.

**Validation of over-expressing cell model (Figure S5.)**

Total RNA was extracted from HEK293cells using TRIzol reagent (Thermo Scientific, USA) and reverse transcription and Q-PCR amplification were performed in LightCycler 96 (Roche, Basel, Switzerland) employing TransScript Green Two-Step qRT-PCR SuperMix system (Transgen, Beijing, China) under optimal PCR cycle conditions: 94 °C for 30 seconds, 45 cycles of 5 seconds at 94 °C, 50 °C for 15 seconds, 72 °C for 10 seconds, and melting at 95 °C for 10 seconds, 65 °C for 10 seconds and 97 °C for 1 second. The following primers were used for amplification: Glyceraldehyde-3-phosphate dehydrogenase (GAPDH), 5’-CATCCCTGCCTCTACTG-3’ (forward) and 5’-GCTTCACCACCTTCTTG-3’ (reverse); Thioredoxin (TXN), 5’-GCCTTTCTTTCATTCCCTCTC-3’ (forward) and 5’-CACACTCTGAAGCAACATCC-3’ (reverse); Lactoylglutathione lyase(GLO1), 5’-AGACCCAGAGTTACCACAATG-3’(forward) and 5’-TTCAATCCAGTAGCCATCAGG-3’ (reverse); CREB-binding protein (CBP), 5’-ATGAAGCAGCAGATTGG-3’ (forward), and 5’-CGCACCTGGTTACTAAG-3’ (reverse); Histone deacetylase 1 (HDAC1), 5’-GCTCCACATCAGTCCTTCC-3’ (forward) and 5’-TCCTCATCGCCACTCTCC-3’ (reverse); Histone deacetylase 3 (HDAC3), 5’-TGATGACCAGAGTTACAAG-3’ (forward) and 5’-CCAGCACGAGTAGAGG-3’ (reverse). Beta-actin (ACTB) was amplified in parallel as an internal loading control with the primers 5’-GCTGTGCTATGTTGCC-3’ (forward) and 5’-GTTGCCGATGGTGATG-3’ (reverse). Qualitative PCR was performed in triplicate foreach gene. The threshold cycle (Ct) values were calculated and statistically evaluated by SPSS version 20 (IBM-SPSS Inc, Armonk, NY). Expression of the target messenger RNAs was normalized to beta-actin levels and relative differences were determined using the comparative Ct (∆∆Ct) method, and fold expression was calculated as 2–∆∆Ct, where ∆∆Ct represents ∆Ct values normalized with the mean ∆Ct of control samples.

Whole-cell proteins of HEK293 cells were extracted and the proteins of interest were detected using specific primary antibodies including GAPDH, TXN, GLO1, CBP (Abcam ab2832), HDAC1 (CST 5356) and HDAC3 (CST 3949).

Immunoprecipitation was also applied for verifying the Kcro levels of TXN, GLO1 and GAPDH in HEK293 cells. Primary antibodies including TXN, GLO1, GAPDH, and FLAG (Abcam ab1162) were used in the experiments. The procedures of protein extraction, concentration determination and protein pull-down were the same as described in the previous methods.

**Simulation of binding of substrate and enzymes**

Auto-Dock 4.0 was used to dock small molecules (substrates) into their enzyme structures with or without Kcro modification, respectively. The original enzyme structure was achieved from the PDB database. The enzymatic structure with Kcro was generated by modifying the side chain of lysine using Pymol software. The structure of small molecules was downloaded from Chemspider. In this study, the bindings of three systems including TXN - NADP (+), GLO1 - (R)-S-lactoylglutathione, and GAPDH – NAD (+) were simulated. The information on active sites and substrate binding sites of TXN, GLO1 and GAPDH were predicted by COACH. Before the docking simulation, the small molecule was placed into the substrate binding site of enzyme as the start point of docking. The parameters for docking were set as follows: the Lamarckian genetic algorithm (LGA) runs were set at 100, and the maximum number of energy evaluations was set at 25 million. The simulation box was fixed at the center of the substrate and the box size was set at 126 Å×126 Å×126 Å in all three dimensions. The conformation with the lowest binding energy of small molecule was considered as the enzyme-bound conformation. By comparing the binding energy and conformation of the enzymes with or without Kcro modification, we infer the likely effect of the modification on the enzymatic activity.

**Table S1. Demographic and clinical characteristics of patients whose paired ITA and SV segments were collected**

| **Characteristics** | **Patients undergoing CABG (N=75)** | **Patients underlying CABG whose vessels were collected for proteomics (N=12)** |
| --- | --- | --- |
| Gender (n, %) |  |  |
| Males | 56 (74.7) | 12 (100) |
| Females | 19 (25.3) | 0 (0) |
| Ages |  |  |
| Range | 46-79 | 54-76 |
| Mean±SD | 66.4±7.4 | 64.4±6.5 |
| Diagnosis (n, %) |  |  |
| Unstable angina | 52 (69.3) | 7 (58.3) |
| Acute NSTEMI | 13 (17.3) | 3 (25) |
| Stable angina | 5 (6.7) | 2 (16.7) |
| Acute anterior myocardial infarction | 3 (4) | 0 (0) |
| Acute STEMI | 1 (1.3) | 0 (0) |
| Acute inferior myocardial infarction | 1 (1.3) | 0 (0) |
| Clinical pathways (n, %) |  |  |
| CAG & CABG | 75 (100) | 12 (100) |

NSTEMI: non-ST-segment elevation myocardial infarction; STEMI: ST-segment elevation myocardial infarction; CAG: coronary angiography; CABG: coronary artery bypass surgery.

**Table S2. Concentration of the six protein extracts of ITA and SV for crotonyl-proteomics**

| **Number** | **Protein concentration（μg/μL）** | **Sample volume（μL）** | **Total protein（μg）** |
| --- | --- | --- | --- |
| ITA-1 | 4.6 | 1000 | 4569 |
| SV-1 | 6.1 | 1000 | 6132 |
| ITA-2 | 4.9 | 1000 | 4871 |
| SV-2 | 3.9 | 1000 | 3884 |
| ITA-3 | 3.4 | 1000 | 3373 |
| SV-3 | 5.3 | 1000 | 5259 |

**Table S3. Differentially expressed crotonylated proteins in ITA/SV we chose for validation**

| Protein accession | Gene name | Modified sequence | ITA/SV Ratio (Expression) | ITA/SV Ratio (Crotonylation) | ITA/SV  P value | Position | Protein-related Pathway/Function |
| --- | --- | --- | --- | --- | --- | --- | --- |
| P11047 | LAMC1 | K(1)VSDLENEAK | 0.634 | 0.733 | 0.026442 | 1557K | Focal adhesion^2^ |
| Q9Y490 | TLN1 | K(1)LEQLKPR | 0.781 | 0.734 | 0.023783 | 2322K | Focal adhesion^3^ |
|  |  | AGALQCSPSDAYTK(1)K |  | 0.691 | 0.0077026 | 1947K |  |
|  |  | AK(1)SVAQR |  | 0.741 | 0.00165536 | 687K |  |
|  |  | K(0.242)NK(0.758)MDESK |  | 0.721 | 0.041121 | 535K |  |
|  |  | VK(1)ADQDSEAMK |  | 0.52 | 0.0100228 | 2445K |  |
|  |  | K(1)LLSAAK |  | 0.701 | 0.028757 | 855K |  |
| P09493 | TPM1 | EAK(1)HIAEDADR | N/A | 0.701 | 0.028805 | 152K | Focal adhesion^4^ |
|  |  | AQK(1)DEEK |  | 0.764 | 0.014757 | 136K |  |
| P07951 | TPM2 | LEQAEK(1)K | N/A | 0.441 | 0.037123 | 76K | Focal adhesion^5^ |
|  |  | EDK(1)YEEEIK |  | 0.441 | 0.02742 | 220K |  |
|  |  | EDKYEEEIK(1)LLEEK |  | 0.479 | 0.041176 | 226K |  |
|  |  | YSESVK(1)EAQEK |  | 0.468 | 0.023616 | 65K |  |
| P06753 | TPM3 | EDK(1)YEEEIK | 1.267 | 0.594 | 0.0044197 | 221K | Focal adhesion^6^ |
|  |  | MELQEIQLK(1)EAK |  | 0.711 | 0.025417 | 150K |  |
| P67936 | TPM4 | LK(1)EAETR | N/A | 0.576 | 1.93051E-05 | 197K | Focal adhesion^7^ |
|  |  | EDK(1)YEEEIK |  | 0.689 | 0.033457 | 184K |  |
|  |  | AMK(1)DEEK |  | 0.608 | 0.0021995 | 100K |  |
| P10599 | TXN | VK(1)QIESK | N/A | 0.639 | 0.0059227 | 3K | Anti-oxidant^8^ |
| Q04760 | GLO1 | K(0.001)PDDGK(0.999)MK | N/A | 0.621 | 0.0105383 | 157K | Anti-oxidant^9^ |
| P04406 | GAPDH | TVDGPSGK(1)LWR | N/A | 0.757 | 0.0131805 | 194K | Anti-oxidant & Glycolysis^10,11^ |
|  |  | FHGTVK(1)AENGK |  | 0.706 | 0.0013016 | 61K |  |
| P05091 | ALDH2 | TEQGPQVDETQFK(1)K | N/A | 0.704 | 0.00173767 | 368K | Glycolysis^12^ |

LAMC1: Laminin subunit gamma-1; TLN1: Talin-1; TPM1: Tropomyosin alpha-1 chain; TPM2: Tropomyosin beta chain; TPM3: Tropomyosin alpha-3 chain; TPM4: Tropomyosin alpha-4 chain; TXN: Thioredoxin; GLO1: Lactoylglutathione lyase; GAPDH: Glyceraldehyde-3-phosphate dehydrogenase; ALDH2: Aldehyde dehydrogenase, mitochondrial. N/A means no significant difference was detected between ITA/SV by the LC-MS/MS. K is the abbreviation of lysine.


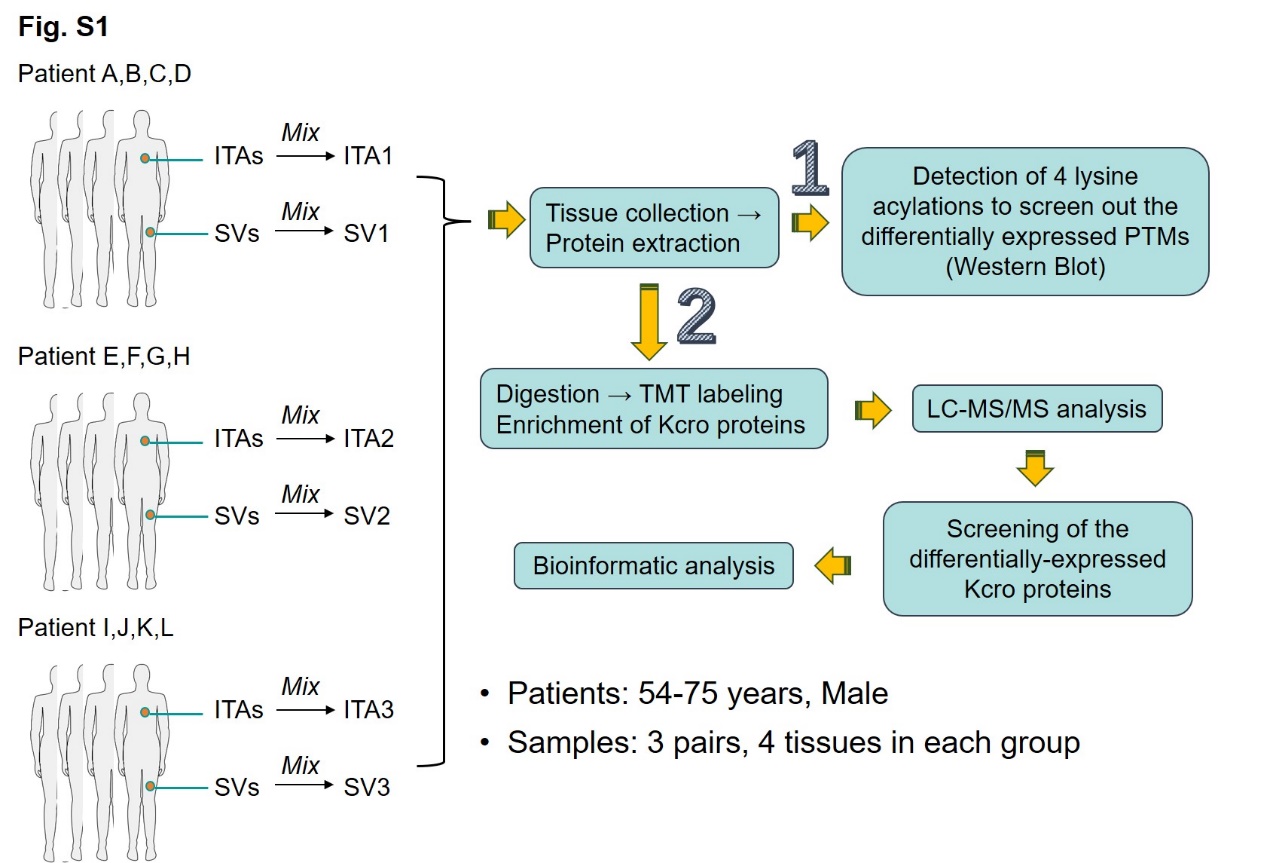


**Fig. S1. Flowchart of the sample collection and the subsequent Kcro proteomics analysis.**


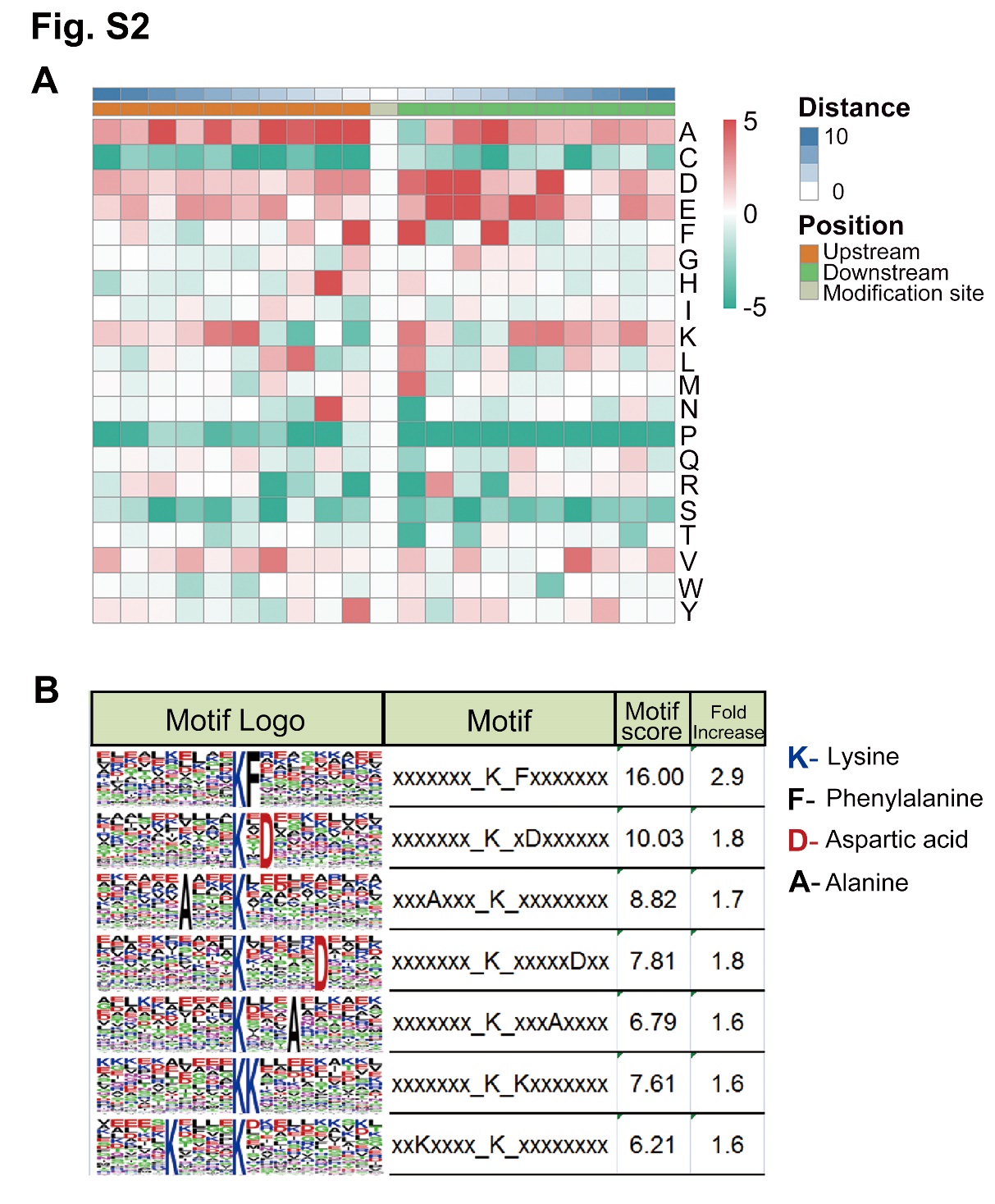


**Fig. S2. The motif enrichment feature of crotonylated site.** (A) The motif enrichment heatmap of upstream and downstream amino acids of all identified modification sites. Red indicates that the amino acid is significantly enriched near the modification site, and green indicates that the amino acid is significantly reduced near the modification site. (B) Crotonylated site feature sequence and its enrichment statistics from Modification Motif (MoMo) software.


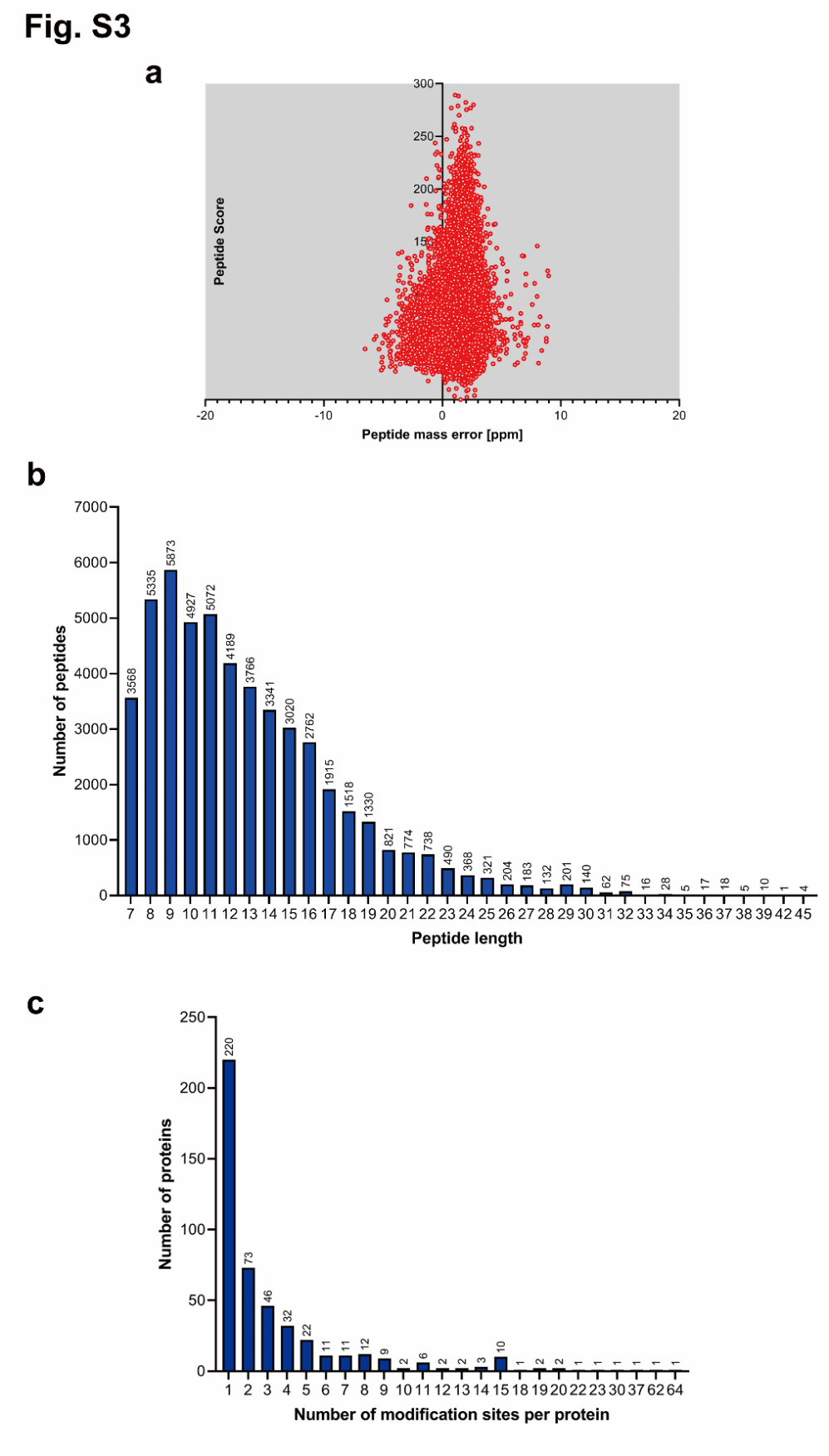


**Fig. S3. The accuracy analysis of the MS data from crotonyl-proteomics.** (a) The distribution of mass errors was near zero and most errors were smaller than 10 ppm, demonstrating the accuracy of the MS data. (b) The length of most of the peptides distributed between 7 and 20, which is consistent with the length of tryptic peptides, demonstrating that sample preparation achieved a reasonable standard. (c) The distribution of crotonylation sites showed that the number of crotonylation sites identified per protein.


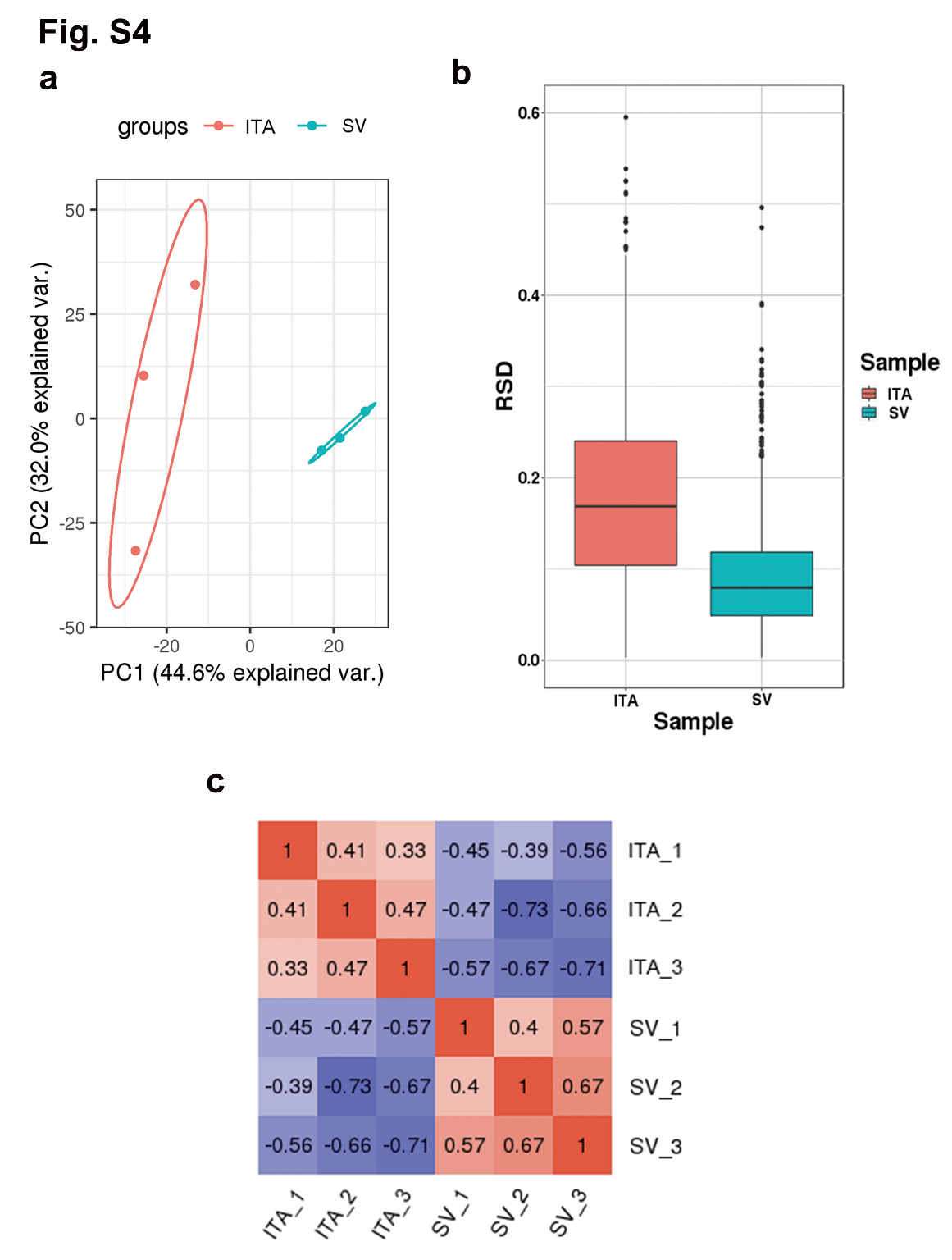


**Fig. S4. The statistical analysis of the repeatability of quantification in three independent groups of ITA and SV samples.** (a) Two-dimensional scatter plots of PCA (principal component analysis) distribution of all samples using quantified proteins. The accumulation of the same type of samples respectively (ITA1, ITA2 and ITA3; SV1, SV2 and SV3) showed a good repeatability of the ITA/SV samples. (b) Box plot of relative standard deviation (RSD) distribution of repeated samples using quantified proteins. (c) Heatmap of Pearson correlation coefficients from all quantified proteins between each pair of samples. It showed that both ITA and SV samples has a positive correlation with the same type of tissues, while they are negatively correlated with the other type of tissue.


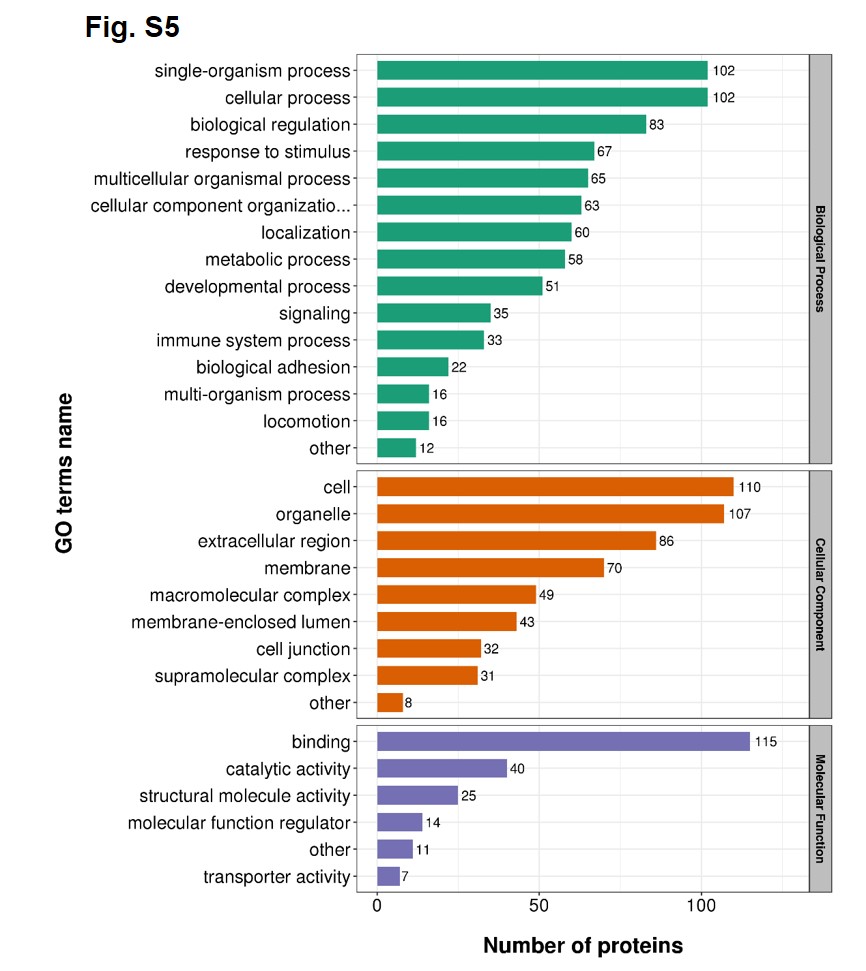


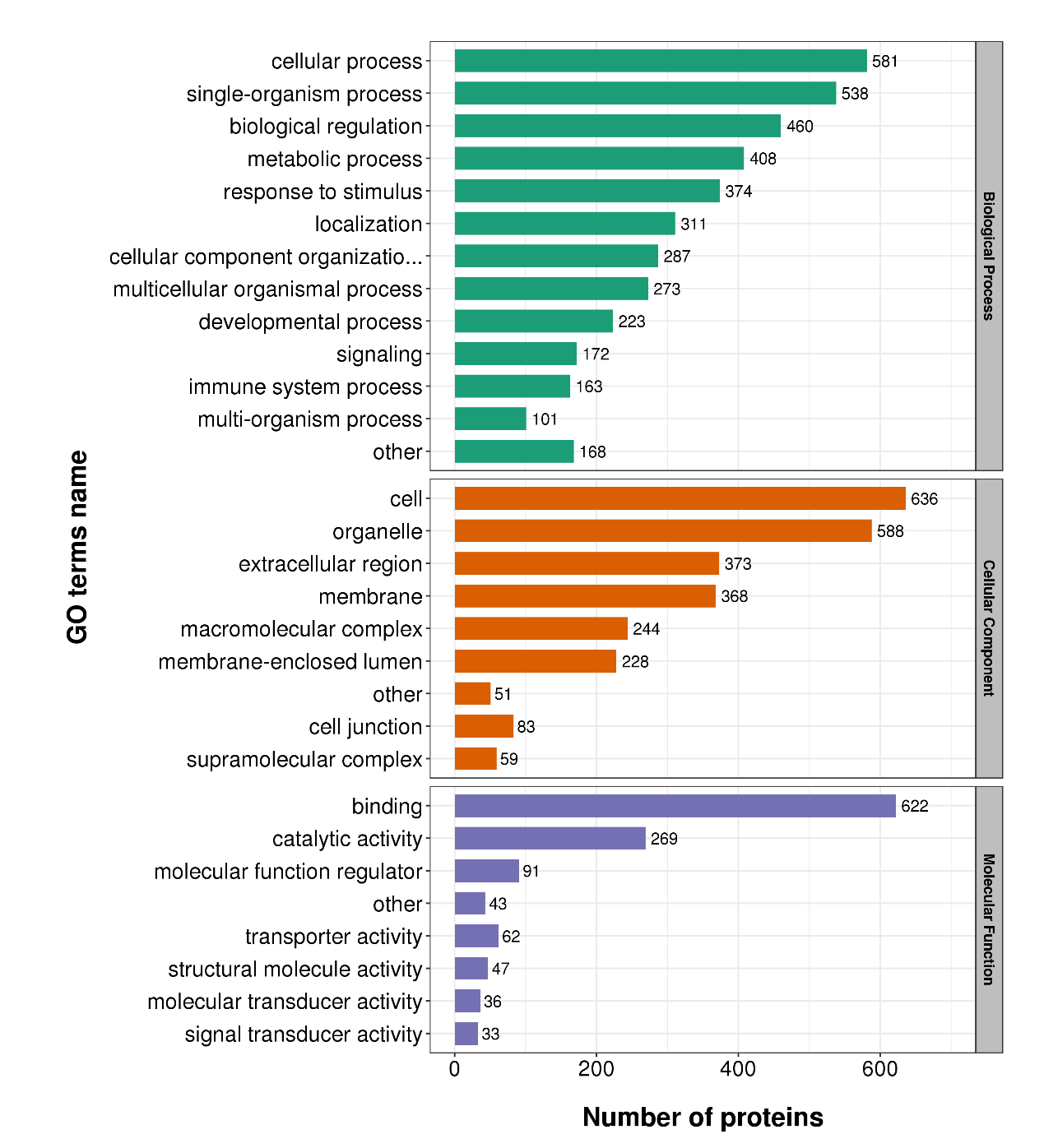


**Fig. S5. GO-based enrichment analysis of differentially-crotonylated (*above*) and differentially-expressed (*below*) proteins in ITA and SV.**


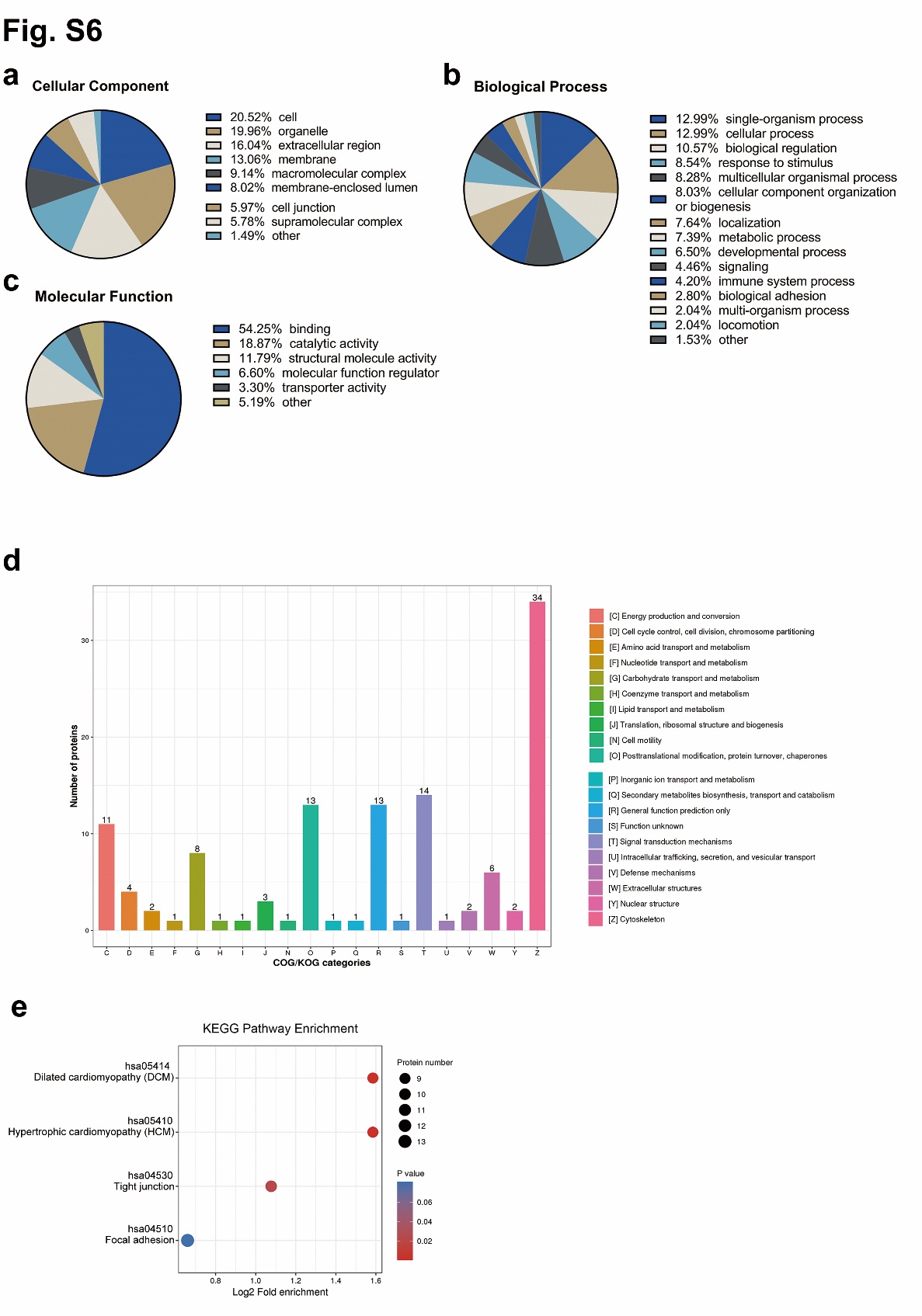


**Fig. S6. Functional annotation and enrichment of differentially-crotonylated proteins in human ITA and SV grafts.** (a)-(c) GO-based enrichment analysis of crotonylated proteins in the format of percentage. (d) COG/KOG functional classification chart of proteins corresponding to differentially expressed modification sites. (e) KEGG pathway enrichment bubble plot of differentially crotonylated proteins.


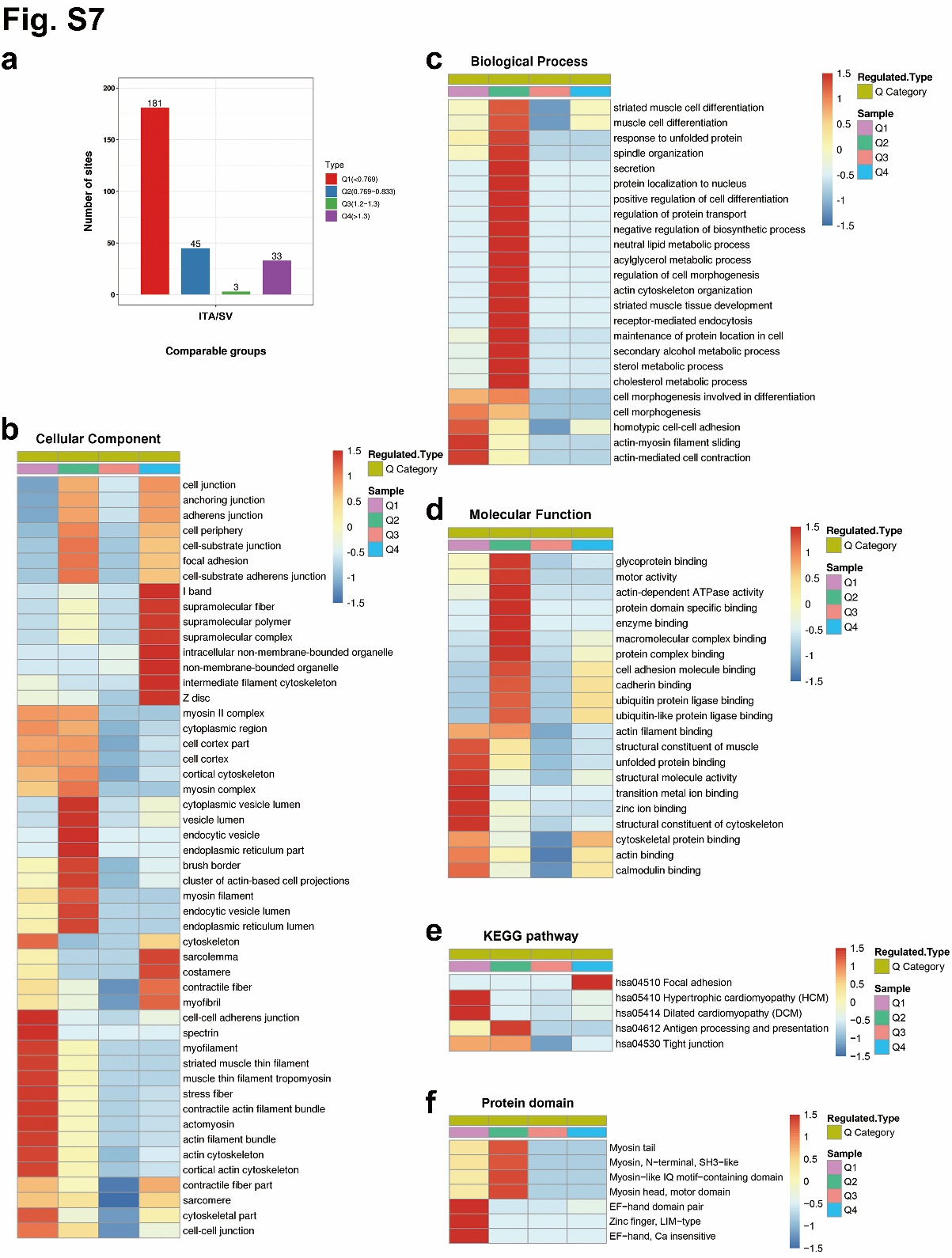


**Fig. S7. Hierarchical clustering analysis the differentially crotonylated sites in ITA and SV.** (a) Differentially expressed modification sites (IMA: SV) are divided into Q1-Q4 distribution according to fold change. Q1 (0< Ratio ≤ 1/1.3), Q2 (1/1.3 < Ratio ≤ 1/1.2), Q3 (1.2 < Ratio ≤ 1.3) and Q4 (Ratio >1.3)). (b)-(f) A comprehensive heatmap for cluster analysis of the enrichment patterns of GO functional categories, KEGG pathways and protein domains. (b, Cellular component, c, Biological process, d, Molecular function, e, KEGG pathway, f, Protein domain).

**Fig. S8**


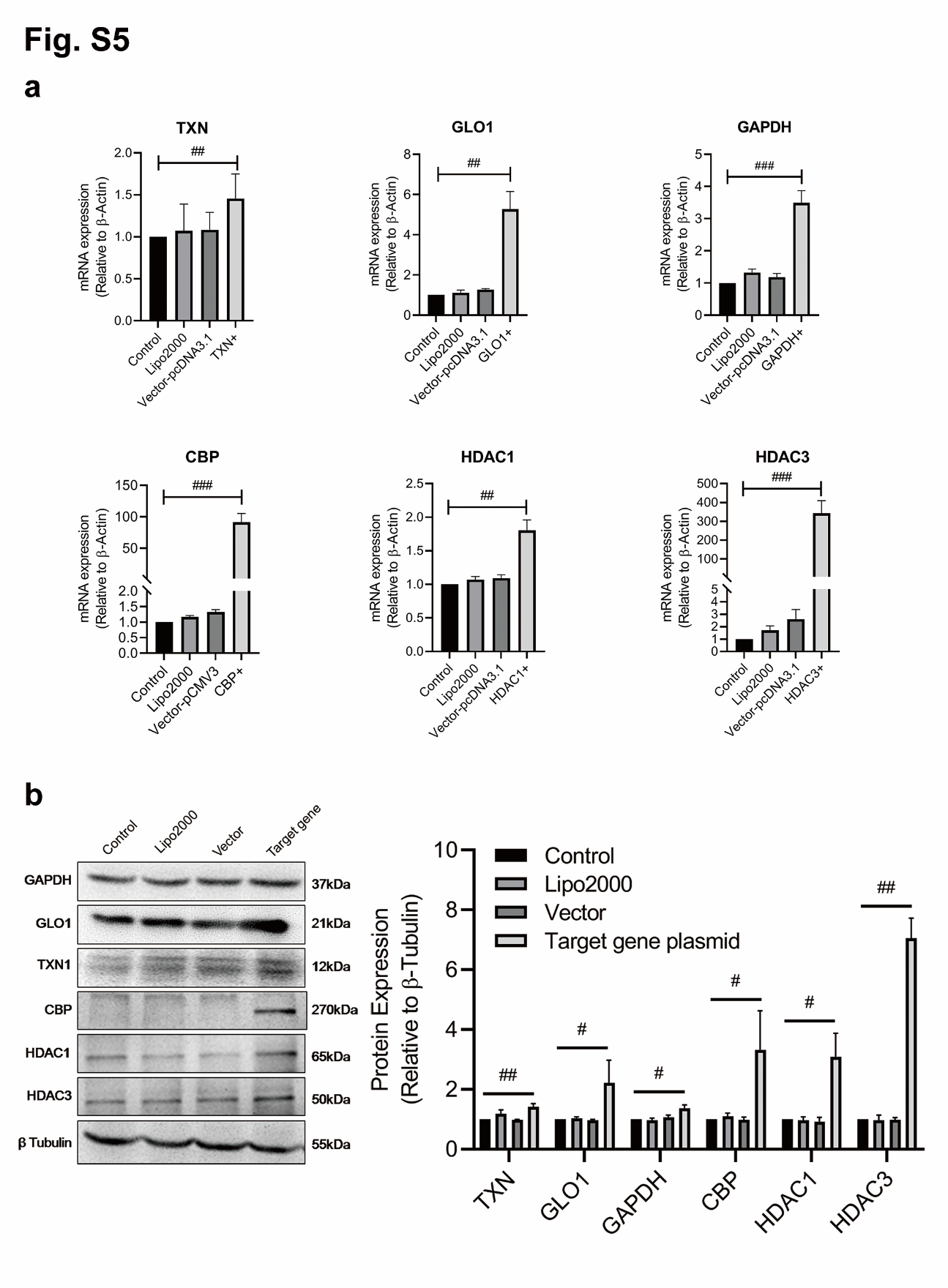


**Figure S8. Validation of gene over-expression cellular model.** (a) Q-PCR results verifying the overexpression of GAPDH, GLO1, TXN, CBP, HDAC1 and HDAC3, respectively. (b) Western blots results verifying the overexpression of the 6 proteins in specific Hek293 cell models.

**References**

1. BIOLABS P. Anti-crotonyllysine antibody conjugated agarose beads. Accessed August 17, 2022. <https://www.ptmbiolabs.com/product/ptm-503/>

2. Hebbel RP, Wei P, Milbauer L, et al. Abnormal Endothelial Gene Expression Associated With Early Coronary Atherosclerosis. *J Am Heart Assoc*. Jul 21 2020;9(14):e016134. doi:10.1161/JAHA.120.016134

3. von Essen M, Rahikainen R, Oksala N, et al. Talin and vinculin are downregulated in atherosclerotic plaque; Tampere Vascular Study. *Atherosclerosis*. Dec 2016;255:43-53. doi:10.1016/j.atherosclerosis.2016.10.031

4. Houle F, Rousseau S, Morrice N, et al. Extracellular signal-regulated kinase mediates phosphorylation of tropomyosin-1 to promote cytoskeleton remodeling in response to oxidative stress: impact on membrane blebbing. *Mol Biol Cell*. Apr 2003;14(4):1418-32. doi:10.1091/mbc.e02-04-0235

5. Meng LB, Shan MJ, Qiu Y, et al. TPM2 as a potential predictive biomarker for atherosclerosis. *Aging (Albany NY)*. Sep 5 2019;11(17):6960-6982. doi:10.18632/aging.102231

6. Abbasian N, Burton JO, Herbert KE, et al. Hyperphosphatemia, Phosphoprotein Phosphatases, and Microparticle Release in Vascular Endothelial Cells. *J Am Soc Nephrol*. Sep 2015;26(9):2152-62. doi:10.1681/ASN.2014070642

7. Abouhamed M, Reichenberg S, Robenek H, Plenz G. Tropomyosin 4 expression is enhanced in dedifferentiating smooth muscle cells in vitro and during atherogenesis. *Eur J Cell Biol*. Sep 2003;82(9):473-82. doi:10.1078/0171-9335-00333

8. Madrigal-Matute J, Fernandez-Garcia CE, Blanco-Colio LM, et al. Thioredoxin-1/peroxiredoxin-1 as sensors of oxidative stress mediated by NADPH oxidase activity in atherosclerosis. *Free Radic Biol Med*. Sep 2015;86:352-61. doi:10.1016/j.freeradbiomed.2015.06.001

9. Hanssen NM, Stehouwer CD, Schalkwijk CG. Methylglyoxal and glyoxalase I in atherosclerosis. *Biochem Soc Trans*. Apr 2014;42(2):443-9. doi:10.1042/BST20140001

10. Hou X, Snarski P, Higashi Y, et al. Nuclear complex of glyceraldehyde-3-phosphate dehydrogenase and DNA repair enzyme apurinic/apyrimidinic endonuclease I protect smooth muscle cells against oxidant-induced cell death. *FASEB J*. Jul 2017;31(7):3179-3192. doi:10.1096/fj.201601082R

11. Shah MS, Brownlee M. Molecular and Cellular Mechanisms of Cardiovascular Disorders in Diabetes. *Circ Res*. May 27 2016;118(11):1808-29. doi:10.1161/CIRCRESAHA.116.306923

12. Zhu H, Wang Z, Dong Z, et al. Aldehyde dehydrogenase 2 deficiency promotes atherosclerotic plaque instability through accelerating mitochondrial ROS-mediated vascular smooth muscle cell senescence. *Biochim Biophys Acta Mol Basis Dis*. Jul 1 2019;1865(7):1782-1792. doi:10.1016/j.bbadis.2018.09.033
