# Supplementary material for: Protein post-translational modification crotonylation of TXN and GLO1 in artery and vein grafts for coronary artery surgery
Source: Redox Biol. 2025 Mar 22;82:103608. doi: 10.1016/j.redox.2025.103608 (PMC11986619; doi:10.1016/j.redox.2025.103608)
Supplement: Multimedia component 2 [file mmc2.pdf]

Fig. 4b LAMC1

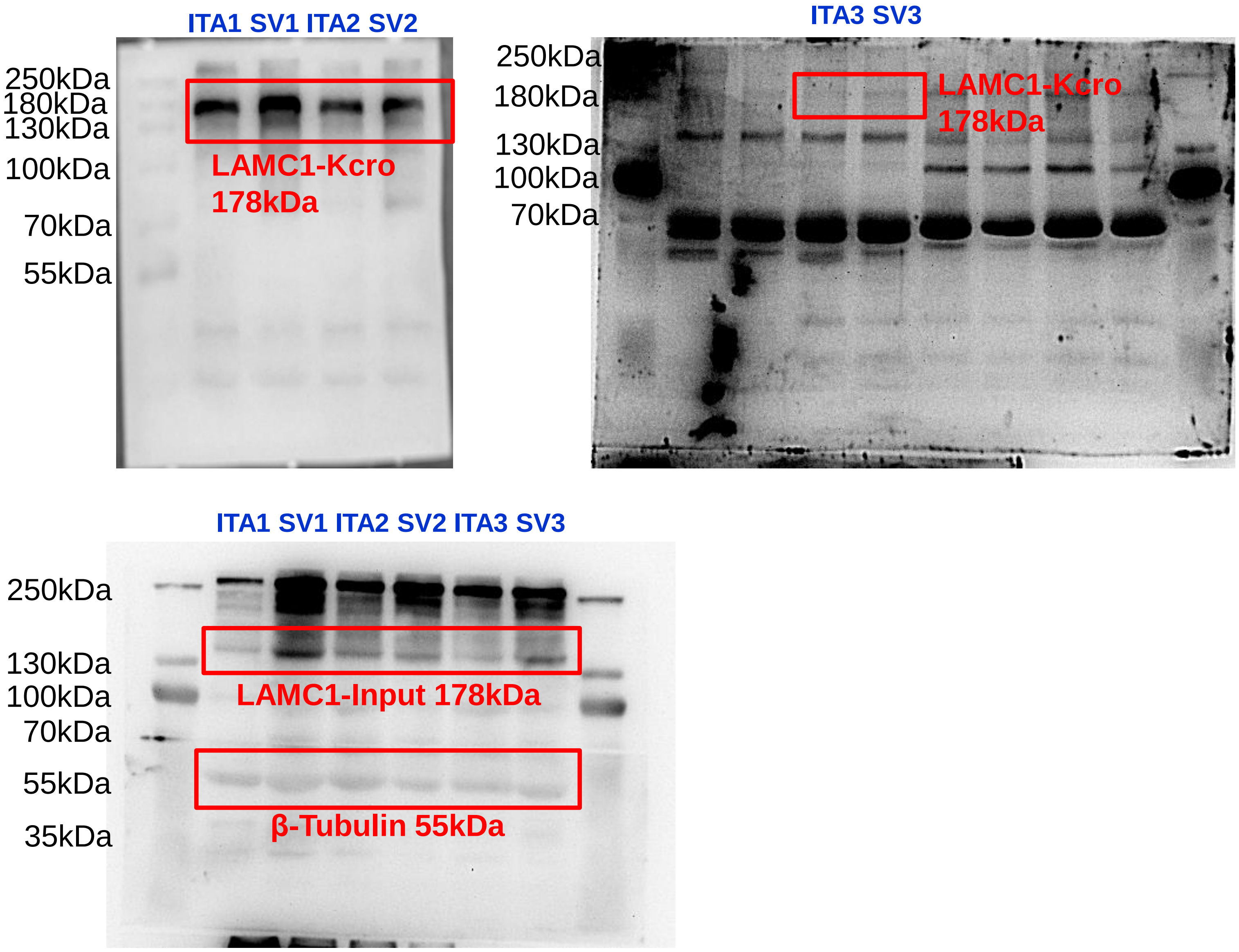
ITA1 SV1 ITA2 SV2
250kDa
180kDa
130kDa
100kDa
70kDa
55kDa
LAMC1-Kcro
178kDa
ITA3 SV3
250kDa
180kDa
130kDa
100kDa
70kDa
LAMC1-Kcro
178kDa
ITA1 SV1 ITA2 SV2 ITA3 SV3
250kDa
130kDa
100kDa
70kDa
55kDa
35kDa
LAMC1-Input 178kDa
β-Tubulin 55kDa

Fig. 4c TLN1

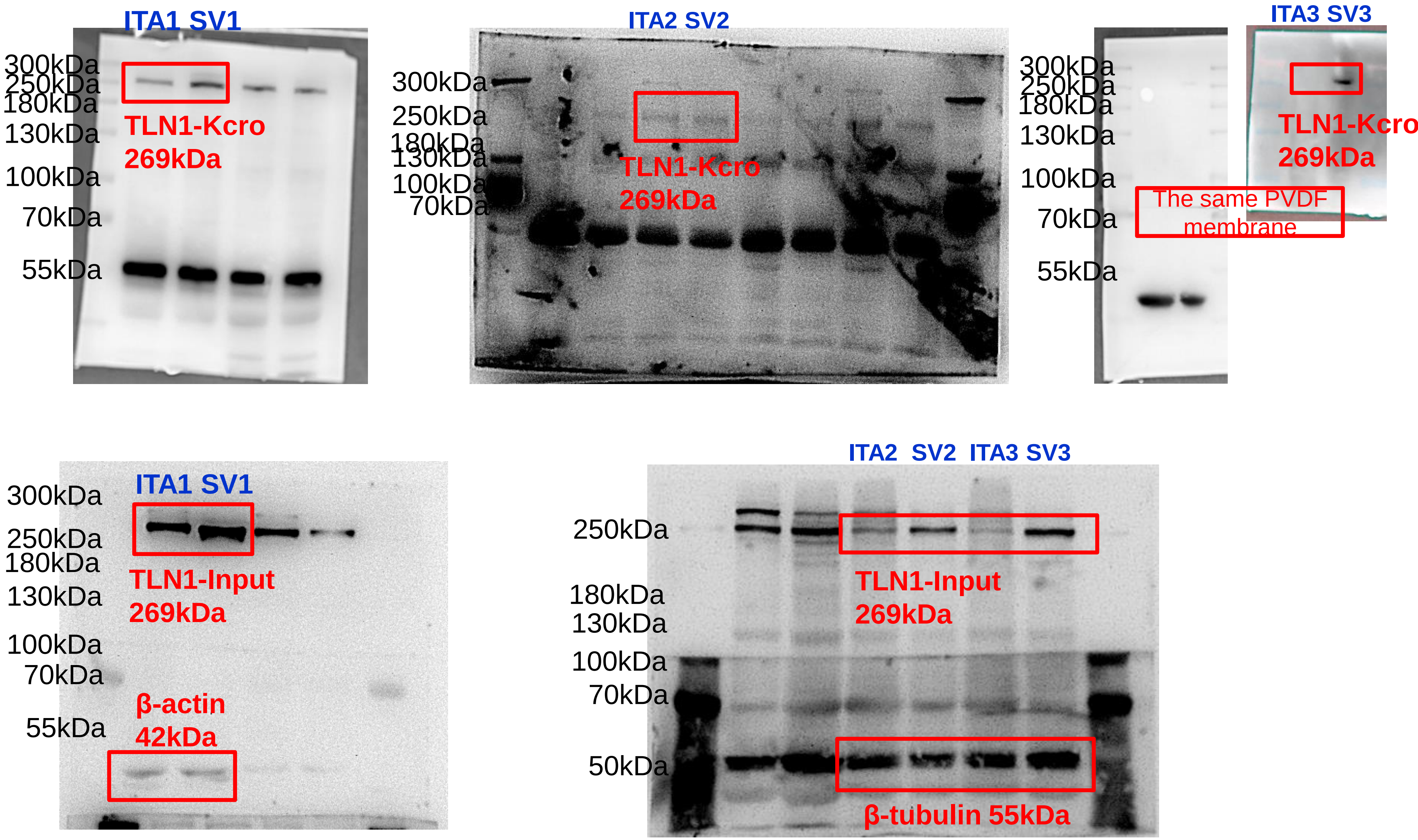

ITA1 SV1
300kDa
250kDa
180kDa
130kDa
100kDa
70kDa
55kDa
TLN1-Kcro
269kDa
ITA2 SV2
300kDa
250kDa
180kDa
130kDa
100kDa
70kDa
TLN1-Kcro
269kDa
ITA3 SV3
300kDa
250kDa
180kDa
130kDa
100kDa
70kDa
55kDa
TLN1-Kcro
269kDa
The same PVDF membrane
ITA1 SV1
300kDa
250kDa
180kDa
130kDa
100kDa
70kDa
55kDa
TLN1-Input
269kDa
β-actin
42kDa
ITA2 SV2 ITA3 SV3
250kDa
180kDa
130kDa
100kDa
70kDa
50kDa
TLN1-Input
269kDa
β-tubulin 55kDa

Fig. 4d TPM1

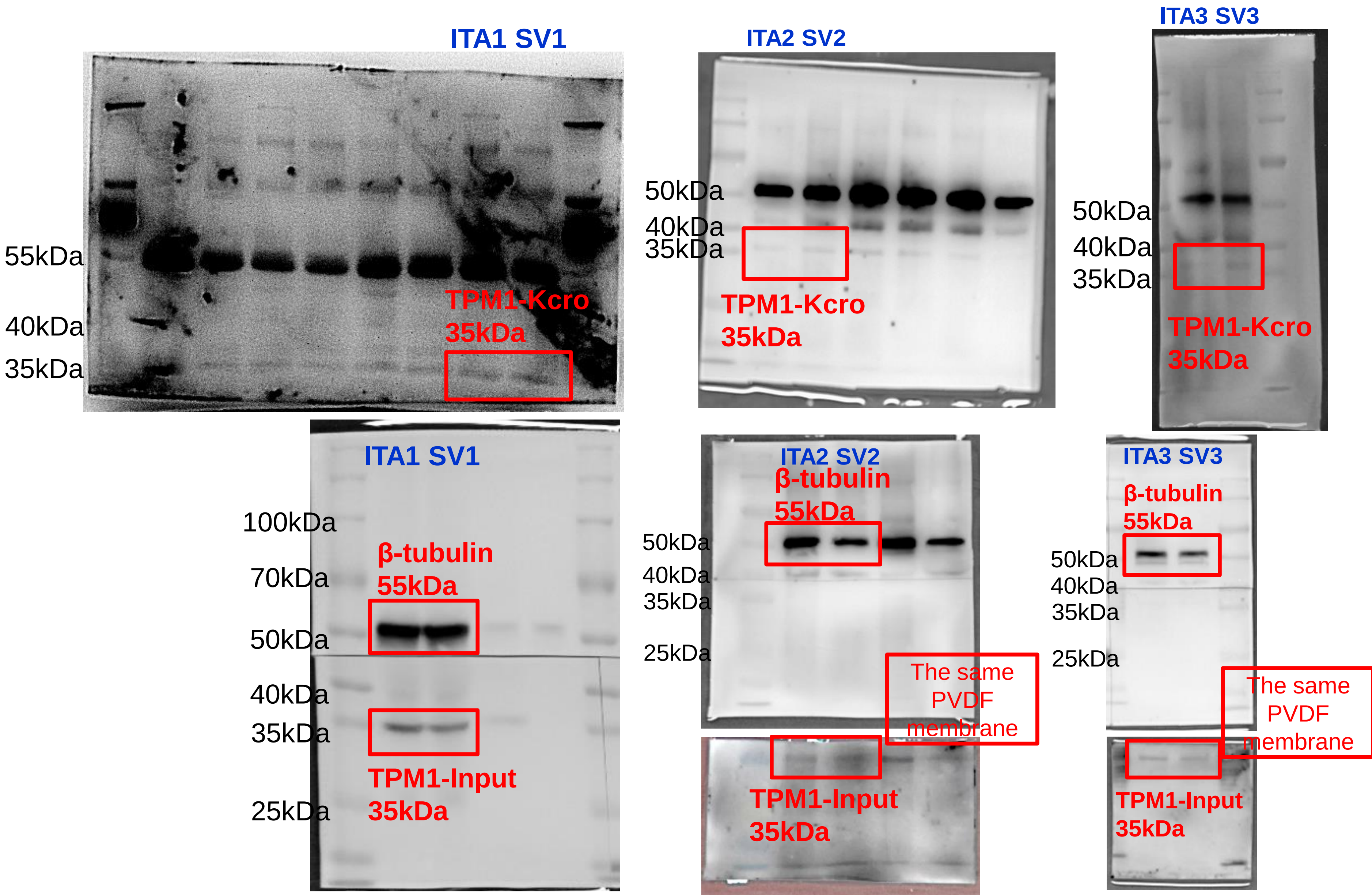

ITA1 SV1
55kDa
40kDa
35kDa
TPM1-Kcro
35kDa
ITA2 SV2
50kDa
40kDa
35kDa
TPM1-Kcro
35kDa
ITA3 SV3
50kDa
40kDa
35kDa
TPM1-Kcro
35kDa
ITA1 SV1
100kDa
70kDa
50kDa
40kDa
35kDa
25kDa
β-tubulin
55kDa
TPM1-Input
35kDa
ITA2 SV2
β-tubulin
55kDa
50kDa
40kDa
35kDa
25kDa
The same PVDF membrane
TPM1-Input
35kDa
ITA3 SV3
β-tubulin
55kDa
50kDa
40kDa
35kDa
25kDa
The same PVDF membrane
TPM1-Input
35kDa

Fig. 4e TPM2

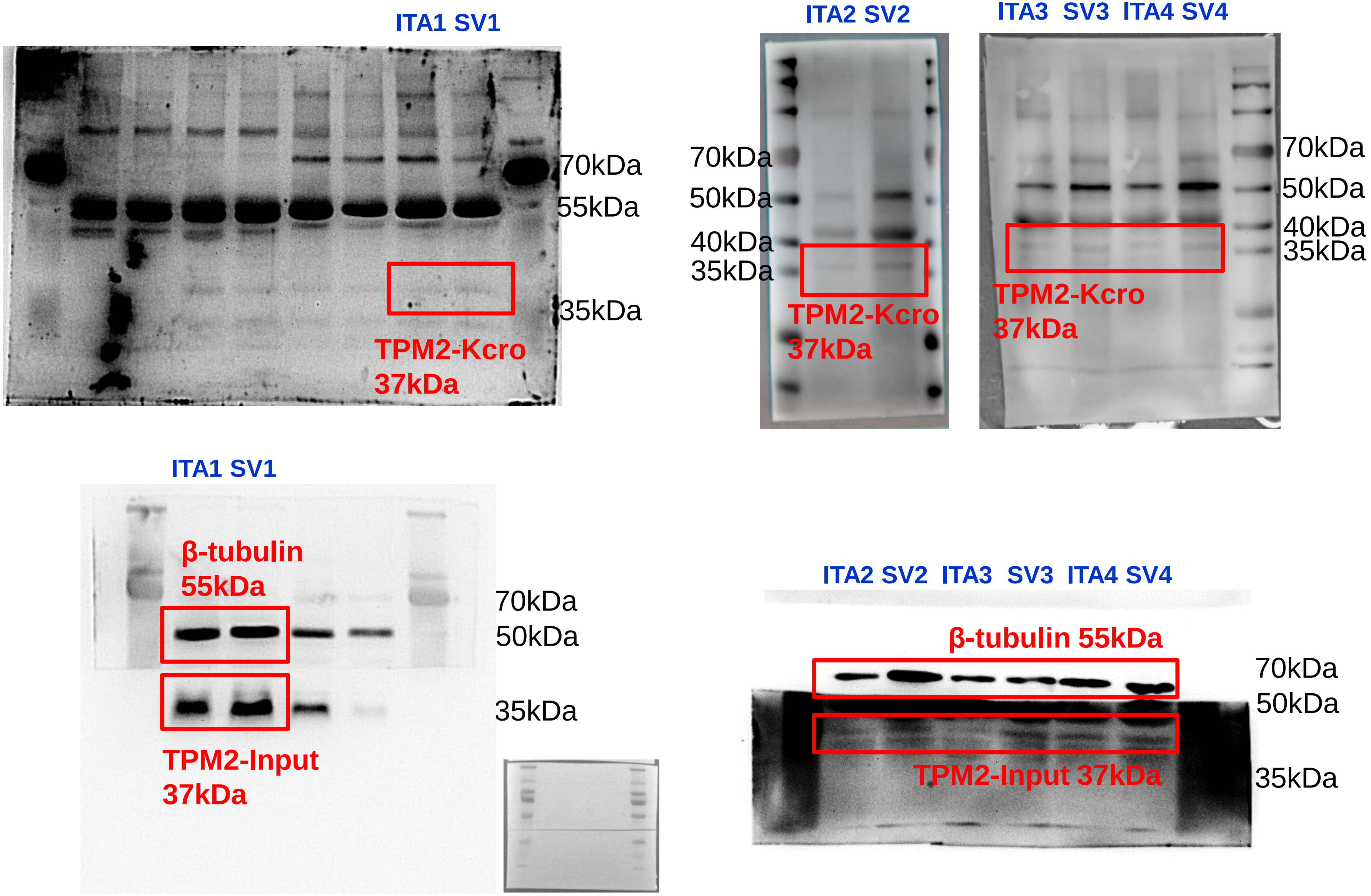
ITA1 SV1
70kDa
55kDa
35kDa
TPM2-Kcro
37kDa
ITA2 SV2
70kDa
50kDa
40kDa
35kDa
TPM2-Kcro
37kDa
ITA3 SV3 ITA4 SV4
70kDa
50kDa
40kDa
35kDa
TPM2-Kcro
37kDa
ITA1 SV1
β-tubulin
55kDa
70kDa
50kDa
35kDa
TPM2-Input
37kDa
ITA2 SV2 ITA3 SV3 ITA4 SV4
β-tubulin 55kDa
70kDa
50kDa
TPM2-Input 37kDa
35kDa

Fig. 4f TPM3

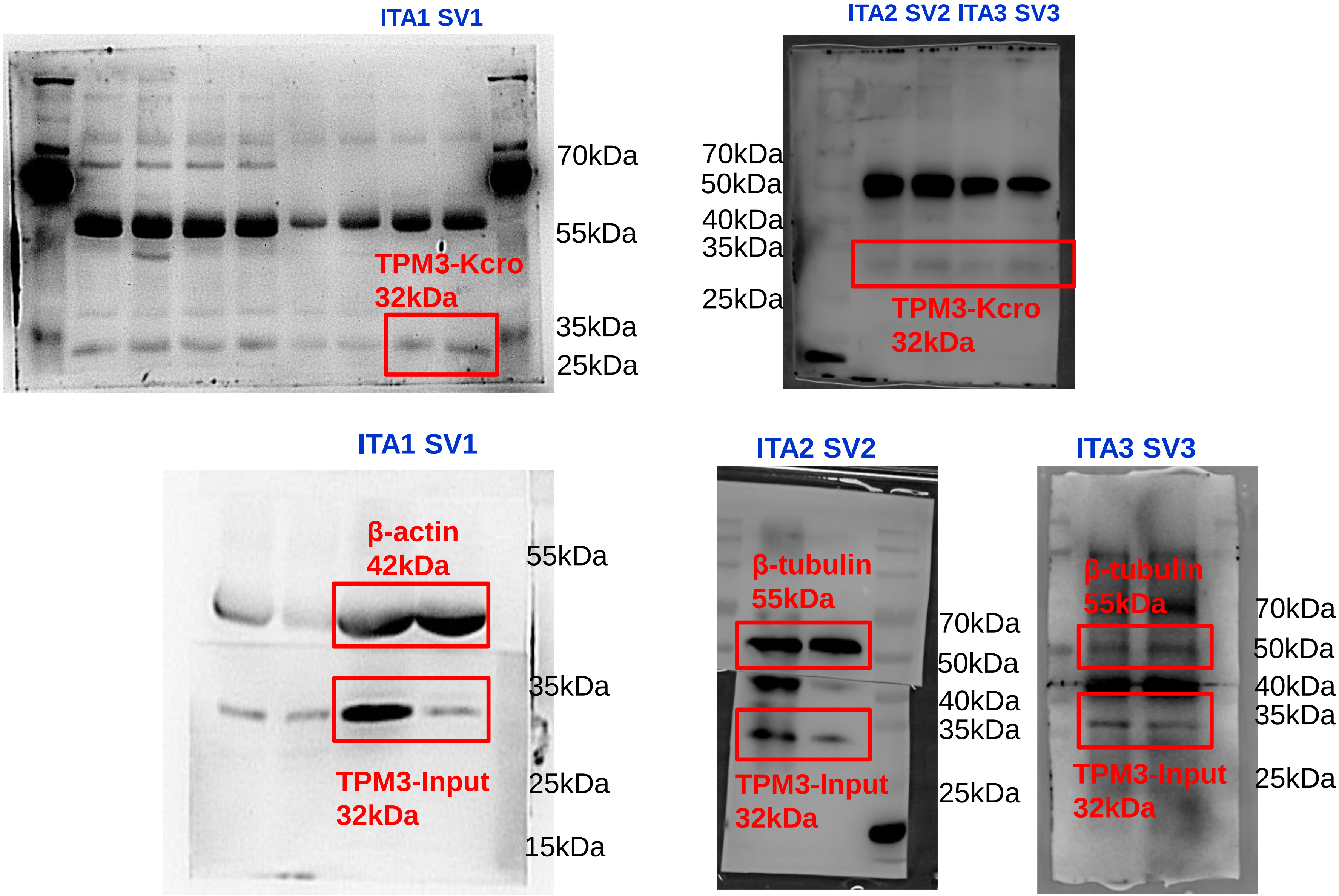
ITA1 SV1
70kDa
55kDa
TPM3-Kcro
32kDa
35kDa
25kDa
ITA2 SV2 ITA3 SV3
70kDa
50kDa
40kDa
35kDa
25kDa
TPM3-Kcro
32kDa
ITA1 SV1
β-actin
42kDa
55kDa
35kDa
TPM3-Input
32kDa
25kDa
15kDa
ITA2 SV2
β-tubulin
55kDa
70kDa
50kDa
40kDa
35kDa
TPM3-Input
32kDa
25kDa
ITA3 SV3
β-tubulin
55kDa
70kDa
50kDa
40kDa
35kDa
TPM3-Input
32kDa
25kDa

Fig. 4g TPM4

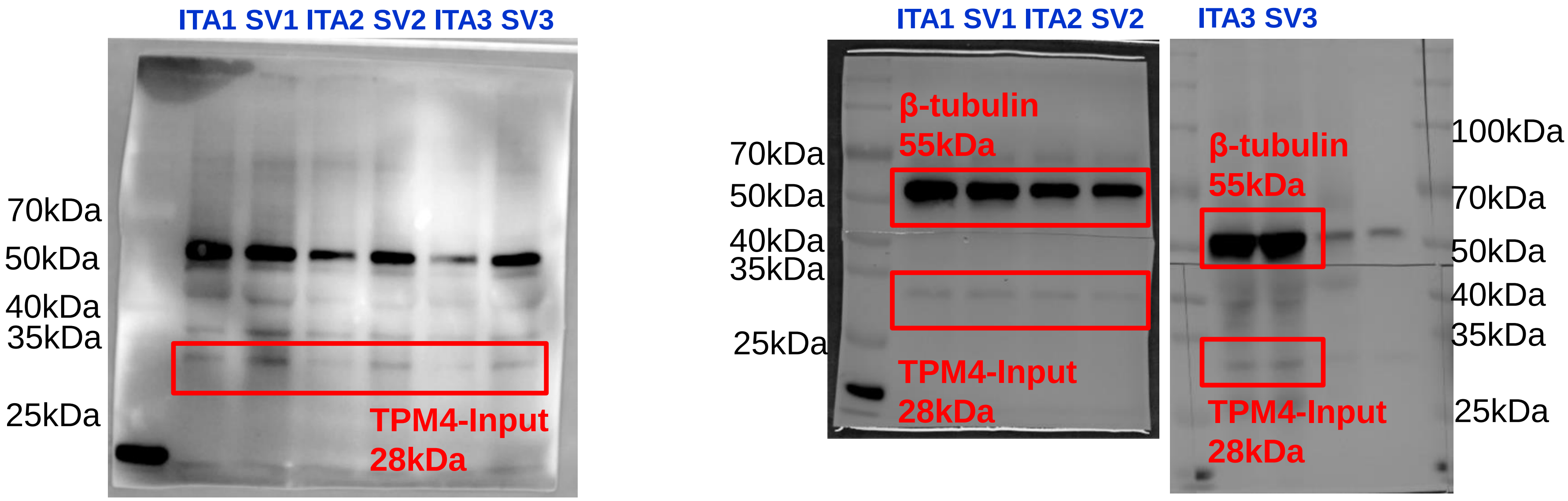
ITA1 SV1 ITA2 SV2 ITA3 SV3
70kDa
50kDa
40kDa
35kDa
25kDa
TPM4-Input
28kDa
ITA1 SV1 ITA2 SV2
ITA3 SV3
β-tubulin
55kDa
70kDa
50kDa
40kDa
35kDa
25kDa
TPM4-Input
28kDa
β-tubulin
55kDa
100kDa
70kDa
50kDa
40kDa
35kDa
25kDa
TPM4-Input
28kDa

Fig. 4h TXN

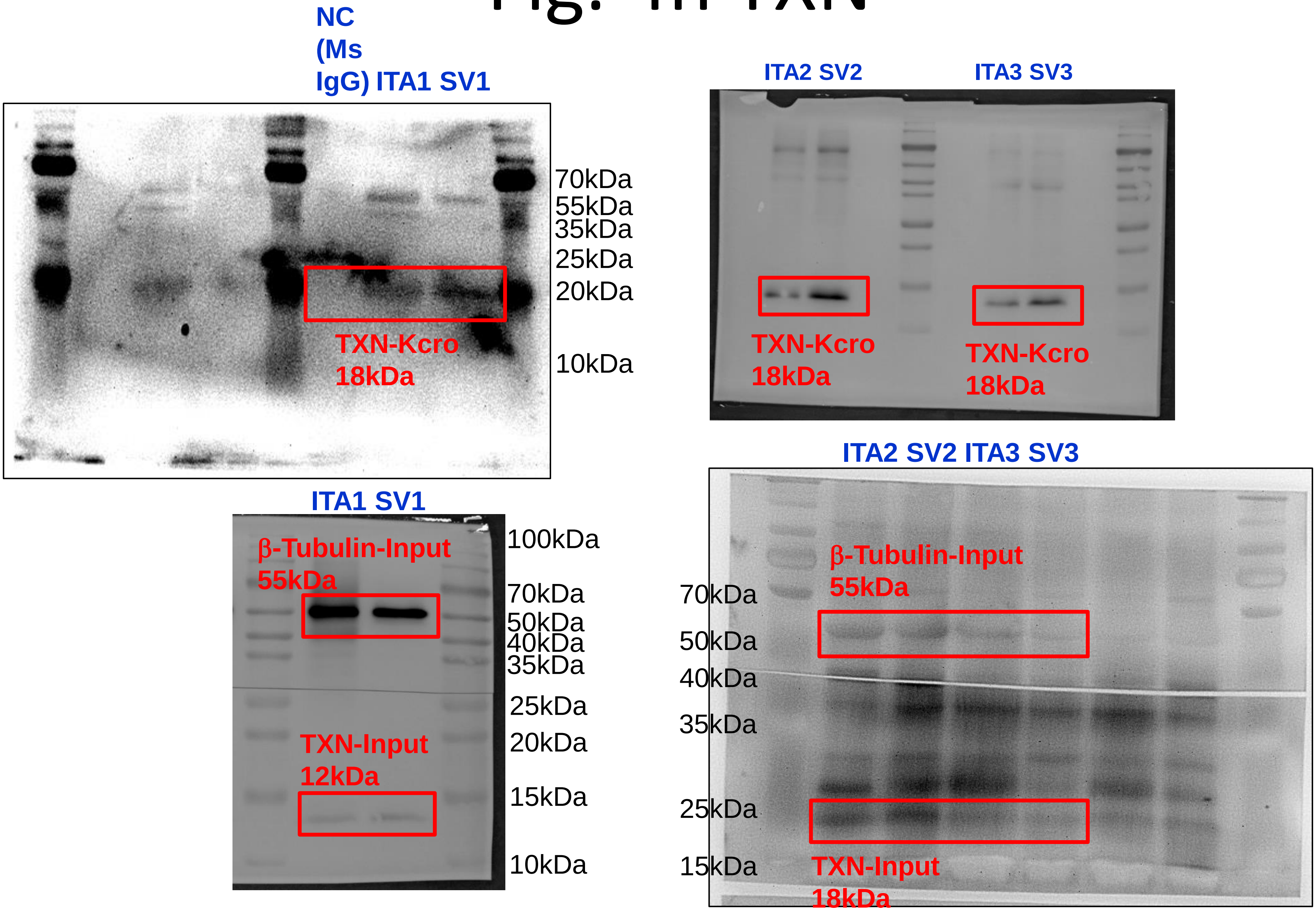
NC
(Ms
IgG) ITA1 SV1
70kDa
55kDa
35kDa
25kDa
20kDa
10kDa
TXN-Kcro
18kDa
ITA2 SV2
ITA3 SV3
TXN-Kcro
18kDa
TXN-Kcro
18kDa
ITA1 SV1
β-Tubulin-Input
55kDa
100kDa
70kDa
50kDa
40kDa
35kDa
25kDa
20kDa
15kDa
10kDa
TXN-Input
12kDa
ITA2 SV2 ITA3 SV3
β-Tubulin-Input
55kDa
70kDa
50kDa
40kDa
35kDa
25kDa
15kDa
TXN-Input
18kDa

Fig. 4i GLO1

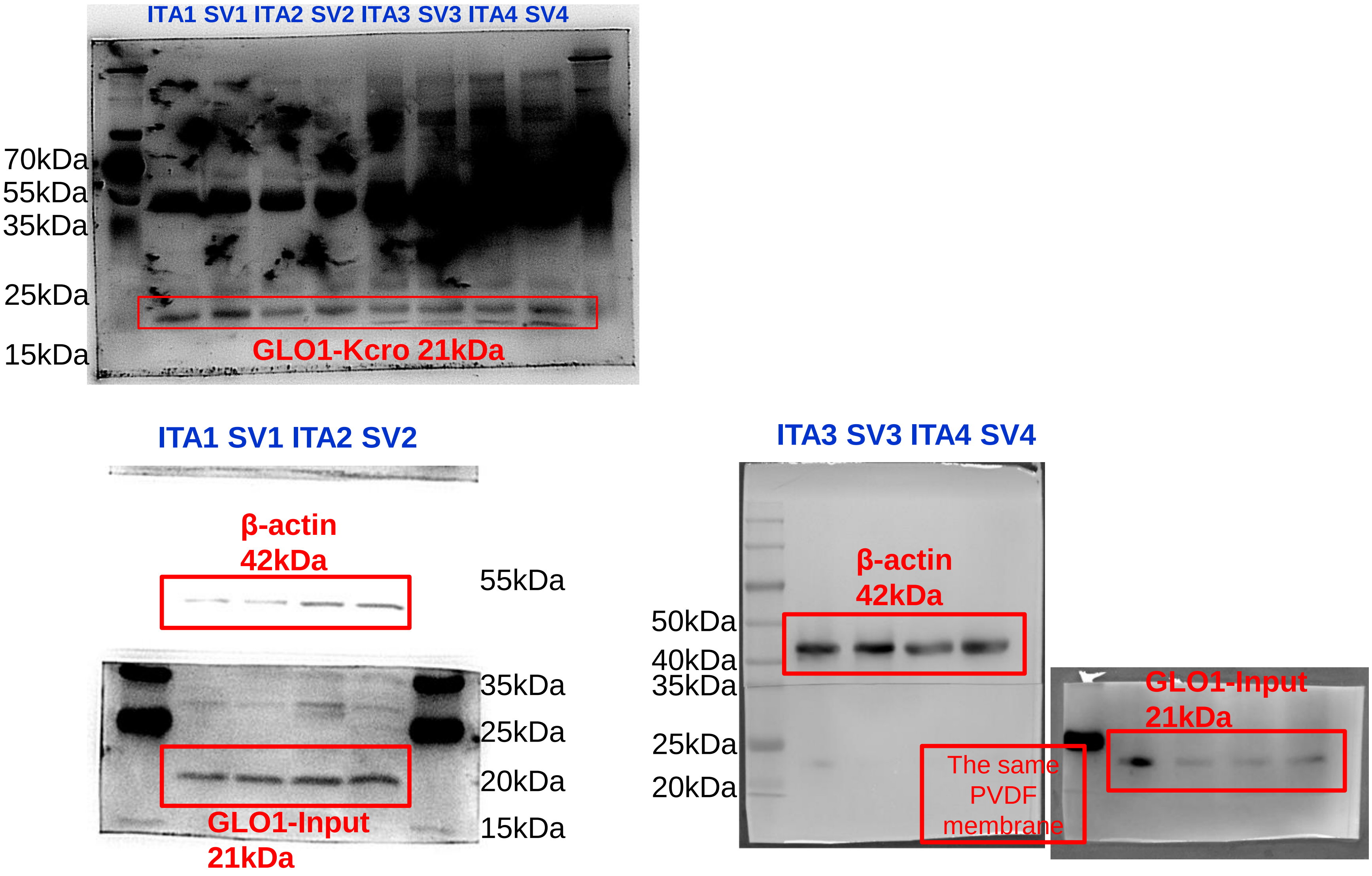
ITA1 SV1 ITA2 SV2 ITA3 SV3 ITA4 SV4
70kDa
55kDa
35kDa
25kDa
15kDa
GLO1-Kcro 21kDa
ITA1 SV1 ITA2 SV2
β-actin
42kDa
55kDa
35kDa
25kDa
20kDa
15kDa
GLO1-Input
21kDa
ITA3 SV3 ITA4 SV4
β-actin
42kDa
50kDa
40kDa
35kDa
25kDa
20kDa
The same
PVDF
membrane
GLO1-Input
21kDa

Fig. 4j GAPDH

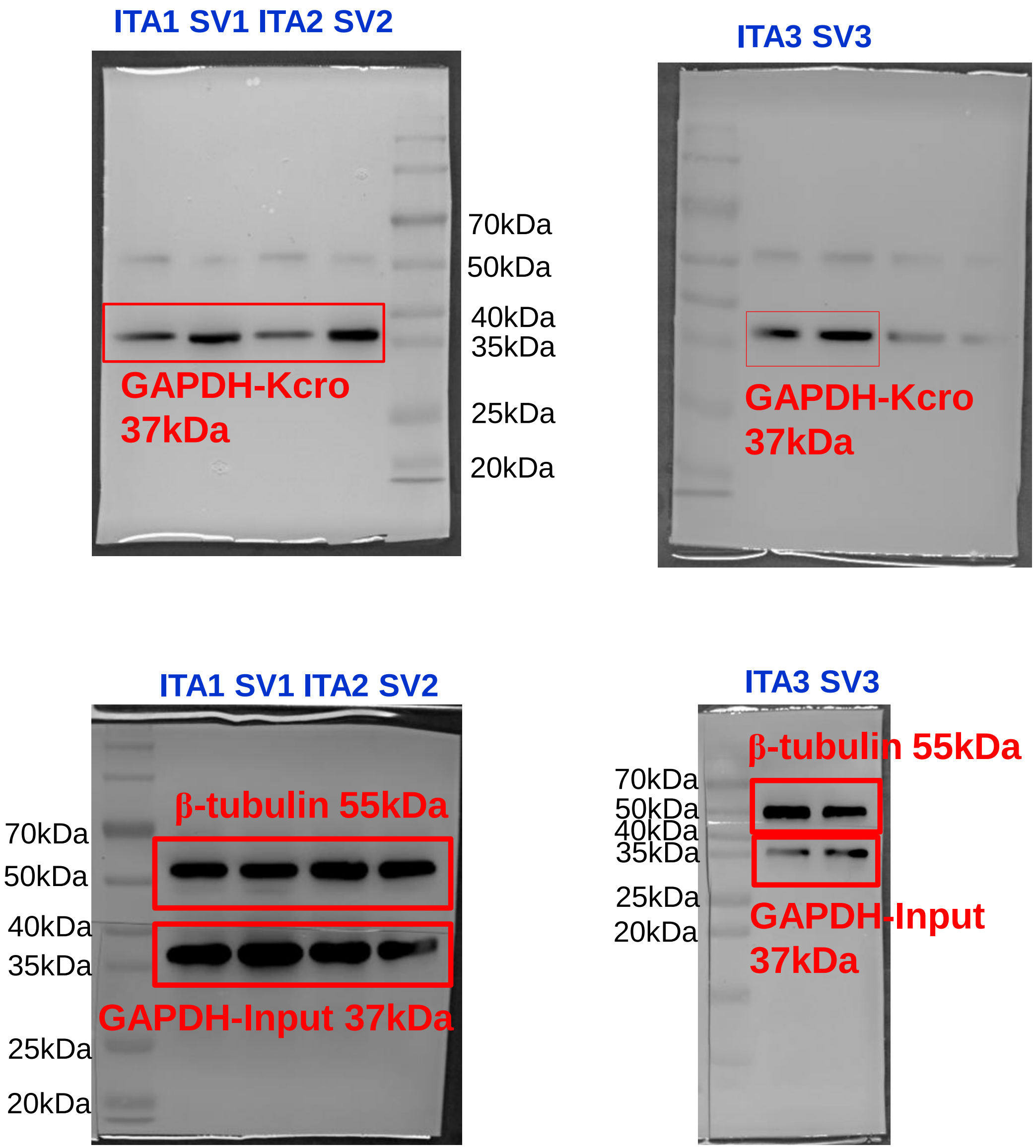

ITA1 SV1 ITA2 SV2
70kDa
50kDa
40kDa
35kDa
GAPDH-Kcro
37kDa
25kDa
20kDa
ITA3 SV3
GAPDH-Kcro
37kDa
ITA1 SV1 ITA2 SV2
70kDa
β-tubulin 55kDa
50kDa
40kDa
35kDa
GAPDH-Input 37kDa
25kDa
20kDa
ITA3 SV3
β-tubulin 55kDa
70kDa
50kDa
40kDa
35kDa
25kDa
20kDa
GAPDH-Input
37kDa

Fig. 4k ALDH2

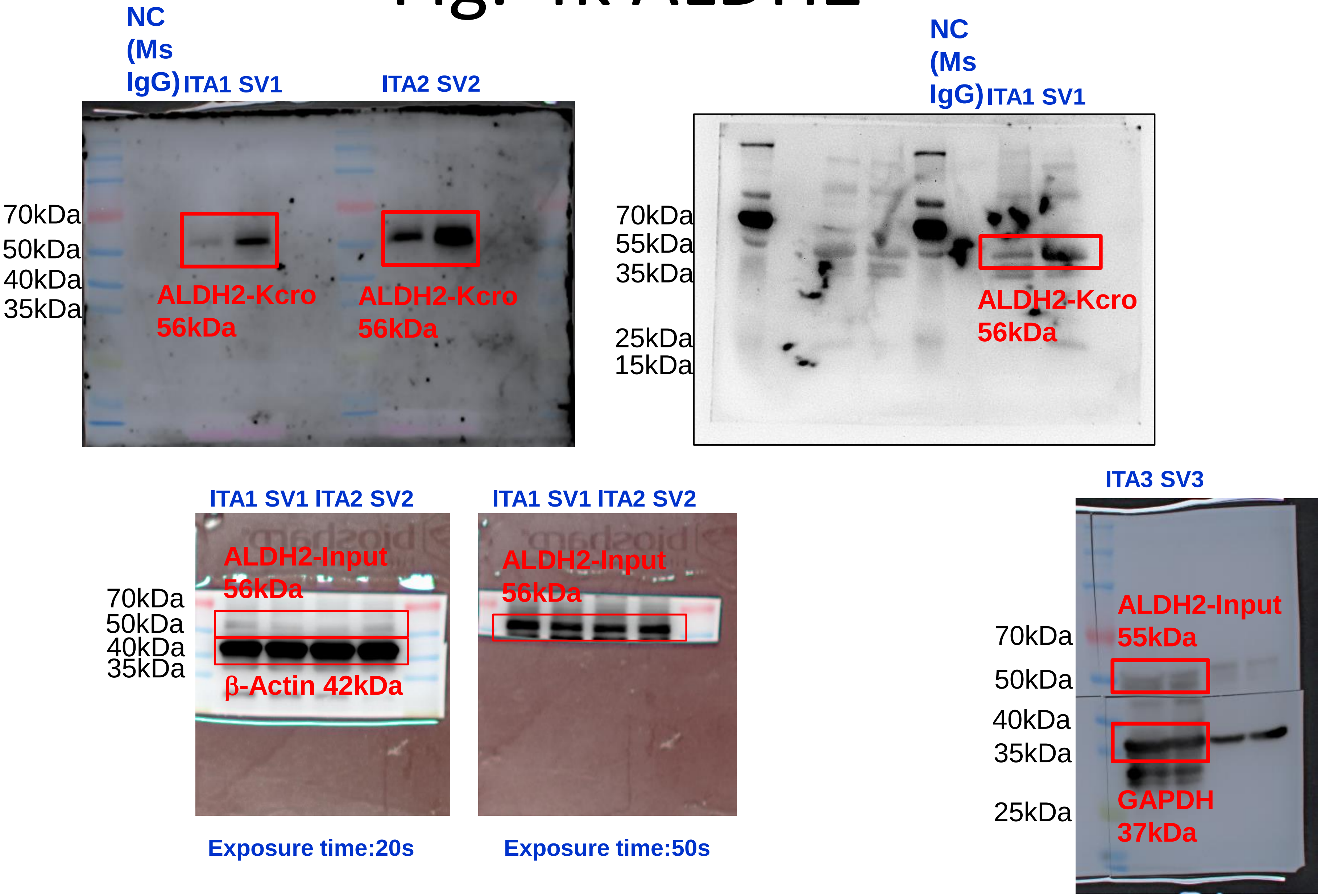
NC (Ms IgG)
ITA1 SV1
ITA2 SV2
70kDa
50kDa
40kDa
35kDa
ALDH2-Kcro 56kDa
ALDH2-Kcro 56kDa
NC (Ms IgG)
ITA1 SV1
70kDa
55kDa
35kDa
25kDa
15kDa
ALDH2-Kcro 56kDa
ITA1 SV1 ITA2 SV2
70kDa
50kDa
40kDa
35kDa
ALDH2-Input 56kDa
β-Actin 42kDa
Exposure time:20s
ITA1 SV1 ITA2 SV2
ALDH2-Input 56kDa
Exposure time:50s
ITA3 SV3
70kDa
50kDa
40kDa
35kDa
25kDa
ALDH2-Input 55kDa
GAPDH 37kDa

Fig. 5c

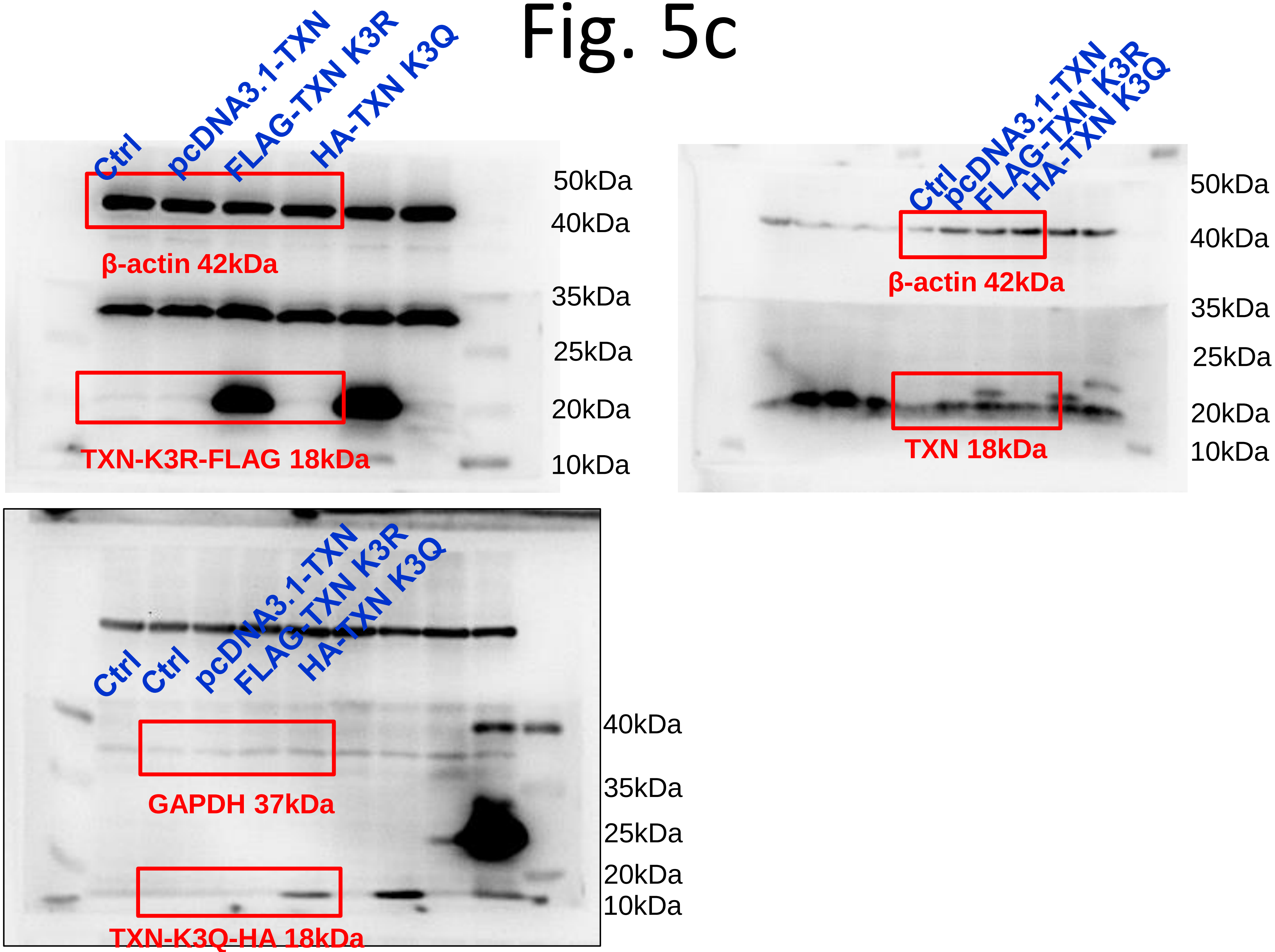

Ctrl
pcDNA3.1-TXN
FLAG-TXN K3R
HA-TXN K3Q
50kDa
40kDa
β-actin 42kDa
35kDa
25kDa
20kDa
TXN-K3R-FLAG 18kDa
10kDa
Ctrl
pcDNA3.1-TXN
FLAG-TXN K3R
HA-TXN K3Q
50kDa
40kDa
β-actin 42kDa
35kDa
25kDa
20kDa
TXN 18kDa
10kDa
Ctrl
Ctrl
pcDNA3.1-TXN
FLAG-TXN K3R
HA-TXN K3Q
40kDa
GAPDH 37kDa
35kDa
25kDa
20kDa
10kDa
TXN-K3Q-HA 18kDa

Fig. 6c

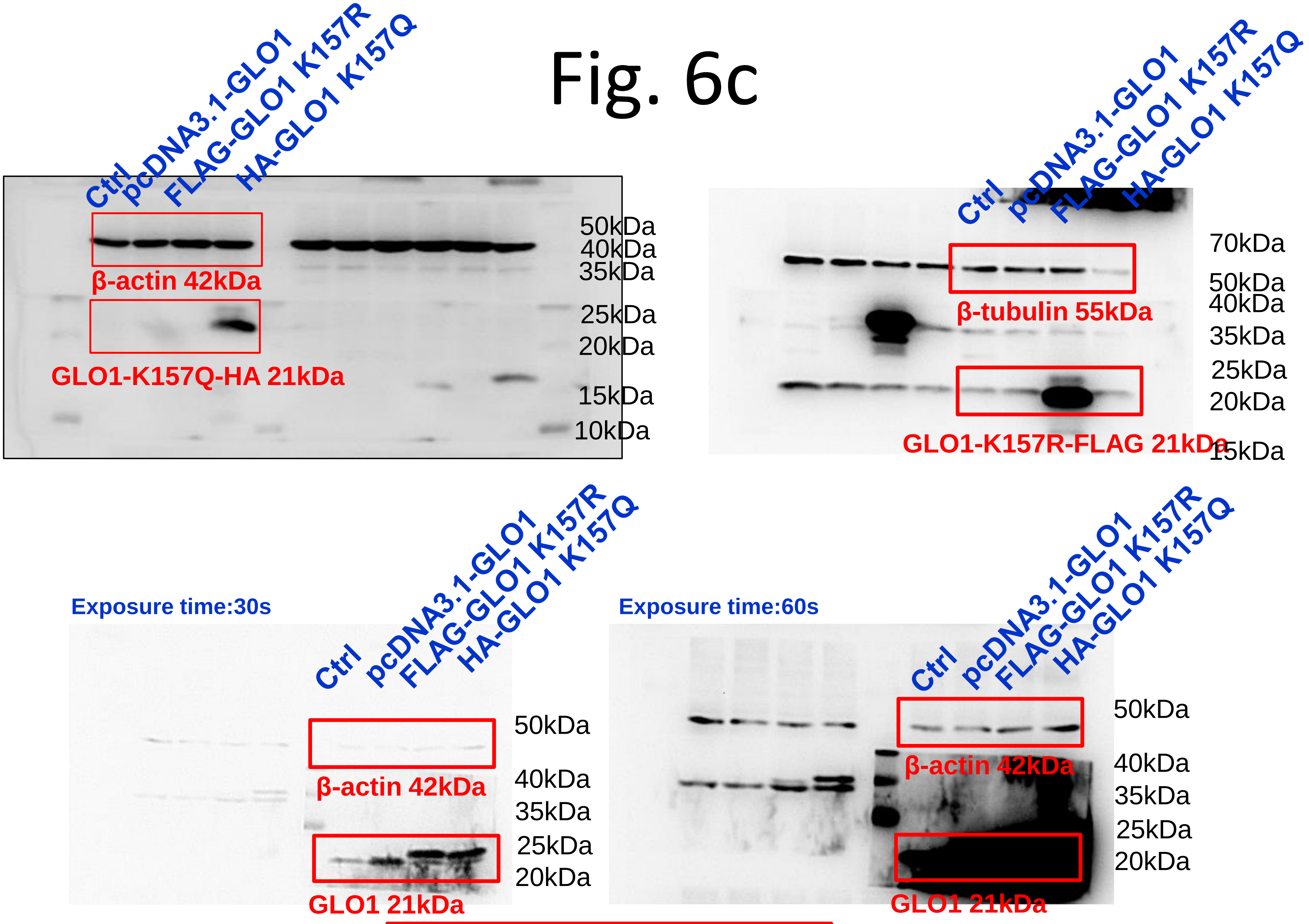

The same PVDF membrane

Fig. 7c

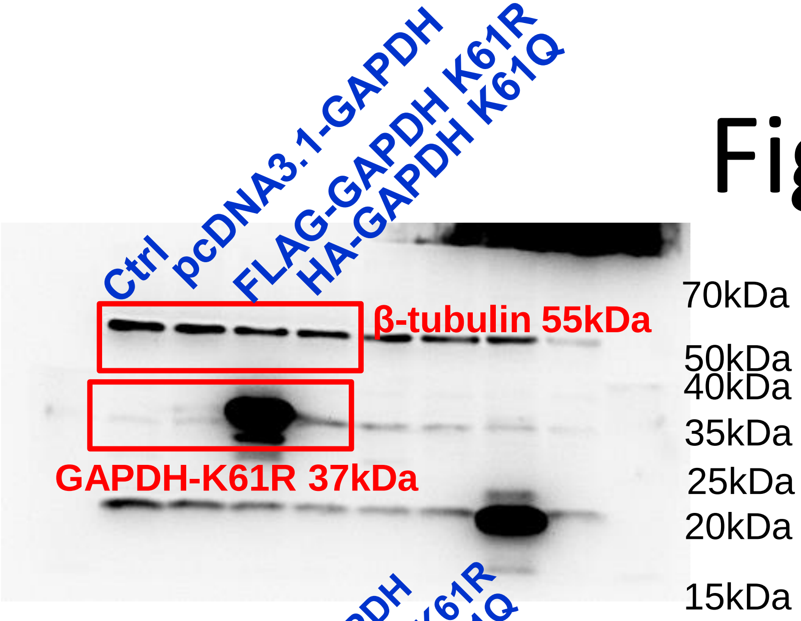

Ctrl
pcDNA3.1-GAPDH
FLAG-GAPDH K61R
HA-GAPDH K61Q
β-tubulin 55kDa
GAPDH-K61R 37kDa
70kDa
50kDa
40kDa
35kDa
25kDa
20kDa
15kDa

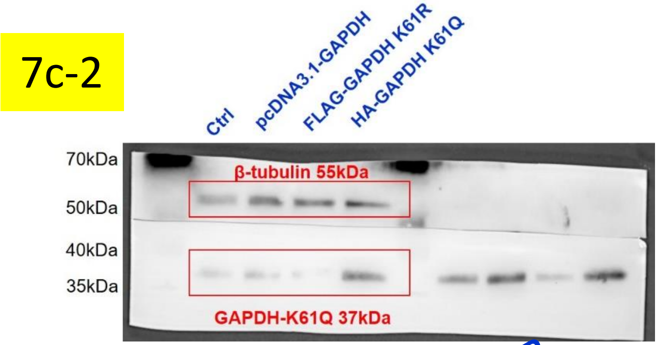

7c-2
Ctrl
pcDNA3.1-GAPDH
FLAG-GAPDH K61R
HA-GAPDH K61Q
70kDa
50kDa
40kDa
35kDa
β-tubulin 55kDa
GAPDH-K61Q 37kDa

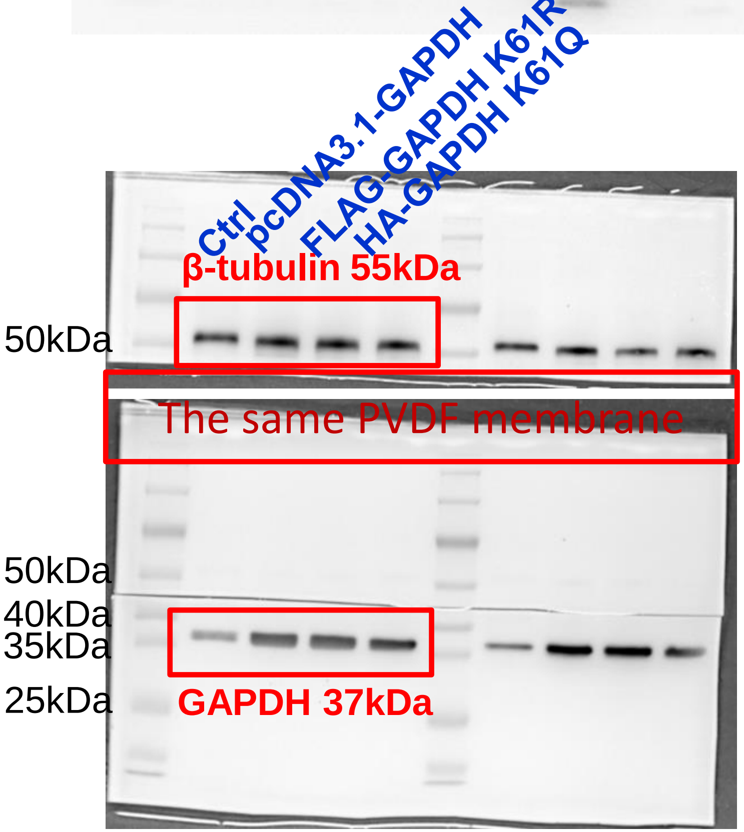

Ctrl
pcDNA3.1-GAPDH
FLAG-GAPDH K61R
HA-GAPDH K61Q
β-tubulin 55kDa
50kDa
The same PVDF membrane
50kDa
40kDa
35kDa
25kDa
GAPDH 37kDa

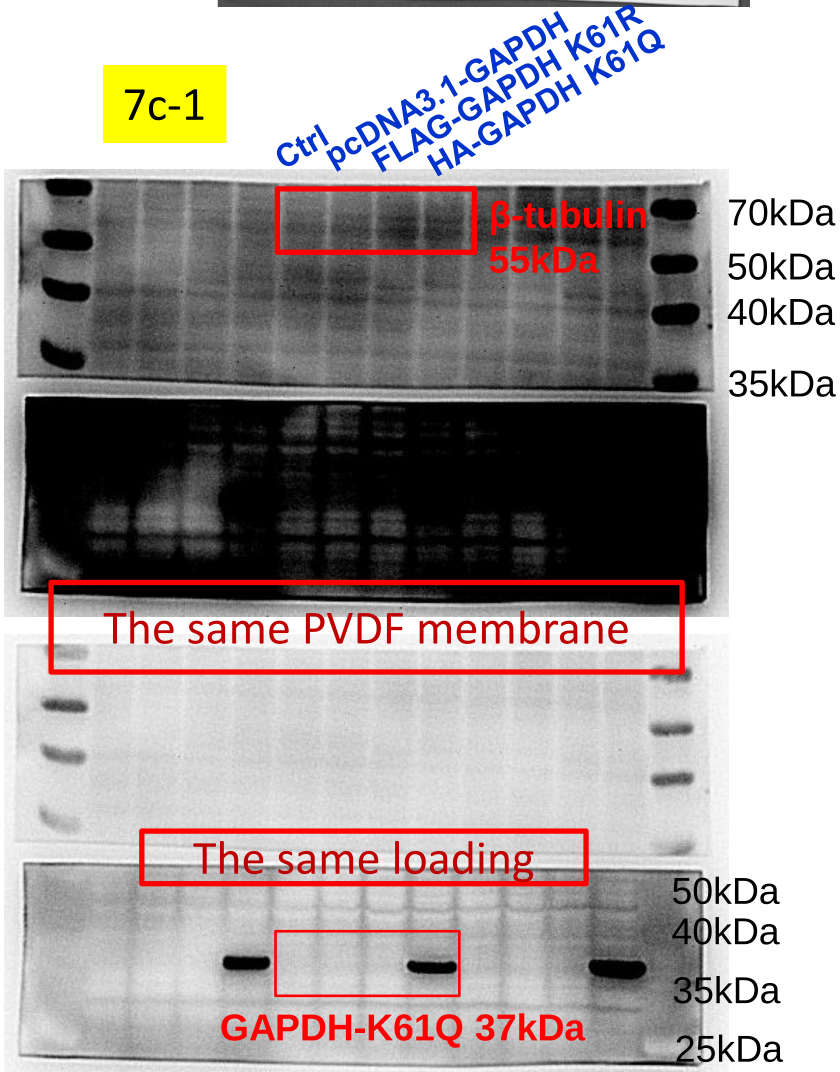

7c-1
Ctrl
pcDNA3.1-GAPDH
FLAG-GAPDH K61R
HA-GAPDH K61Q
β-tubulin 55kDa
70kDa
50kDa
40kDa
35kDa
The same PVDF membrane
The same loading
50kDa
40kDa
35kDa
25kDa
GAPDH-K61Q 37kDa

Fig. 8b

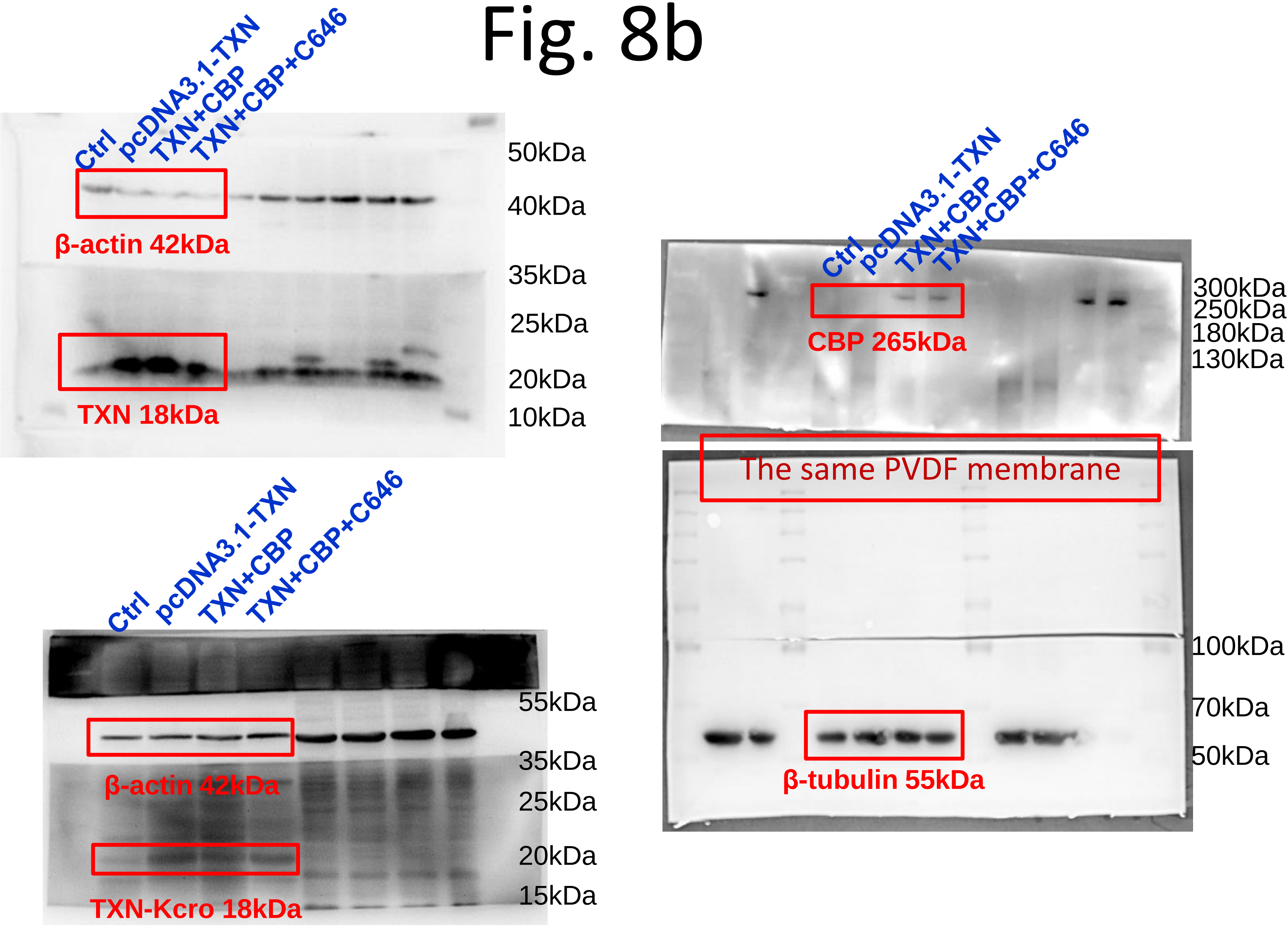

Ctrl
pcDNA3.1-TXN
TXN+CBP
TXN+CBP+C646
50kDa
40kDa
β-actin 42kDa
35kDa
25kDa
20kDa
TXN 18kDa
10kDa
Ctrl
pcDNA3.1-TXN
TXN+CBP
TXN+CBP+C646
300kDa
250kDa
180kDa
130kDa
CBP 265kDa
The same PVDF membrane
100kDa
70kDa
50kDa
β-tubulin 55kDa
Ctrl
pcDNA3.1-TXN
TXN+CBP
TXN+CBP+C646
55kDa
35kDa
β-actin 42kDa
25kDa
20kDa
15kDa
TXN-Kcro 18kDa

Fig. 8c

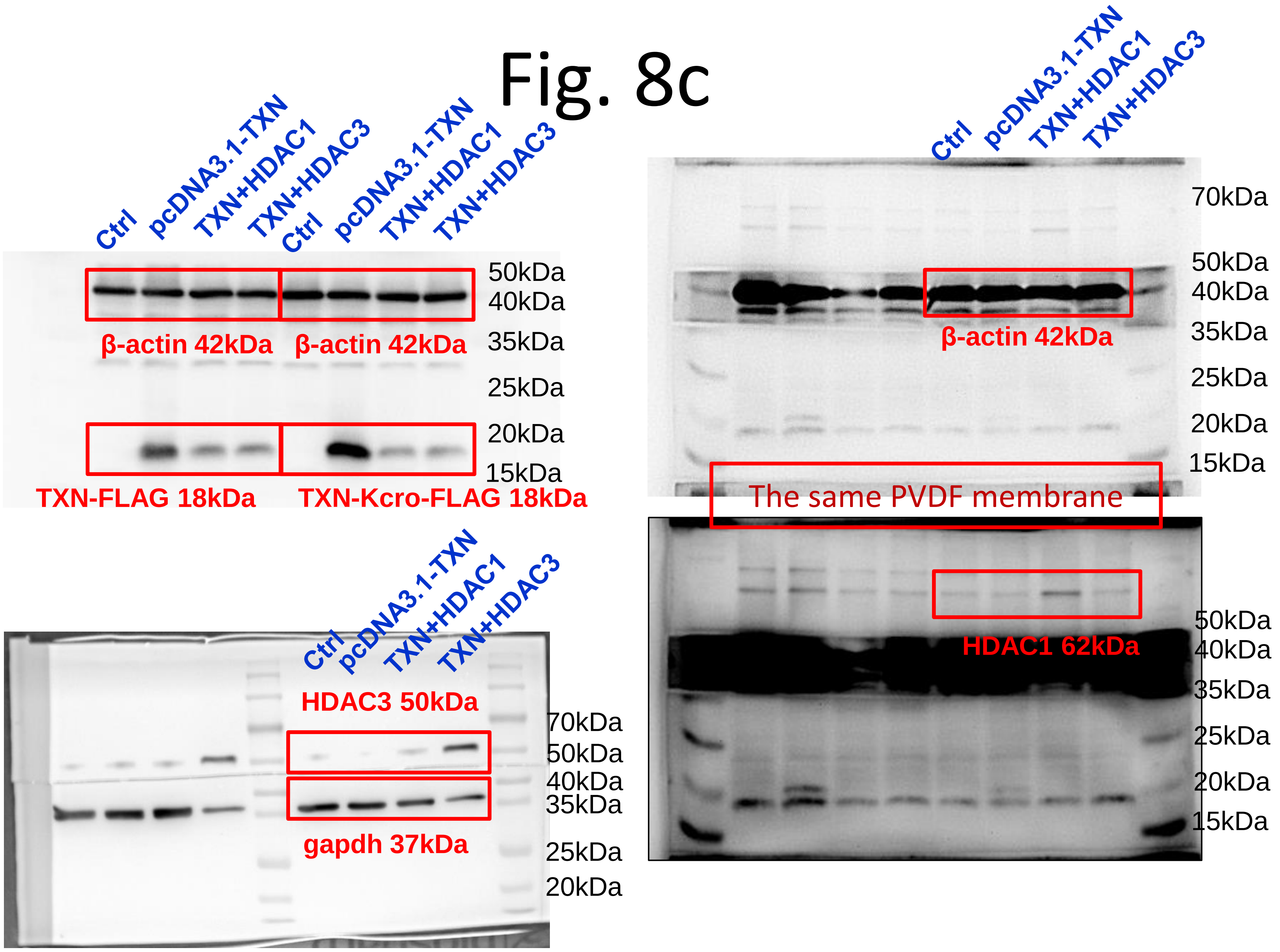

Ctrl
pcDNA3.1-TXN
TXN+HDAC1
TXN+HDAC3
Ctrl
pcDNA3.1-TXN
TXN+HDAC1
TXN+HDAC3
50kDa
40kDa
β-actin 42kDa
β-actin 42kDa
35kDa
25kDa
20kDa
15kDa
TXN-FLAG 18kDa
TXN-Kcro-FLAG 18kDa
Ctrl
pcDNA3.1-TXN
TXN+HDAC1
TXN+HDAC3
70kDa
50kDa
40kDa
β-actin 42kDa
35kDa
25kDa
20kDa
15kDa
The same PVDF membrane
HDAC1 62kDa
50kDa
40kDa
35kDa
25kDa
20kDa
15kDa
Ctrl
pcDNA3.1-TXN
TXN+HDAC1
TXN+HDAC3
HDAC3 50kDa
gapdh 37kDa
70kDa
50kDa
40kDa
35kDa
25kDa
20kDa

Fig. 8e

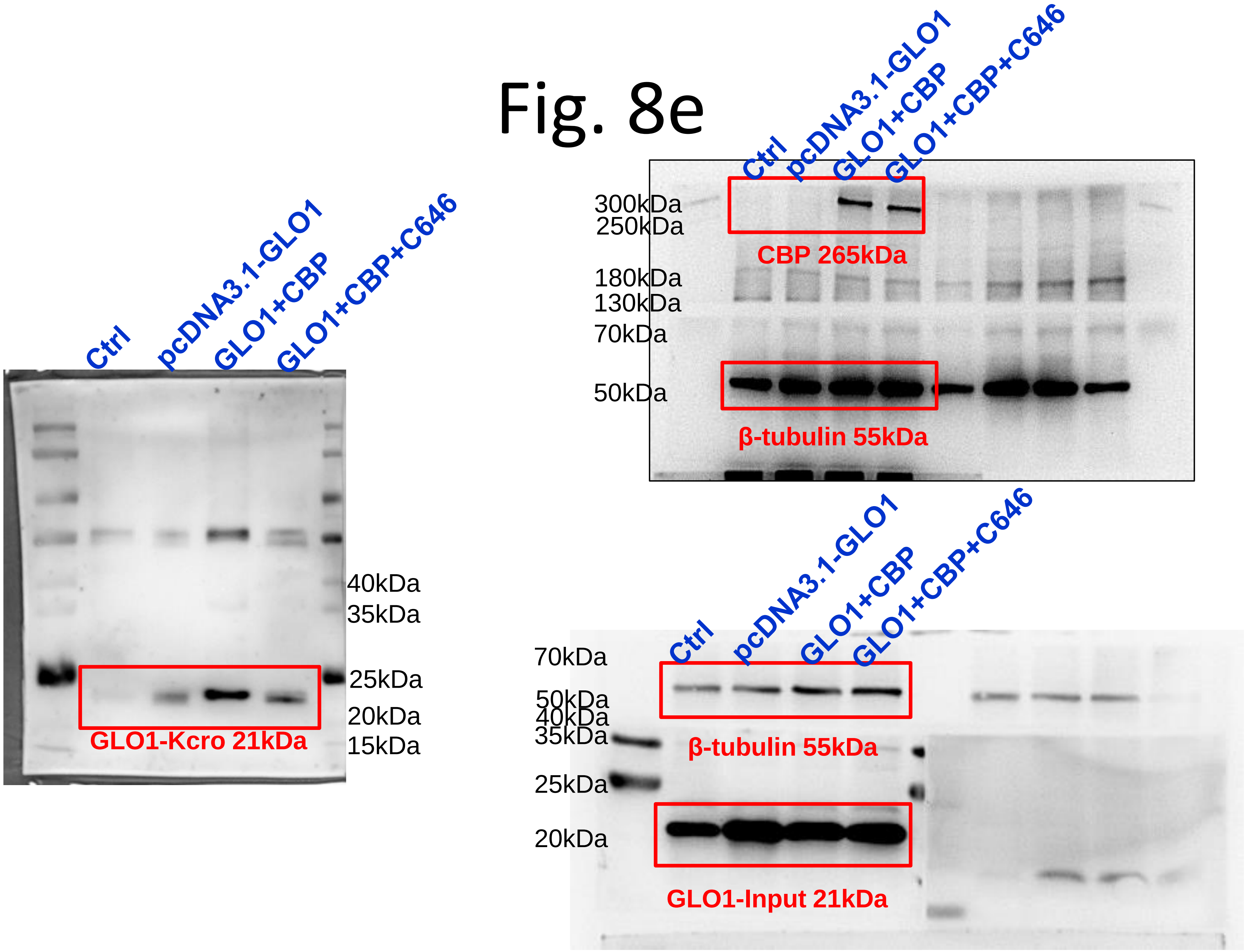

Ctrl
pcDNA3.1-GLO1
GLO1+CBP
GLO1+CBP+C646
40kDa
35kDa
25kDa
20kDa
15kDa
GLO1-Kcro 21kDa
Ctrl
pcDNA3.1-GLO1
GLO1+CBP
GLO1+CBP+C646
300kDa
250kDa
180kDa
130kDa
70kDa
50kDa
CBP 265kDa
β-tubulin 55kDa
Ctrl
pcDNA3.1-GLO1
GLO1+CBP
GLO1+CBP+C646
70kDa
50kDa
40kDa
35kDa
25kDa
20kDa
β-tubulin 55kDa
GLO1-Input 21kDa

Fig. 8f

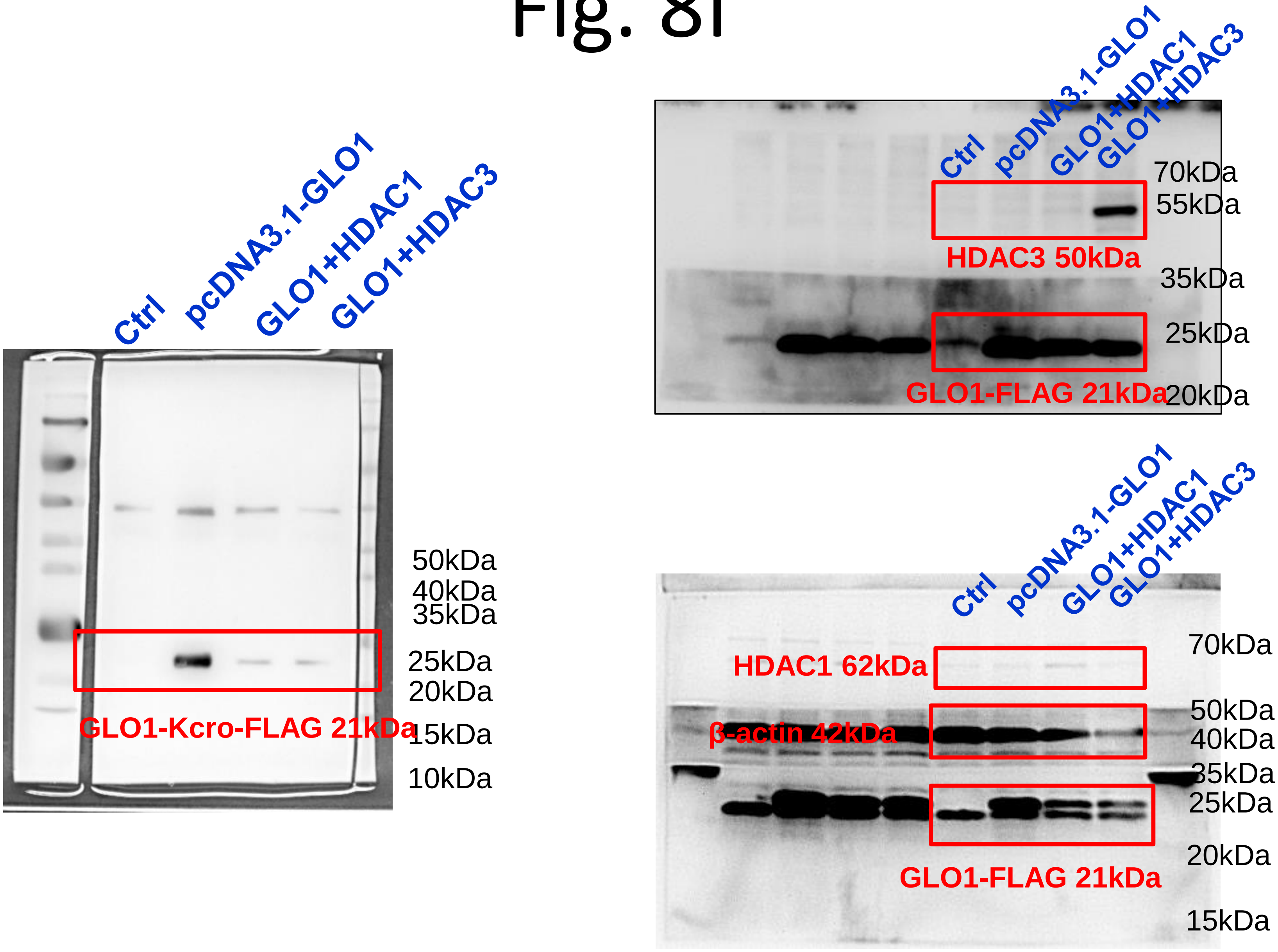

Ctrl
pcDNA3.1-GLO1
GLO1+HDAC1
GLO1+HDAC3
50kDa
40kDa
35kDa
25kDa
20kDa
GLO1-Kcro-FLAG 21kDa
15kDa
10kDa
Ctrl
pcDNA3.1-GLO1
GLO1+HDAC1
GLO1+HDAC3
70kDa
55kDa
HDAC3 50kDa
35kDa
25kDa
GLO1-FLAG 21kDa
20kDa
Ctrl
pcDNA3.1-GLO1
GLO1+HDAC1
GLO1+HDAC3
70kDa
HDAC1 62kDa
50kDa
β-actin 42kDa
40kDa
35kDa
25kDa
20kDa
GLO1-FLAG 21kDa
15kDa

Fig. 8h

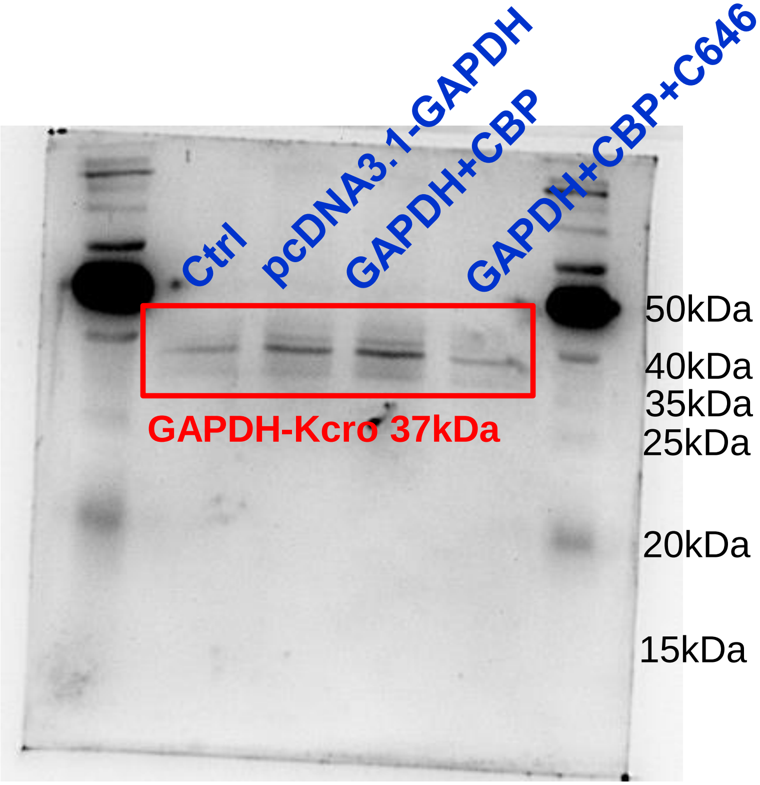
Ctrl
pcDNA3.1-GAPDH
GAPDH+CBP
GAPDH+CBP+C646
50kDa
40kDa
35kDa
25kDa
20kDa
15kDa
GAPDH-Kcro 37kDa

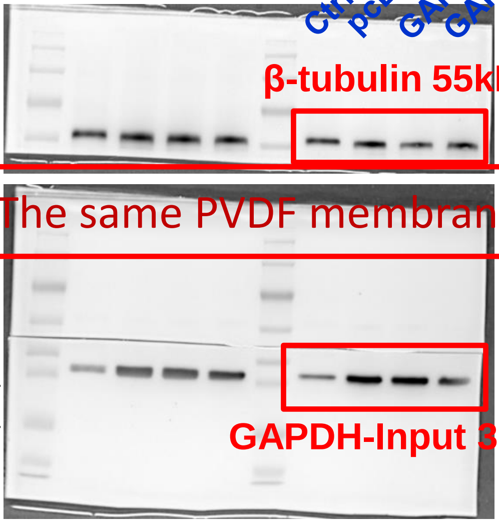
Ctrl
pcDNA3.1-GAPDH
GAPDH+CBP
GAPDH+CBP+C646
β-tubulin 55kDa
50kDa
The same PVDF membrane
50kDa
40kDa
35kDa
25kDa
GAPDH-Input 37kDa

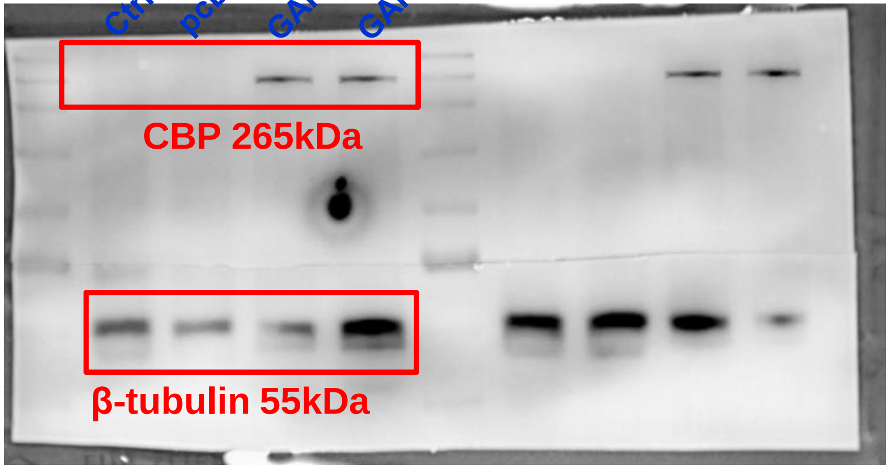
Ctrl
pcDNA3.1-GAPDH
GAPDH+CBP
GAPDH+CBP+C646
300kDa
250kDa
130kDa
100kDa
70kDa
50kDa
CBP 265kDa
β-tubulin 55kDa

Fig. 8i

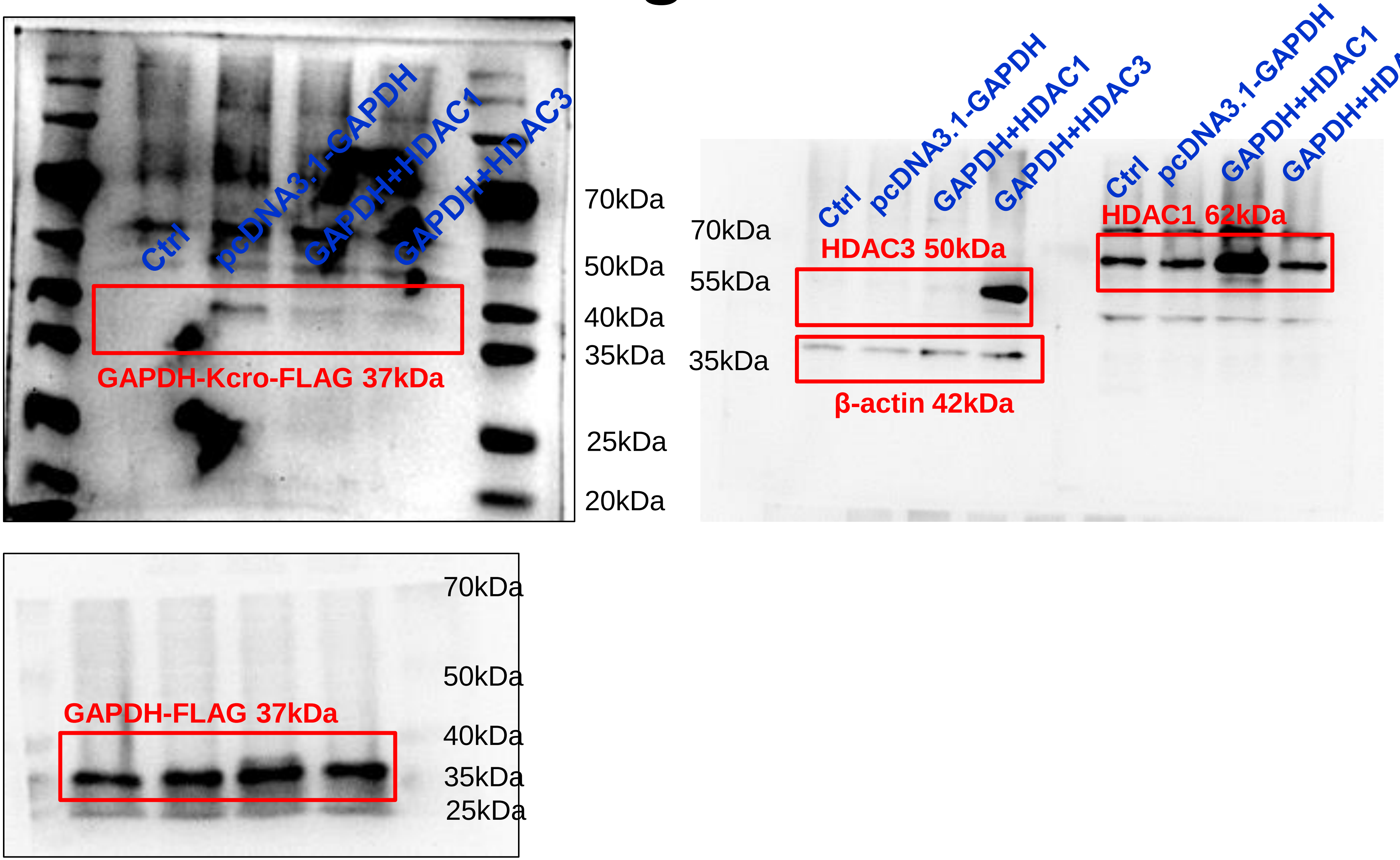

Ctrl
pcDNA3.1-GAPDH
GAPDH+HDAC1
GAPDH+HDAC3
70kDa
50kDa
40kDa
35kDa
25kDa
20kDa
GAPDH-Kcro-FLAG 37kDa
Ctrl
pcDNA3.1-GAPDH
GAPDH+HDAC1
GAPDH+HDAC3
70kDa
55kDa
35kDa
HDAC3 50kDa
β-actin 42kDa
Ctrl
pcDNA3.1-GAPDH
GAPDH+HDAC1
HDAC1 62kDa
70kDa
50kDa
40kDa
35kDa
25kDa
GAPDH-FLAG 37kDa

Fig. 8m

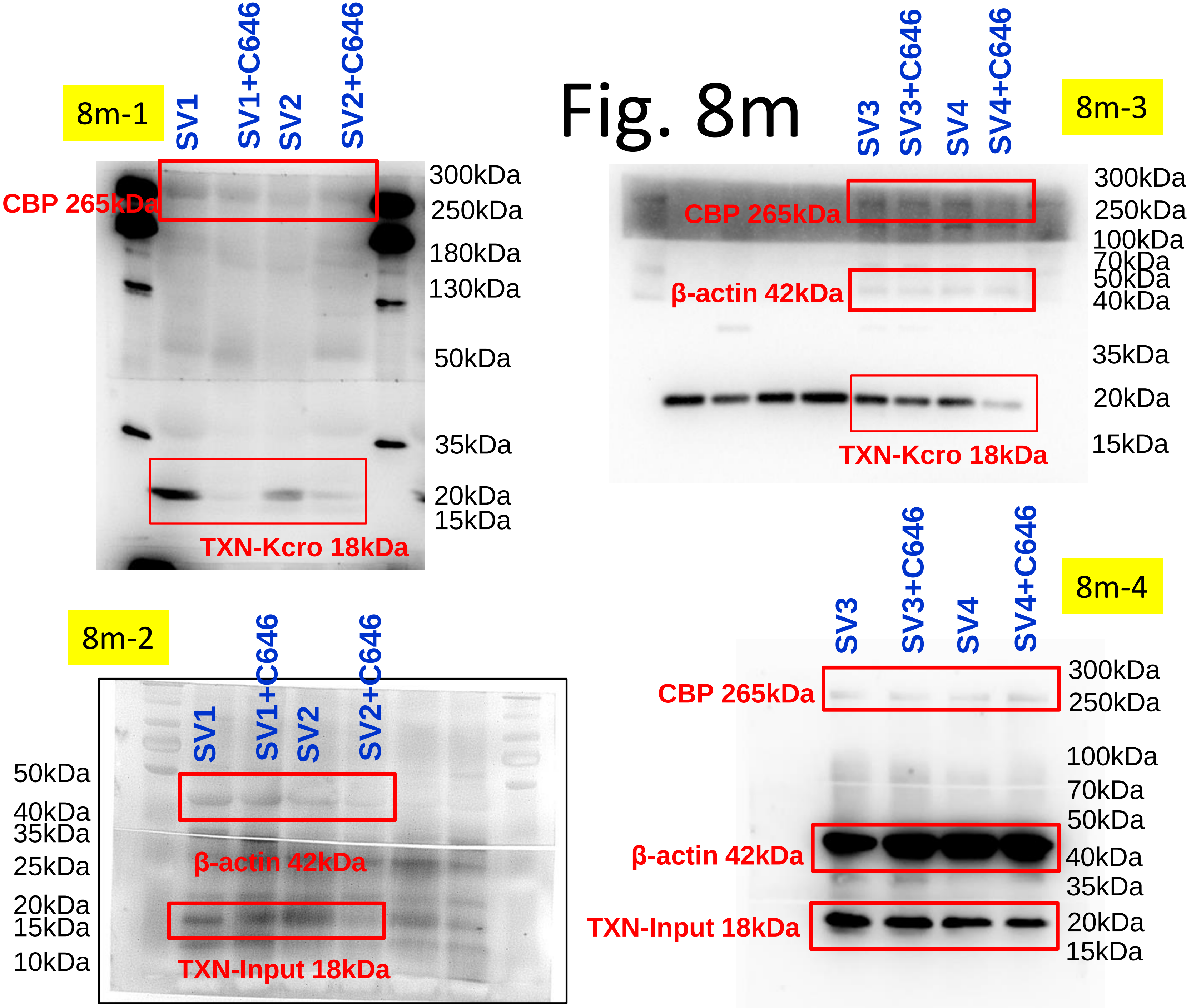

8m-1
SV1
SV1+C646
SV2
SV2+C646
CBP 265kDa
300kDa
250kDa
180kDa
130kDa
50kDa
35kDa
20kDa
15kDa
TXN-Kcro 18kDa
8m-2
SV1
SV1+C646
SV2
SV2+C646
50kDa
40kDa
35kDa
25kDa
20kDa
15kDa
10kDa
β-actin 42kDa
TXN-Input 18kDa
SV3
SV3+C646
SV4
SV4+C646
8m-3
CBP 265kDa
β-actin 42kDa
300kDa
250kDa
100kDa
70kDa
50kDa
40kDa
35kDa
20kDa
15kDa
TXN-Kcro 18kDa
8m-4
SV3
SV3+C646
SV4
SV4+C646
CBP 265kDa
300kDa
250kDa
100kDa
70kDa
50kDa
β-actin 42kDa
40kDa
35kDa
TXN-Input 18kDa
20kDa
15kDa

Fig. 8n

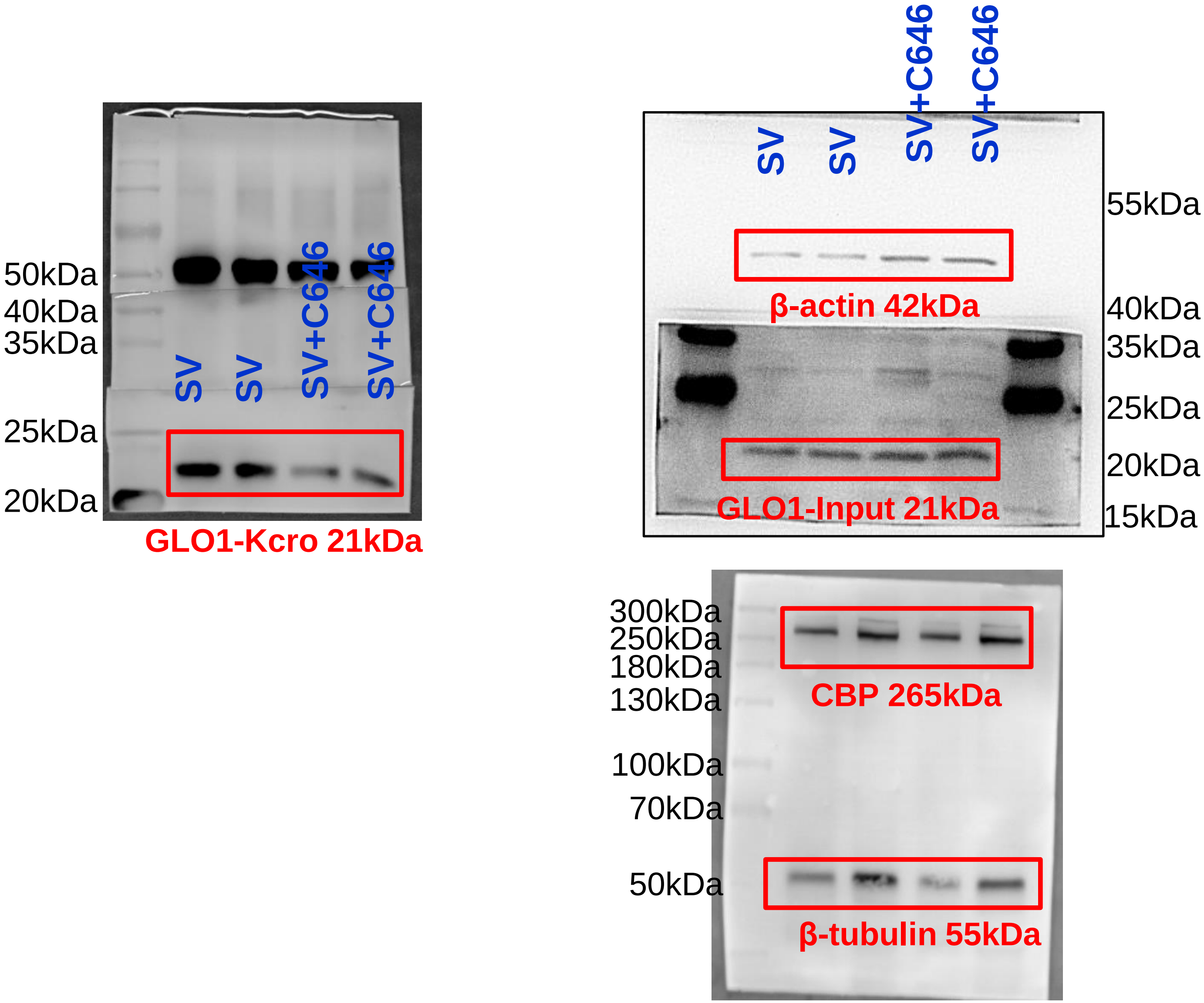

50kDa
40kDa
35kDa
25kDa
20kDa
SV
SV
SV+C646
SV+C646
GLO1-Kcro 21kDa
SV
SV
SV+C646
SV+C646
55kDa
β-actin 42kDa
40kDa
35kDa
25kDa
20kDa
GLO1-Input 21kDa
15kDa
300kDa
250kDa
180kDa
130kDa
CBP 265kDa
100kDa
70kDa
50kDa
β-tubulin 55kDa

Fig. 8o

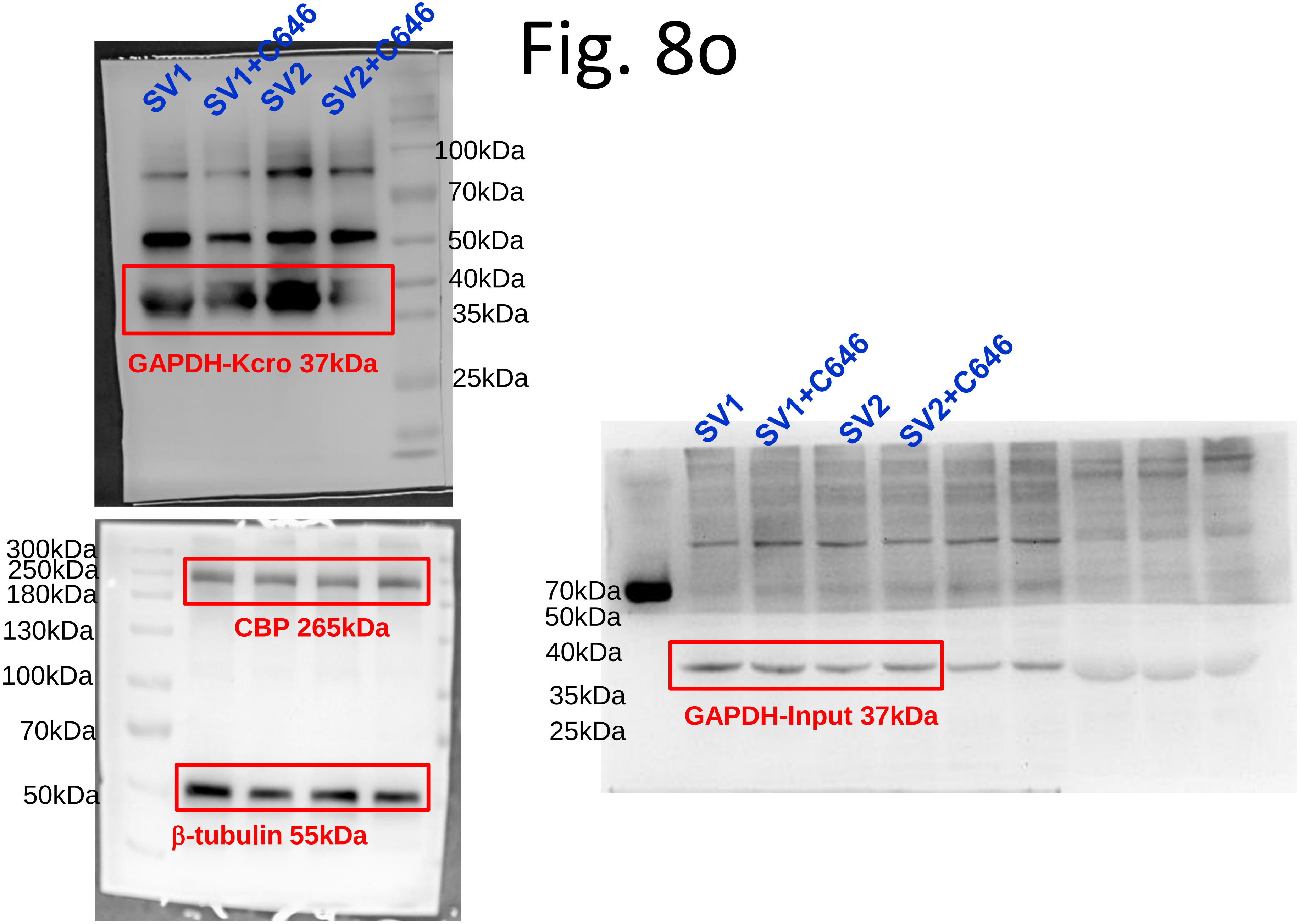
SV1
SV1+C646
SV2
SV2+C646
100kDa
70kDa
50kDa
40kDa
35kDa
25kDa
GAPDH-Kcro 37kDa
300kDa
250kDa
180kDa
130kDa
100kDa
70kDa
50kDa
CBP 265kDa
β-tubulin 55kDa
SV1
SV1+C646
SV2
SV2+C646
70kDa
50kDa
40kDa
35kDa
25kDa
GAPDH-Input 37kDa

Fig. 8p

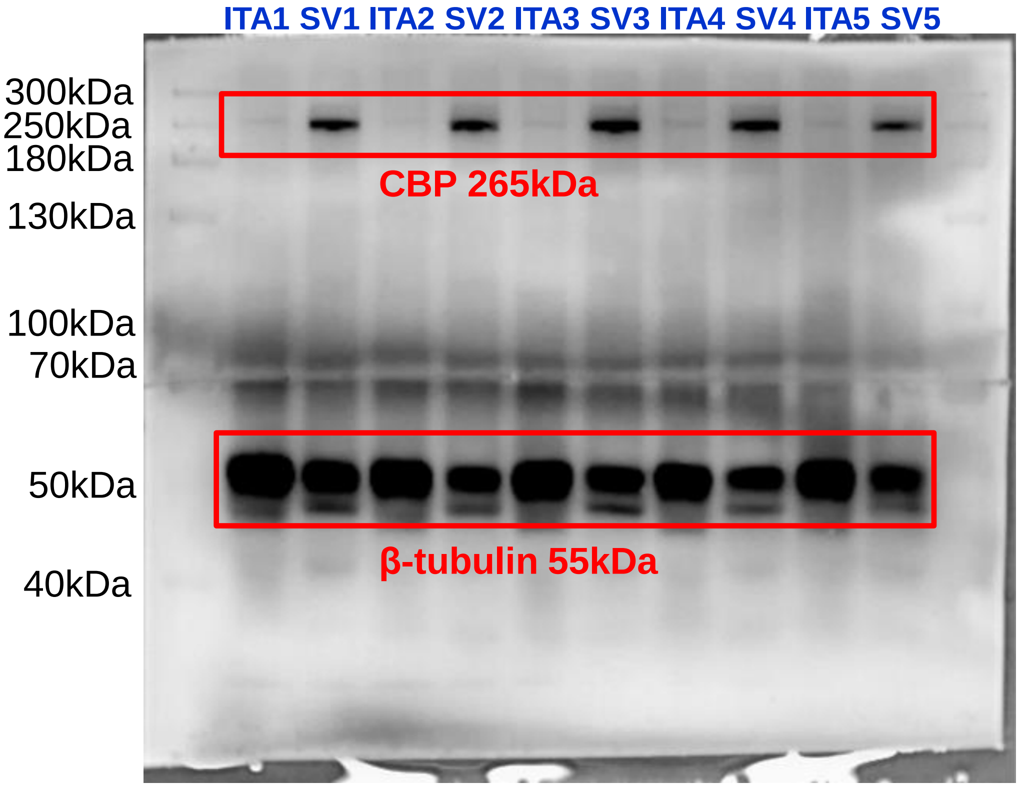
ITA1 SV1 ITA2 SV2 ITA3 SV3 ITA4 SV4 ITA5 SV5
300kDa
250kDa
180kDa
130kDa
100kDa
70kDa
50kDa
40kDa
CBP 265kDa
β-tubulin 55kDa
